# Holocene Closure of Lib Pond, Marshall Islands

**DOI:** 10.1371/journal.pone.0090939

**Published:** 2014-03-17

**Authors:** Conor L. Myhrvold, Fran Janny, Daniel Nelson, S. Nemiah Ladd, Alyssa Atwood, Julian P. Sachs

**Affiliations:** School of Oceanography, University of Washington, Seattle, Washington, United States of America; ETH, Switzerland

## Abstract

Well-preserved sediment from closed water bodies of atolls such as Lib Pond are rare opportunities to reconstruct the past regional climate, which pieced together across a latitude and longitude range identify the range of movement patterns of wider scale climate phenomena such as the Intertropical Convergence Zone (ITCZ) and El Niño Southern Oscillation (ENSO). We conducted the first physico-chemical survey of Lib Pond, a shallow, closed-water saline lake located on remote and difficult to access Lib Island in the Marshall Islands at 8° 18′ 48.99″ N, 167 22′ 51.90″ E in the Pacific Ocean, in July 2009. We performed a bathymetric survey, recorded salinity, dissolved oxygen, pH, and temperature profiles, monitored the tidal variability, and conducted a vegetation survey surrounding the lake. From bathymetric data we calculated the lake volume, which we used to estimate the lake's salt budget, and ultimately the residence time of water in the lake basin. We took a series of sediment cores from the lake, cores which indicate Lib Island's changing environment and climate. Radiocarbon measurements determined sediment age, and reveal significant mixing over the last 2 ka of deposition. We conclude that prior to 3 ka, Lib Island was an atoll with a central lagoon connected to the open ocean, which was then closed off from the open ocean to form the brackish system that exists today. We predict that the sediment accumulation in Lib Pond evident today will continue. As seawater is inhibited from exchanging with fresh water, Lib Pond will become a shallower lake with increasingly fresh water.

## Introduction

### Lib island

Lib, also called Ellep, is a coral island that is part of the Ralik Chain in the Republic of the Marshall Islands. The Marshall Islands are located between 4–14°N and 160–173°E, 181.3 million km^2^ of land area across 1,942 million km^2^ of ocean with a mean elevation 2 m above sea level. The majority of the Marshall Islands are low-lying partially submerged carbonate platforms surrounded by fringing reef, likely the remaining emergent portions of otherwise subsided atolls [1]. Lib Island today has a wet tropical climate, based on data from the nearby meteorological station on Kwajalein Atoll (58 km to the NE, 8° 43′N 167° 44′E) (Table 1, Table 2). Annual temperatures at the surface average 27.9°C (Table 1) and the prevailing winds are the easterly trade winds. Three-quarters of annual rainfall occurs during the wet season (June – Dec), influenced by the El Niño Southern Oscillation (ENSO) [1]. This precipitation distribution is consistent with the annual migration of the Intertropical Convergence Zone (ITCZ). In this regard, drought periods on Lib – inferred from decreased rainfall on neighboring Kwajalein – commonly follow El Niño events [2].

**Table 1:** Lib Island Profile Parameters.

Parameter	Value	Source
Land area	0.93 km^2^	RMI Embassy
Maximum width	2.12 km	RMI Embassy
Average annual temperature	27.9°C	RTS Weather Station
Average annual rainfall	2,540 mm/yr	RTS Weather Station estimate (“approximately 100 inches”).
Average Kwajalein monthly rainfall, 1945–2009	108 mm/month (January); 255 mm/month (July)	RTS Weather Station (converted from inches and rounded to nearest mm).
Lib Pond surface area	120,692 m^2^	This study (calculated in Mathematica from Google Earth satellite image outline).
Lib Pond surface area	117,212 m^2^	This study (calculated in ArcGIS from Google Earth satellite image outline).
Lib Pond perimeter	1658 m	This study (calculated in Mathematica from Google Earth satellite image outline).
Lib Pond perimeter	1728 m	This study (calculated in ArcGIS from Google Earth satellite image outline).

**Table 2:** Kwajalein Weather Data, July 21–25 2009.

Date	Min Temp (°C)	Max Temp (°C)	Precipitation (mm)	Thunderstorm?
July 21	26.4	30.4	0.254	No
July 22	24.7	30.5	16.00	No
July 23	24.2	30.6	26.67	Yes
July 24	24.2	29.3	47.50	No
July 25	25.0	30.6	8.13	No

Source: We used records from Kwajalein's (permanently based) weather station. http://rts-wx.com/data/climatology/daily_summary/2009/#table6

**Figure 1:**
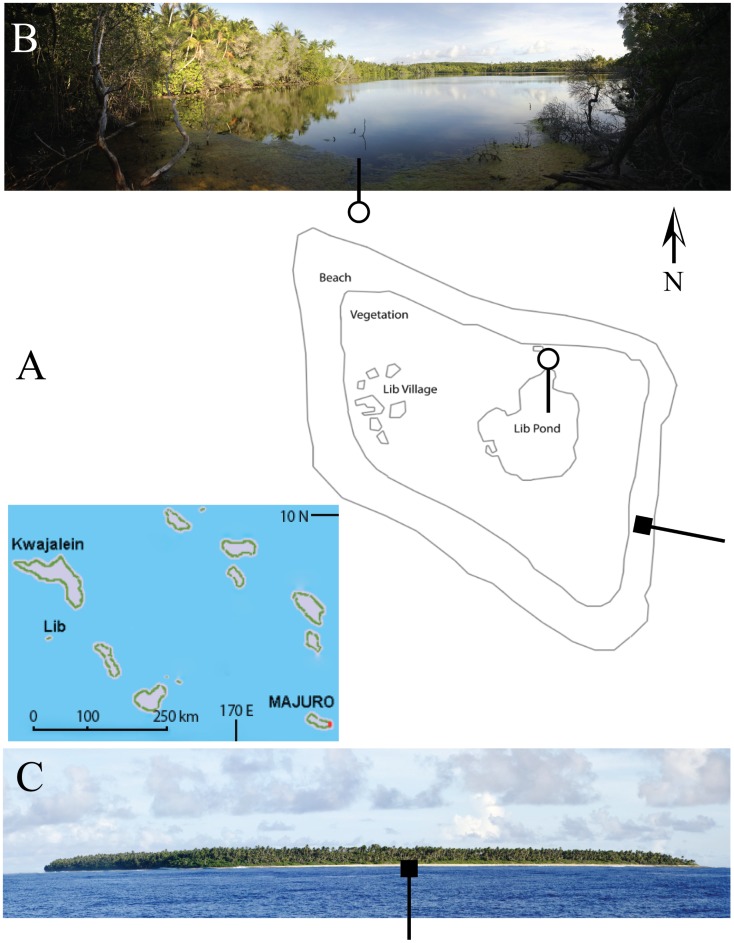
Lib Island. Schematic of Lib Island (A), with *stitched panorama* of Lib Pond lakeshore edge (B) and an offshore view of Lib Island on boat approach (C). The circle line symbol indicates the orientation of (B) on (A), from north to south. The square line symbol indicates the orientation of (C) on (A), looking from east to the west. Additionally, a regional map of the Marshall Islands with Lib Island is left center.

Lib Pond is located at the east end of the island; its closest edge is 150 m from the ocean beach (Fig. 1). A bathymetric survey had never been conducted at this remote site, and no hydrologic data existed for the lake system. The surrounding native forest around the lake's perimeter does not show manmade structures or human impact which would comprise the sediment chronology. Today Lib has 115 human inhabitants [3] living at the opposite end of the island to Lib Pond.

Lib Pond is a unique case study since it is one of 3 true islands in the Marshall Islands out of 29 main atolls, and the only location with a closed water body that might have sufficient sediment to reconstruct a 3 ka local climate history, based on our month-long Marshall Islands expedition which visited atolls and islands including Majuro, Arno, Ailinglaplap, Lib and Mejit. Here we describe the limnology of Lib Pond and develop a bathymetric map using a boundary slope fitting technique, which we have determined to be suitable where numerous depth waypoints cannot be taken (see Supp. Info S1 for more detail). We also report a series of chemical and physical properties of the lake, including salinity, pH, dissolved oxygen, temperature, water ^2^H/^1^H ratios, and sediment porosity. We use these parameters along with the bathymetric data, lake level, basin morphometry, and satellite imagery to calculate the flushing time of the system. When coupled with a description of the lake bottom sediments these data represent an important step towards characterizing the environment of this unique and remote location, and is as such the first scientific study devoted to Lib Pond [4].

## Results and Discussion

### Volume and depth

**Figure 2:**
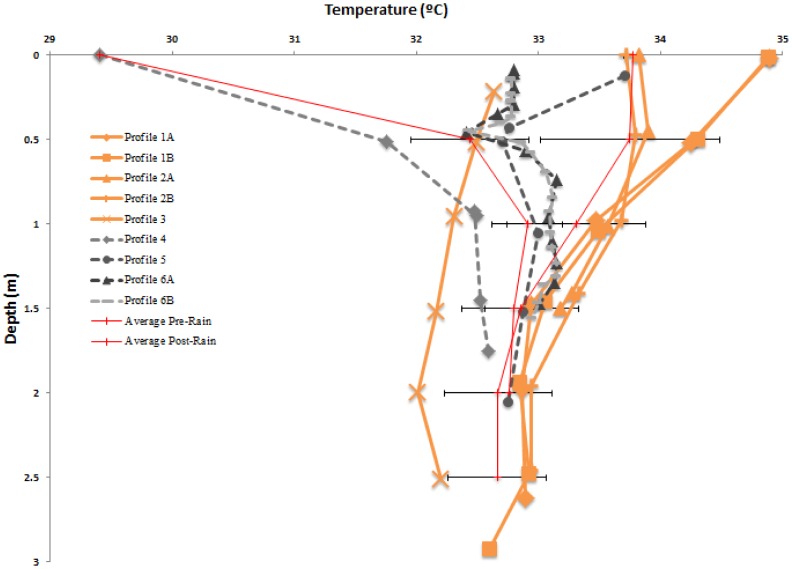
Water Temperature Profiles. Depth profiles of measured water temperature, July 21–23, prior to significant rain event (in orange), with July 24–25, following a significant rain event (in gray scale). Also displayed are pre-rain and post rain averages with horizontal errors bars representing the standard deviation of the average, on either side.

**Figure 3:**
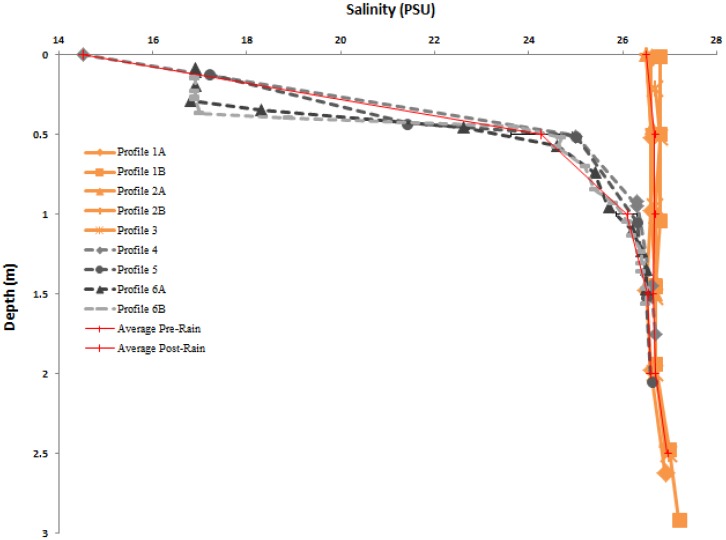
Salinity Profiles. Depth profiles of measured salinity, July 21–23, prior to significant rain event (in orange), with July 24–25, following a significant rain event (in gray scale). In the top meter of Lib Pond, there was a dramatic decrease in salinity following a series of freshwater precipitation events (Table 2, July 23–24). Also displayed are pre-rain and post rain averages with horizontal errors bars representing the standard deviation of the average, on either side.

**Figure 4:**
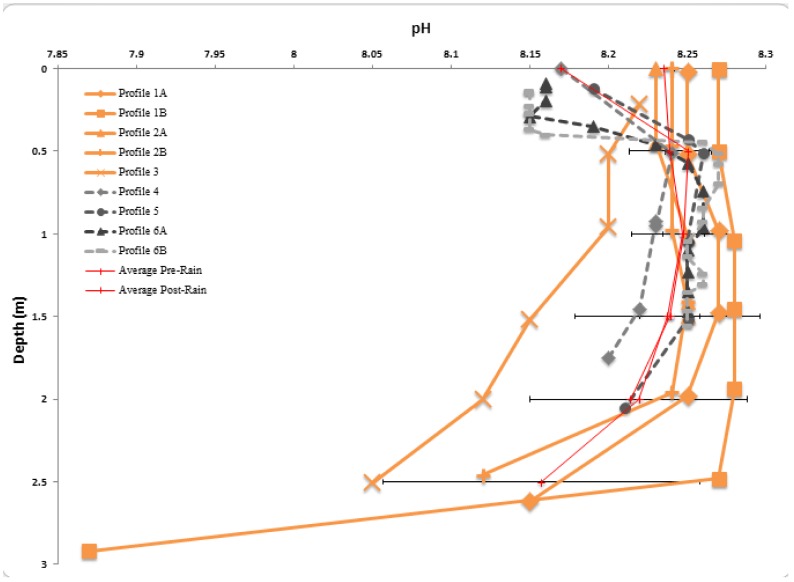
pH Profiles. Depth profiles of measured pH, July 21–23, prior to significant rain event (in orange), with July 24–25, following a significant rain event (in gray scale). Also displayed are pre-rain and post rain averages with horizontal error bars representing the standard deviation of the average, on either side.

**Figure 5:**
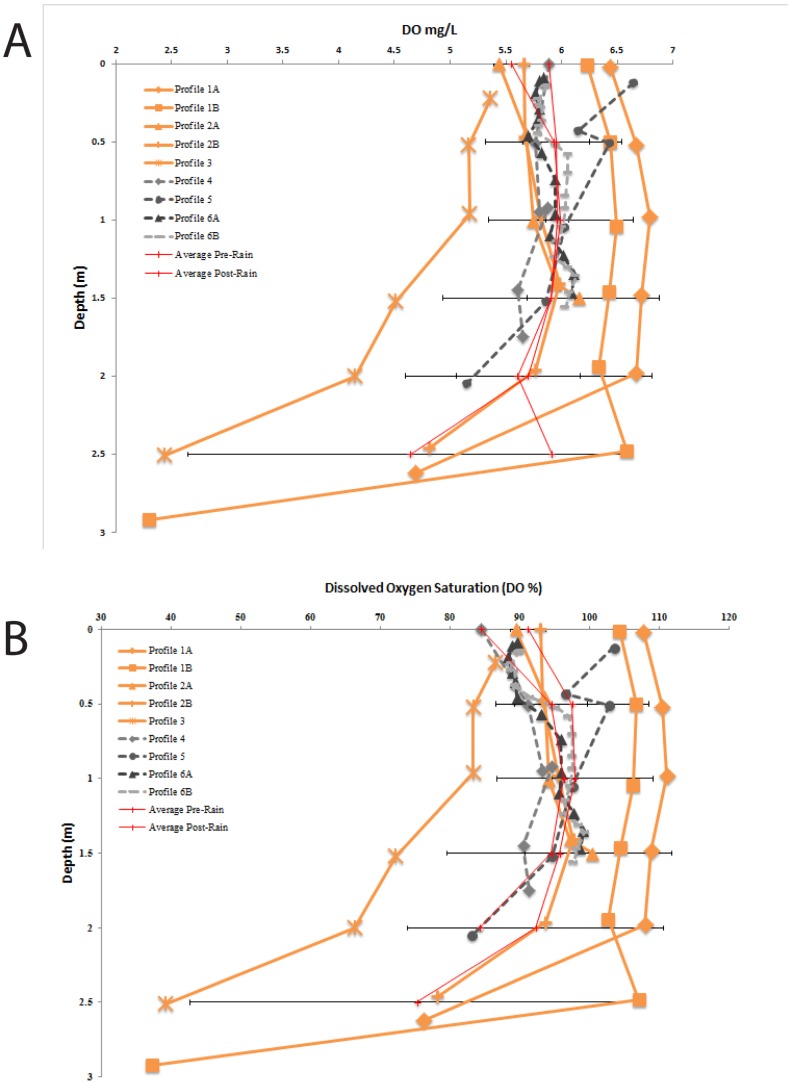
Dissolved Oxygen Profiles. Depth profiles of measured dissolved oxygen concentration (A) and percentage (B), July 21–23, prior to significant rain event (in orange), with July 24–25, following a significant rain event (in gray scale). Also displayed are pre-rain and post rain averages with horizontal errors bars representing the standard deviation of the average, on either side.

Lib Pond is a shallow lake with a depth of 0.2–3.3 m and mean depth of 2.3 m (see Fig. 1A for its shape). The bathymetric structure of Lib Pond is a shallow floodplain region in the southwest, with water < 1 m deep. Most depths in the lake center were within 0.5 m of each other, suggesting a relatively flat central bottom. The steepest depth gradients occurred along the eastern edge. Lib Pond's estimated volume depends on the variance in slope change across the bottom; the sharpness of the slope, which when summed across all depth changes can lead to meaningful volume differences. Lib Pond's volume is ∼135,000 m^3^ using a simple interpolation based on the original GPS and depth pairings. However, this estimate produces a bathymetric map with steep and jagged edges unrealistic for a lake. A more refined method which smooths out the bottom slope changes, a method which we call boundary slope fitting, followed by Delaunay triangulation, yields a volume estimate of ∼170,000 m^3^ (See Supp. Info S1 and Methods for additional details), a 35,000 m^3^ difference. We construct several profiles for each geochemical characteristic of the water in Lib Pond (temperature, salinity, pH, dissolved oxygen levels and percentages), before heavy rain and after rain (Figs. 2,3,4,5), which are described in detail in the Materials & Methods (Table 3).

**Table 3:** Water Sample Profiles.

Profile	Date	Time (hh:mm:ss) UTC/GMT +12	Depth range (m)	GPS coordinates
1A	21-Jul-09	15∶27∶49	0.02 – 2.62	8° 18.808′ N, 167° 22.942′ E
1B	21-Jul-09	15∶49∶14	0.01 – 2.92	8° 18.808′ N, 167° 22.942′ E
2A	22-Jul-09	15∶13∶13	0.00 – 1.50	*
2B	22-Jul-09	15∶33∶38	0.00 – 2.46	*
3	23-Jul-09	11∶07∶11	0.22 – 2.51	*
4	24-Jul-09	17∶39∶54	0.00 – 1.75	*
5	25-Jul-09	14∶21∶13	0.12 – 2.05	8° 18.873′ N, 167° 22.860′ E
6A, 10 sec	25-Jul-09	15∶07∶51	0.09 – 1.47	8° 18.830′ N, 167° 22.885′ E
6B 5 sec	25-Jul-09	15∶10∶41	0.14 – 1.56	8° 18.830′ N, 167° 22.885′ E

*No GPS data available.

### Tidal influence and flushing time

Lib Pond is mesosaline (20–50 psu, see Supp. Info S1), polymictic, alkaline and eutrophic. No surface connection to seawater exists, but based on tidal and chemical measurements – which varied little compared to ocean tide ranges – we believe Lib Pond has a limited subterranean ocean exchange.

We observed lake level fluctuations during 5 sampling days. A substantial rain event occurred on July 23–24. According to pre-rain lake level changes an estimated influx of seawater occurred in 21.1 hrs, corresponding to the increasing tide over this interval, which increased lake level −1.1 cm to +2.0 cm relative to the initial gauge reading. Data indicate a tidal range of 3.1 cm and a period of 42.2 hrs.

Lib Pond's tidal range (1.61 m) is 1.9% of the ocean range, according to the July 21^st^ Kwajalein tide table (assumed comparable to Lib Island, see Supp. Info S1). Tidal frequency within the lake (0.57 period d^−1^) is also dampened to 30% of regional ocean tidal frequency (1.91 period d^−1^). In other words, Lib Pond's water level moved little compared to the ocean's water level across the same set of tides.

The residence time of the lake water has a period of 31–34 d^−1^, using measurements of reservoir salinity (26.72 psu) applied to a mass balance model of dissolved constituents. We used a tidal prism model of a well-mixed estuary, with inputs of approximate daily tidal range of 3.1 cm d^−1^ and summer P-E records over the geographic region of +10 mm d^−1^. Thus, we estimate Lib Pond lake water has complete turnover about every month.

**Figure 6:**
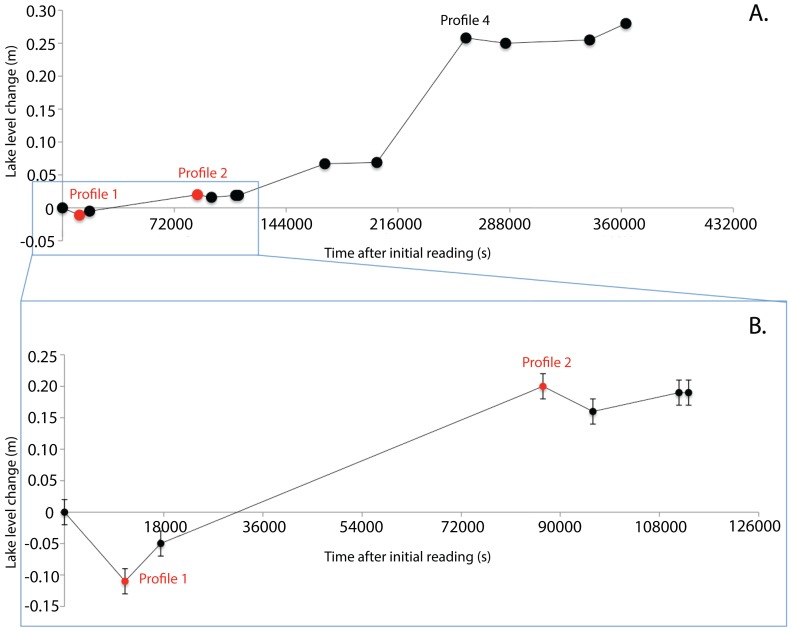
Lake Level Readings. Panel A: Recorded lake levels spanning the duration of sampling time, with several listed Profiles for reference. Values are reported in cm and on the time scale of seconds following initial lake level reading. Dates are listed above two of the plotted points. Panel B: Lake level readings between July 21–22. Red points correspond to values selected for estimation of lake tidal range.

Lib Pond's water temperature (Fig. 2), salinity (Fig. 3), and pH (Fig. 4) at the surface varied considerably over short timescales (Fig. 6) due to episodic rain (see Kwajalein Rainfall Data in Table 2), while dissolved oxygen levels did not meaningfully change. Rainfall did not affect deeper geochemical measurements of the lake.

### Dissolved oxygen (DO)

Dissolved oxygen (DO) for all depths was between 2.30±0.02 mg/L and 6.79±0.07 mg/L among profiles taken between July 21^st^ and 23^rd^. The lake was supersaturated on July 21^st^ down to 2 m depth, with a mean saturation of 107.1±2.6% spanning a range of 102.8±2.4% and 111.1±2.4% (Fig. 5).

Below 2 m, a rapid decrease in DO was observed, and approached hypoxia at 2.92 m (37.3±2.4%). On July 22^nd^, DO fell, ranging 89.48±2.4% to 100.51±2.4% in the top 2 m of the water column with a 94.8±3.0% mean. DO saturation also decreased at depth, reaching 78.23±2.4% at 2.46 m. DO saturation in Profile 3 was consistently lower than in previous profiles (but it was also collected at a different time of day than the other profiles). Saturation was 86.64±2.4% at the surface (0.22 m) and decreased with depth to the near-hypoxic level of 39.3±2.4% at 2.51 m.

After the rain event on July 24–25, oxygen was undersaturated throughout the water column, with the exception of 2 supersaturated readings from Profile 5 toward the surface at 0–1 m. Except for the Profile 5 readings near the surface, DO% was slightly lower in the top 0.5 m (range of 84.5±2.4% to 91.5±2.4%, mean of 89.1±1.5%) than in the remainder of the water column. The DO% ranged 91.2±2.4% to 99.4±2.4% and averaged 96.1±2.6%. We did not take post-rain profiles below 2 m in depth.

Lib Pond's water column was nearly saturated or supersaturated in DO which suggests high biological production. The greenish-brown, turbid water in the lake and abundant floating algal mats indicate high phytoplankton biomass. The sharp decline in % DO saturation that occurred below 2 m depths, trending towards hypoxic conditions, is likely an indication of the oxygen electrode recording measurements at or below the poorly constrained sediment-water interface. The discrepancy among % DO profiles taken before the rain could be explained by inconsistency in sampling time, as phytoplankton produce more oxygen in sunlight during the day. Profile 3 was collected several hours before Profiles 1A/B and 2A/B, before noon, when irradiance is highest. The lower % DO saturations of Profile 3 could therefore be from lower solar irradiance.

### Temperature

Profiles taken prior to the rain event revealed slightly decreasing trends of temperature with depth. Lake temperature ranged 32.6–34.9±0.2°C among all profiles except Profile 3 (taken earlier in the day). The total range of all profiles is > 2°C (Fig. 2).

On July 24^th^, following the rain event (Profile 4), at 1.75 m temperature decreased to 29.41±0.1°C at the surface and reached 32.59±0.1°C, consistently lower at corresponding depths than the pre-rain profiles also taken in the afternoon. On July 25^th^, 2 days after heavy rain, surface temperatures increased and approached pre-rain temperature values and becoming uniform within the water column, averaging 32.92±0.2°C.

The temperature profiles preceding the large rain event represent the differential solar irradiance in a given diurnal cycle (e.g. lower temperatures in the morning when Profile 3 was measured than the afternoon when Profile 1A was measured). Lower surface temperatures in Profiles 2A/B on July 22^nd^ (compared to Profiles 1A/B on July 21^st^) may have been due to more lower solar irradiance caused by cloud cover or turbulent mixing because of higher wind stress, equilibrating the surface with the cooler air (27.9°C). Post-rain profiles reflect the cooler temperature of the rainwater introduced to the system, a previously documented phenomenon [5]. During the rain event, cloud cover increased, so lower solar irradiance also could be an additional cause for depressed temperatures.

### Salinity and hydrology

Before the rain on July 23^rd^, the lake was mesosaline: salinity ranged 26.5–27.2±0.3 psu, with a mean value of 26.72±0.2 psu. Afterward, the 3 m water column became highly stratified (see Fig. 3, Profiles 4,5,6A,6B); salinity ranged from 14.5–26.7±0.3 psu, with the lowest salinity readings obtained the day following the rain shower at the surface and increasing with depth to 1 m where pre-rain values were reached. A surface mixed layer between 0–0.3 m appeared to have developed by July 25^th^, with a uniform salinity of 16.9 psu (Fig. 3).

Salinity was relatively uniform with depth. We therefore classify the lake as warm polymictic. The mesosaline lake water of 26.72 psu suggests exchange with 34.3 psu ocean water surrounding Lib Island. This exchange is supported by tide gauge readings which indicate oscillatory water level fluctuation.

The lake's internal mixing rate must be relatively rapid because the salinity in the top meter of the 3 m pond depth changed almost immediately after significant rainfall. As previously mentioned, uniform salinity profiles prior to the rain event became highly stratified, as the freshwater input mixed with the existing saltwater the 24 hrs after the rain. This mixing is likely driven by wind-generated turbulence that penetrates to the shallow lake bottom [6]. The estimated rainfall of 21 cm for July 23–24 based on salinity profiles, closely matches that estimated from tidal readings (24 cm), suggesting that the flushing rate is relatively low compared to internal mixing.

To calculate the lake water residence time, we assumed that the lake system is in steady state [7]. Although this was not the case, the fluctuating water levels observed during sampling imply that the input of tidal floodwater and seasonal daily average P-E are small compared to the overall water volume of the lake and can therefore be ignored for the flushing time calculation. Another assumption in the mass balance model is instantaneous mixing of inputs into the system during flood tide. In the case of Lib Pond this assumption is well justified given the well-mixed salinity profiles before rainfall occurred (Fig. 3).

Model parameters do not account for incomplete exchange of inflow during the tidal cycle, as well as floodwater being a mixture of ebb flow and seawater. These factors may result in an underestimation of total flushing time [7]. Further uncertainty in turnover estimates owe to the few data points from which the flood tide input was extrapolated. However, the independent consistency of lake turnover – calculated from empirically measured lake chemistry (salinity mass balance) and again calculated from tide gauge readings – suggests that the flood tide data we did get is sufficient. The present-day hydrology of the system is summarized in a box diagram in the Supporting Information (Supp. Info S1).

### pH

Prior to the major rain event, lake pH was 7.87–8.28±0.2 and was generally constant with depth, averaging 8.26±0.1 until 2 m depth. Below 2 m the pH decreased with depth (Fig. 4). Profile 3, measured a few hours earlier in the day on a different date, yielded consistently low pH readings relative to the other 4 profiles; this profile ranged 8.22–8.05±0.2 from 0–2.51 m depth, following a decreasing trend with depth.

The lake pH after the rain event was slightly higher (8.15–8.27±0.2 down the water column). Minimum values occurred toward the surface between 0–0.5 m, while relatively uniform maximum values occurred from 0.5 m to 1.5 m depth. Profiles 4 and 5 extend below 1.5 m, where pH decreased slightly with depth. A mixed layer appears to have developed between 0–0.4 m, with a relatively uniform pH averaging 8.16±0.1, most pronounced in Profiles 6A and 6B.

The pH depth profile trends are moderately positively correlated to DO saturation (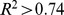, see Supp. Info S1). We expect this since higher photosynthesis rates increase pH via CO_2_ consumption. In tropical lake environments, rapid photosynthesis stimulated by high solar irradiance during the day can elevate pH by up to 2 units in just several hours [6].

### Vegetation and shore

Lake margins were generally low angle, although steep embankments were found along the northwest edge. The lake shore was comprised of a dark, organic-rich material overlain by abundant terrestrial plant debris. Dense and diverse vegetation, largely coconut palm and mangrove, persisted to the lake edge. Floating green algal matter (similar in appearance to, and likely similar in composition to Christmas Island [8]) ran along the lake perimeter as well as the offshore lake surface and thick, brown, unconsolidated organic matter protruded from the lake's southwest margin towards its center (observed on site and visible from satellite imagery.) The river channel visible in the southwest corner could be detected from the lake contours. We did not see any evidence of human alteration of the lake system. However, near the local village there was small scale subsistence crop agriculture.

### Sediment cores and stratigraphy

**Figure 7:**
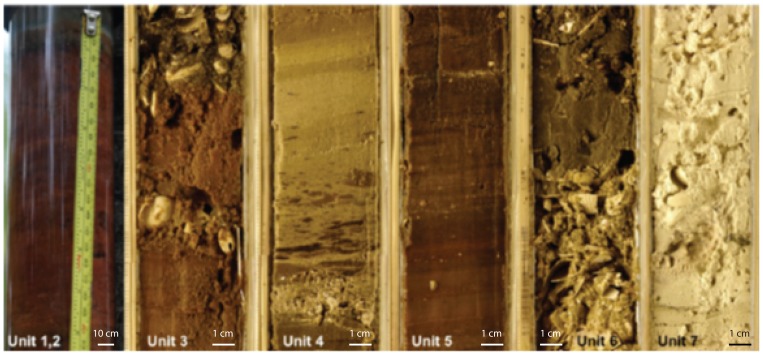
Lib Pond Sediment Unit Examples. Representative images of distinct stratigraphic units observed in sediment cores, Units 1–7. Unit 1 and 2 appear in the same image. Unit 1 is the 1–2 cm green layer above the underlying red material, Unit 2. Relative scales have been added to show how much real-world space each image represents.

The nearly 8 m lake sediment core exhibited stratigraphic variability, with substantial variability also observed between the cores (Fig. 7 for Units, and Supporting Information S1 for more detailed pictures of the actual sediment cores). The upper 2 cm consisted of flocculated green algal material (Unit 1). This was underlain by 30–100 cm of unconsolidated, rust-colored gelatinous organic material, possessing a sulfidic odor. Thin, lightly tinted laminae were observed along this sequence (Unit 2). Between this sequence was a diffuse chromatic boundary layer and an underlying brown-to-tan layer spanning 50–70 cm and persisting until 170 cm deep. At 170 cm, a diffuse boundary layer marked the emergence of a poorly sorted sequence of dark-brown-to-tan colloid with intermittent presence of bivalve shells and other carbonate fragments, proceeding until 0.26 m. Bivalve layers varied in both length and distribution between cores (Unit 3). The dominant bivalve species was *Ctena Bella*. A thinly-bedded to laminated sequence of generally dark brown material followed, more consolidated than the surficial laminated sequence. This layer spanned 0.26 m to 0.33–0.35 m in the piston cores (Unit 4).

Almost no bivalve presence was apparent and sediment diffusely transitioned to a sand layer sequence 20–25 cm in length. The lower region of this sequence was poorly sorted, containing components of variable sizes and densities, resembling underlying layers in color and apparent grain size (Unit 5). Intermittent brown silt and sand sequences containing bivalves persisted until 0.41–0.44 m deep (Unit 6), in which the transition was made to lagoon sediment (Unit 7). Coral basement was reached at 0.73 m.

Changes in sediment stratigraphy (Fig. 7) indicate deposition and inherently a change in the physico-chemical environment of the system during accumulation [9]. Though cores generally presented the same progression of facies, a number of the major sequences compared between cores appear at different depths, and persist for different lengths (see Supp. Info S1). This could be due to sediment compression upon collection, given the high water content in the sediment (e.g. Unit 2). Discrepancies could also reflect the heterogeneity of the basin's morphometry, whether collected on a basin slope or near the basin's maximum depth [10].

Unit 1 is suspected to be a living microbial mat, growing on the sediment water interface (see Supp. Info S1). Low-salinity lakes on Kiribati with high ocean-to-lake exchange also exhibited thin green alga layers on their sediment surfaces [8]. This material could also be recently deposited organic matter from the water column, since it closely resembles the floating algal mats observed on the lake's surface in physical appearance.

Unit 2 suggests decay of accumulating mat material, because microbial mat decay liberates cyanobacterial carotenoid pigments, giving the previously green material (like in Unit 1) a red-brown hue [11]. The lamination observed indicates an absence of bioturbation. This is supported by the observed oxygen-deplete conditions near or within the sediment, an inhibiting factor for benthic macrofauna [10]. The white-pink laminae observed could be calcium carbonate precipitates, formed by microbial mineralization. Lake enrichment of Ca^2+^, HCO_3_^−^ and CO_3_^−^, inferred by surrounding carbonate island sediment and oceanic communication, creates conditions close to CaCO_3_ saturation levels, which means changes in water chemistry can cause calcite precipitation. Increased photosynthesis, positively correlated to pH in the system, decreases the solubility of calcite, facilitating precipitation. While the decomposition of organic matter releases CO_2_ and increases calcite solubility, the net precipitate accumulation may be observable because of the microbial matrix created in the mat sequence, which has been shown to absorb to calcite crystals, supporting precipitate formation, termed organomineralization [12]. The water column's high bioproduction and pH conditions observed could support this CaCO_3_ precipitation. The alternating darker layers are likely due to biolamination. Burial can occur from abrupt interruptions in growth of the benthic mat population [11], most likely due to drastic water chemistry changes from intense episodic rainfall. Further, these laminations may reflect dramatic annual oscillation between wet and dry seasons observed in the Marshall Islands, tied to latitudinal ITCZ migration. Lamination in Unit 2 may also signify the region's interannual precipitation changes, most likely due to ENSO patterns. Microbial mats have also been linked to basin closure of lakes [8], and this thick mat sequence in the surficial sediment may explain our observation of minimal water exchange with the ocean through groundwater seepage.

The deposition of the highly variable sediment sequence, Unit 3, suggests a period in Lib Pond's history of periodically and dramatically changing lake characteristics. The dark coloration and fine-grained material persistent throughout the sequence is likely microbial mat growth, as cyanobacteria can grow in a wide range of salinities, from brackish to greater than 300 g/kg [13]. The presence of bivalves in the sediment, however, is closely tied to basin salinity. The dominating bivalve species has been identified as *Ctena bella*, from the family *Lucinidae* which are obligate halophiles, only occurring in saltwater (see certification in Supp. Info S1.) *Lucinidae* are largely known to inhabit marine environments, in benthic zones of normal ocean salinities [14]. The intermittency of bivalve layers is likely linked to changes in lake conditions such as increased ocean exchange. Sea level changes would influence surface connection to the ocean as well, through the possible creation or closing of a channel, and its influence on tidal transmittance through groundwater. Again, observed variability is likely due to ENSO events, since they are the driver of interannual sea level anomalies in the region [15].

The underlying Unit 4 sequence is composed of dark laminated material, with light dispersed lightly tinted laminae, and scant evidence of benthic faunal. The fact that Unit 4 mostly resembles Unit 2 means that environmental conditions were likely similar during the accumulation of these two sequences. If this was true, sediment accumulation and diagenesis effects on organic-rich sediments could account for the greater degree of consolidation and darker coloration compared to Unit 2 [16].

Unit 5 likely suggests a relatively abrupt and energetic event leading to the observed deposition of sandy material into the basin. This is substantiated by the poor sorting at the lower region of the sequence, and the presumed entrainment of underlying mat material, indicated by the regions of dark sediment portions dispersed within the sand layer. This may have been the result of a severe storm event that triggered over wash.

Unit 6 suggests a period of intermittent allogenic and endogenic sedimentation, with alternating bands of dark, likely microbial accumulation as well as carbonate sand deposits containing bivalve shells. From this sequence of continuous fine sand overlaying Unit 7 (the layer of presumed lagoon deposit) we infer an infilling of a previously existing lagoon or inlet on Lib Island.

### Lake variability due to rain event

We report salinity, temperature, pH, and dissolved oxygen both before and after significant rain, and tide gauge readings at the same time of day before and after the event indicated a 23.8 cm lake level net change. Accordingly, we estimate Lib Pond received 21±5 cm of rain on July 23^rd^ – 24^th^, 2009. Rain events occurring during sampling resulted in considerable lake chemistry variability over a 24-hr period. The extensive heterogeneity observed in lake sediment stratigraphy suggests dramatic changes in hydrologic conditions over longer timescales as well. The slight decrease in DO saturation seen in surface waters after the rain event could be from the contrasting chemical and physical properties of precipitation relative to that of the system. In particular, the rainwater lens likely has lower dissolved inorganic carbon and nutrient concentrations required by photosynthetic reactions [17], leading to reduced oxygen production.

### Cause of shallow flood plain

The current floodplain configuration is likely the result of wind-driven advection of floating vegetation and soil to the leeward side of the lake, where it sinks and accumulates, filling the basin in from the leeward to the windward side (prograding).

### Bathymetric map construction

We fit exponential functions to the lake slopes of linear depth transects and apply those functions to nearby shoreline regions without GPS depth data, in what we call boundary slope fitting. The method yields a bathymetric map of a body of water, using several depth transects, a satellite image and commercially available software (ArcGIS, Mathematica).

The concept is a way to model the lake bathymetry in unknown regions based on known adjacent depths and topographic information not reflected in that data, yet still relevant to the bathymetric map construction: the lake perimeter has a depth of zero, contains a small number of shoreline types which can be determined from looking at transects (such as a sloping beach or a rapid drop off) and that a gradual transition ensues from these shoreline types to deeper regions. In this manner, unknown regions of the lake are assumed to have similar properties to regions with depth data.

Since all added depth points are near the lake edge and these locations are shallower on average than the central parts of the body of water, boundary slope fitting does not affect the resulting volume calculation as much as the method of [18], where central (deep) regions of the lake are modeled in addition to shoreline regions. The added points give boundary values, which affect the volume slightly but primarily create reasonable lake slopes and correct knife edge-ridges. This method also improves accuracy for water bodies with irregular shapes and depth patterns as it does not require a geometric shape such as [18] (to make transect depth profiles).

### ITCZ implications

Paleoclimate data is of great interest in the present era of global warming. Establishing the natural variation bounds of past climate increases our understanding of the current global climate system, which aids in anticipating future climate changes related to greenhouse gas forcing.

Climate variability within the equatorial Pacific is essentially confined to patterns of rainfall. The Intertropical Convergence Zone (ITCZ) is the latitudinal region that Earth's tropical rain band annually oscillates across, a narrow band of deep convection and heavy precipitation. The annual migration of this rain belt is responsible for wet and dry seasons in the Equatorial Pacific basin. Presently the ITCZ spans a latitude range near but north of the equator (∼3–10°N) [19].

Interannual climate patterns (i.e. precipitation) near the equator are largely influenced by El Niño Southern Oscillation (ENSO) cycles. ENSO induces large sea surface temperature (SST) variations, especially in the east equatorial Pacific, which are responsible for the observed precipitation changes [20].

Studies of paleoclimate indicate that the Intertropical Convergence Zone (ITCZ) reached its southern-most position within the Holocene during the Little Ice Age (LIA). Titanium and iron concentrations in ocean sediment from the Cariaco Basin in the south Caribbean at the northern boundary of the ITCZ [21], in addition to hydrogen isotope (^2^H/^1^H) sediment core data from closed water bodies of tropical Pacific islands and atolls from a latitude range spanning the ITCZ as well as above and below it [22], suggests that the ITCZ itself has shifted: 5°S from the Medieval Warm Period (MWP) to the Little Ice Age (LIA) and 5°N from the MWP to the where it presently resides [19]. Each of these studies only directly supports regional movement but they and others concomitantly portray a broader picture – one relevant to climate modeling – of global ITCZ movement, since the regional movement directions studied thus far have been consistent with each other.

However, there have been until recently very few Holocene climate records from inside the tropical Pacific basin, the region in which ENSO is generated, as well as the meteorological core of the ITCZ, records which would vastly enhance our current understanding of tropical convection patterns through time. Geochemical data from the present day Lib system, in addition to the sediment cores we collected, provide a snapshot of the Lib region's climate today in addition to its past, that can serve as a benchmark to compare against possible future ITCZ movement.

## Summary and Conclusion

We describe Lib Island's lacustrine body in terms of geology, morphometry, ecology and physico-chemistry, combining water property measurements of salinity, pH, temperature, and dissolved oxygen (DO) with water level fluctuations and macroscale stratigraphic observations to generate a physico-chemical overview. Using GPS data and computer modeling we created a bathymetric map of Lib Pond with estimated ranges for surface area, perimeter and volume using two methods (boundary slope fitting, Delaunay Triangulation). These methods can be applied to any shallow body of water, particularly where data intensive bathymetric techniques and multispectral imaging are unavailable.

Based on a simple age model from sediment core dates and the coral bottom (see Supp. Info S1), prior to several thousand years Lib Island was a coral atoll with a lagoon open to the ocean. Sediment accumulation changed the island morphometry enough to create a closed body of water. As the water contribution increasingly came from precipitation the salinity decreased to the point where Lib Pond is brackish today. The sediment of Lib Pond changes dramatically during the several thousand years of accumulation. These changes are likely due to changes in lake chemistry and biology caused by the sensitivity of Lib Pond to interannual perturbations including ENSO events and possibly the ITCZ's annual oscillation range change during this time period [19] [22] as well.

## Materials and Methods

Permission for fieldwork on Lib Island was granted by Ingrid Ahlgren of the Historic Preservation Office of the Marshall Islands. The individuals in this manuscript have given written informed consent (as outlined in the PLOS consent form) to publish these case details.

### Kwajalein weather and climate data

We used records from Kwajalein's (permanently based) meteorology station as a proxy for Lib Island (Table 2). We also show the average annual rainfall of Kwajalein compared against the general regional rainfall trend, which correlates with the Southern Oscillation Index (SOI), a pressure difference measurement which serves as an indicator for El Niño/La Niña cycles (see Supp. Info S1).

### Fieldwork

Field sampling was performed July 21–25, 2009 on Lib Island's single lake, Lib Pond (centered at 8° 18.81′ N, 167° 22.88′ E). A series of intense rain showers took place starting July 23^rd^ through July 24^th^. Weather data from the Kwajalein over the same time period (seen in the Supp. Info S1), indicates a thunderstorm on July 23^rd^, with significant rainfall on July 23^rd^ and 24^th^ like on Lib; although tropical precipitation is highly spatially variable, and it is not surprising that precipitation occurred on July 22^nd^ on Kwajalein but was not observed on Lib.

The equipment used for the survey is detailed in the Supp. Info S1. We used a small inflatable row boat to navigate the lake surface. Our multiprobe took measurements of temperature, salinity, dissolved oxygen (DO mg/L), dissolved oxygen percent saturation (abbreviated as DO % sat), and pH (Table 3).

Intact sediment–water interface cores were recovered with a universal core head sediment sampler (U-core, manufactured by Aquatic Research) and sub-sampled on site in 1 cm intervals and frozen the same day. Longer cores were taken with a Livingstone-type piston corer (Geocore).

Water depths were coupled with GPS coordinates at each depth location to create 8 linear lake transects. We recorded tide gauge readings throughout the sampling period at different times of day. Tidal fluctuations were negligible in comparison to the baseline depth values over the sampling time period.

We collected vegetation samples of the 12 most dominant species in Lib Pond's surrounding perimeter, which can be used to determine the sources of lipid biomarkers to the sediment.

More details are located in the Supporting Information (Supp. Info S1).

### Projecting lib pond in a coordinate system

Since the Lib Pond outline was not able to be traced with GPS coordinates in the field due to thick vegetation, the satellite image of the lake was directly input into Mathematica along with the collected data (GPS locations coupled with bottom depths). Geodetic calculations project the GPS data on a Lambert-Azimuthal Projection of the satellite image. The Lambert-Azimuthal projection is used because it keeps the area constant, i.e. there is little distortion. (Although at only several hundred meters across, practically any map projection would do for Lib Pond as projection distortions are small across a small area.) For the projection image, see the Supporting Information (Supp. Info S1, Satellite Image Alignment section.)

### Lake dimension calculation

To compute the lake dimensions, elements of the lake discovered through fieldwork and a satellite image were combined. A high resolution satellite image (Google Earth) provided GPS coordinates of the lake outline along with general bottom types, most notably confirming the presence of a very shallow section.

### Accounting for the floodplain

One large portion of Lib Pond inaccessible to the boat is noted by both field observations and the satellite image to be an extremely shallow floodplain. Another assumption is that the entire floodplain is well approximated by the average depth we measured (before the boat could not go any further due to too-shallow depths). 0.2 m depth points were placed at the floodplain boundaries to ensure a uniform depth value for the entire floodplain.

### Boundary slope fitting method

Fieldwork depth and GPS data points were assembled together and from that, significant features were interactively traced onto the image, such as the lake outline. The depth of the lake outline (composed of connected points) is effectively treated as zero, as that is the value each boundary point assumes. Next, the lake depth points are interpolated to create a 3D bottom profile. Known depths are assembled into linear combinations of depths (transects). Lake regions sharing shore type and bottom conditions were matched (see Supp. Info S1.) The known lake slope information is mathematically taken into account by fitting a smoothed curve to the transects. There were 5 shoreline type fits from nearby transects (called edge types). On each type, a nonlinear least squares fit was performed to the rational exponential function:
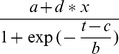
(1)


The fitting procedure determined the parameters 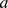, 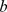, 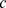 and 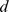 with excellent fit quality (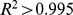). In every case, the slopes of the lake transects is well approximated by an exponential fit. The resulting rational exponential functions are then used to create shore profiles projected out on normal vectors from the edge of the lake in regions of the lake where the bathymetry was undetermined. Lake slopes are extrapolated and extended along the shoreline, using the relevant shoreline type. Depending on which shore type was involved, the shore vectors were projected out 10–60 m from the lake edge. No hypothetical shore points were projected in areas that already had dense accumulations of data points. Edge types 2–5 each represent a different shoreline type (the first edge type is the uniform value for the shallow floodplain). Additional details and descriptions are described in the Supporting Information (Supp. Info S1).

### Lake volume calculation

A triangulated surface model of the lake was computed from a Delaunay Triangulation of the original data points. The concept of its application is simple: a triangular mesh that covers the entire surface is created from 2D dataplots. Each vertex of the triangle has a depth assigned to it. The volume of the overall mesh is then calculated by summing the volume of each triangular prism as defined by the mesh. Note that in this particular approach, no interpolation is being done. Instead, a triangulated polygonal surface mesh is constructed from original depth data and the modeling.

### Hydrologic model

Rainfall was estimated with measured salinities before and after rain events using the equation [23]:
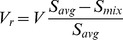
(2)adapted from a mass balance estuarine salt budget. It assumes the conservative mixing properties of salt between two water masses. 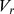 represents rainfall volume; 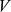, lake volume; 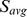, mean lake salinity prior to rain event; 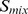, mean lake salinity of rain-mixed layer following rain event. 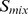 was calculated from post-rainfall salinity depth profiles. Each area bound by salinity vs. depth curves was estimated using the trapezoidal rule for definite integral approximation. The average salinity of lake water mixed with rainwater, 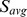, was obtained by solving for the salinity value that would bisecting the total area under the curve. Dividing calculated rain volume by lake surface area yielded estimates of linear rainfall.

### Tidal estimation and lake turnover time

For tidal estimation and lake turnover time, a simple tidal range estimation method was applied to lake-level readings collected prior to significant rainfall events. The minimum and consecutive maximum values were taken as the low and high for the tidal cycle, the difference being the total range. Time elapsed between the two readings was taken to be the duration of the flood tide, approximately half the tidal period. The tidal prism model of a well-mixed estuary was used to calculate lake turnover time. It follows the equation,
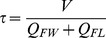
(3)
[7] where turnover 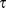 is equal to volume 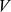, divided by the sum of seawater inflow on the flood tide, 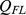, and freshwater inflow (P-E) during the flood tide, 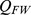. Values of 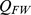 were derived from average literature values of regional precipitation minus evaporation (P – E) between June and July (see Supp. Info S1). 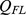 was derived from the tidal range estimate. A mass balance equation was used to solve for an unknown 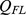 and make an additional turnover estimate using salinity measurements from within the lake and literature values for ocean salinity (see Supp. Info S1), and the assumed rainfall salinity of zero. In this equation,
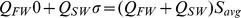
(4)where 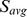 represents average lake salinity, 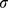 denotes salinity of seawater, and the seawater inflow 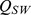, is equivalent to the floodwater inflow, 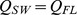.

### Lib sediment core radiocarbon dating

From the collected sediment cores at Lib Pond (see Table 4) we obtained organic material from which we got radiocarbon dates. (See Supp. Info S1 for individual measurements.) These dates tell us when the material, and implicitly the surrounding sediment layer, was deposited on the lake bottom, and therefore how old it is. The composition of characteristics of the sediment layer at that time reveal environmental change, as different types of sediment imply different environments. As stated in the introduction, from radiocarbon dating the cores we took give us a 3 ka climate history of Lib Pond, and by extension the region of Lib Island.

**Table 4:** Collected Lib Pond Sediment Cores.

Site Name	Date	Core	Location
MI-Lib 5	23-Jul-09	MI-Lib Ucore 5	8° 18.835′ N, 167° 22.873′ E
MI-Lib 6	23-Jul-09	MI-Lib Ucore 6	8° 18.852′ N, 167° 22.856′ E
MI-Lib 8	23-Jul-09	MI-Lib Ucore 8	8° 18.837′ N, 167° 22.870′ E
MI-Lib 6	23/24-Jul-09	MI-Lib Geocore 6	8° 18.852′ N, 167° 22.856′ E
MI-Lib 11	25-Jul-09	MI-Lib Geocore 11	*

*No GPS data available.

### Lib pond age model

A simplified age model assuming a linear interpolation between the radiocarbon dates of our sediment cores indicates some of the sediment collected in the Lib Pond cores is over a thousand years old. Samples were dated by the Xi'an AMS Center at the Insitute of Earth Environment, the Chinese Academy of Sciences (url: http://english.ieexa.cas.cn/rh/rd/200907/t20090719_23981.html), designated by ‘IEEE’. Samples dated by the Center for Accelerator Mass Spectrometry at the Lawrence Livermore National Laboratory (https://cams.llnl.gov/) are indicated by ‘CAMS’.

### Linear interpolation of geochemical profiles to estimate variation

We used a linear interpolation of geochemical profiles in Excel (using a pre-determined formula) to get values for a given geochemical property (temperature, salinity, pH, DO and DO%) of Lib Pond at a given lake location (Profile 1, 2, 3, 4, 5, 6) in half meter increments from the surface to 2.5 meters depth (0, 0.5, 1, 1.5, 2, 2.5 meters). We then averaged the values of the given geochemical property across locations at those depths and took the standard deviation of the average to create a horizontal error bar each in direction along the x-axis. These bars reflect the natural variation we observed of the geochemical properties of Lib Pond at given depths, as they varied across different geographical locations of the lake. They represent the maximum measurement error at a location, as each direction includes the full standard deviation of the averages for that geochemical property. (The error from the Eureka probe used to take geochemical measurements is negligible compared with the natural variation the Eureka probe recorded, so is not a useful error to display.)

## Supporting Information

Supporting Information S1 **Supporting Online Information for “Holocene Closure Inferred from a Physico-chemical and Bathymetric Survey of Lib Pond, Marshall Islands”.** This document contains supplementary material and details of the methods that were used in the paper.
(PDF)
