# Supplementary material for: Holocene Closure of Lib Pond, Marshall Islands
Source: PLoS One. 2014 Mar 17;9(3):e90939. doi: 10.1371/journal.pone.0090939 (PMC3956464; doi:10.1371/journal.pone.0090939)
Supplement: Supporting Information S1 — Supporting Online Information for “Holocene Closure Inferred from a Physico-chemical and Bathymetric Survey of Lib Pond, Marshall Islands”. This document contains supplementary material and details of the methods that were used in the paper. (PDF) [file pone.0090939.s001.pdf]

Supporting Online Information for "Holocene Closure Inferred from a Physico-chemical and Bathymetric Survey of Lib Pond, Marshall Islands"

Conor L. Myhrvold[1*], Fran Janny[1], Daniel Nelson[1], S. Nemiah Ladd[1], Alyssa Atwood[1], Julian P. Sachs[1]

[1] School of Oceanography, University of Washington, Seattle, Washington, United States of America

[*] Email: conor.myhrvold@gmail.com

This document contains supplementary material and details of the methods that were used in the paper.

Index

Page **1** Cover Page

Page **2** Index

Pages **3**-7 Lib Island Geography

Pages **8**-9 Lib Island in Context: Prior Sachs Studies

Page **10** Prior Mentions of Lib in the Scientific Literature

Page **11** Kwajalein Weather and Climate Data

Pages **12**-15 Kwajalein Tide Tables

Pages **16**-24 Lib Pond Bathymetry Data

Pages **25**-31 Field Data – Geochemistry

Page **32** P-E Box Model

Pages **33**-42 Sediment Core Data

Pages **43**-60 Hi-res Ucore Photograph Sequence

Pages **61**-75 Sediment Core Side-by-Side Photograph Sequence

Pages **76**-87 Lib Pond Vegetation Survey

Pages **88**-89 Conventional Bathymetry Background

Pages **90**-101 Boundary Slope Fitting Methodology

Pages **102**-108 Volume Calculation Discussion

Pages **108**-117 Error Analysis of Volume Estimation

Pages **118**-119 Water Volume Estimation

Pages **120**-121 References for Supporting Information

How to use index: Click on bold number of the section you want to go to (the beginning page).

Lib Island Geography

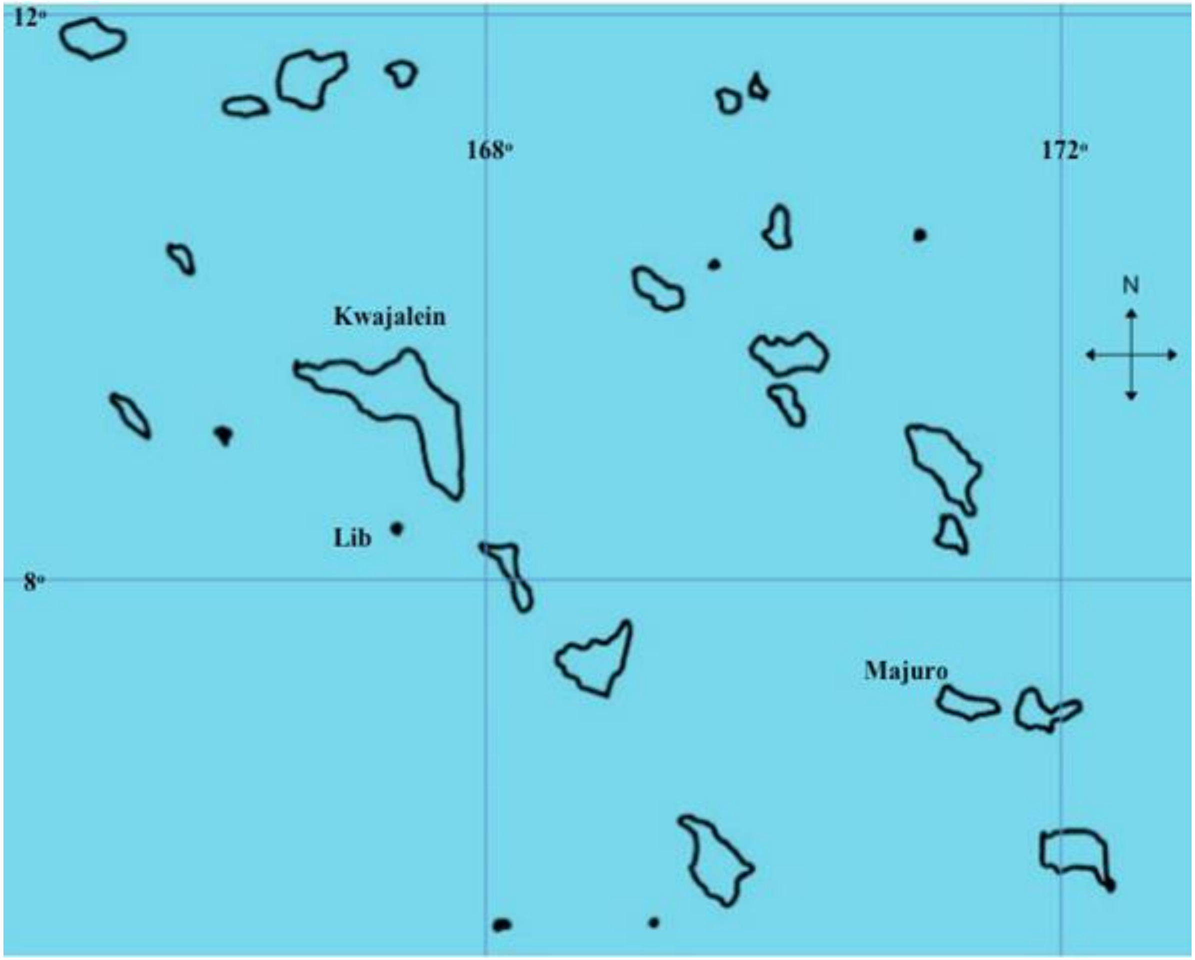

Figure S1. Modified map of the Republic of the Marshall Islands, specifying the positions of Lib Island, as well as Kwajalein and Majuro Atoll (the location of the capital city, Majuro). The Marshall Islands span from Ebon Atoll in the South to Bokak Atoll in the North, Ujelang Atoll in the West to Mili Atoll in the East.

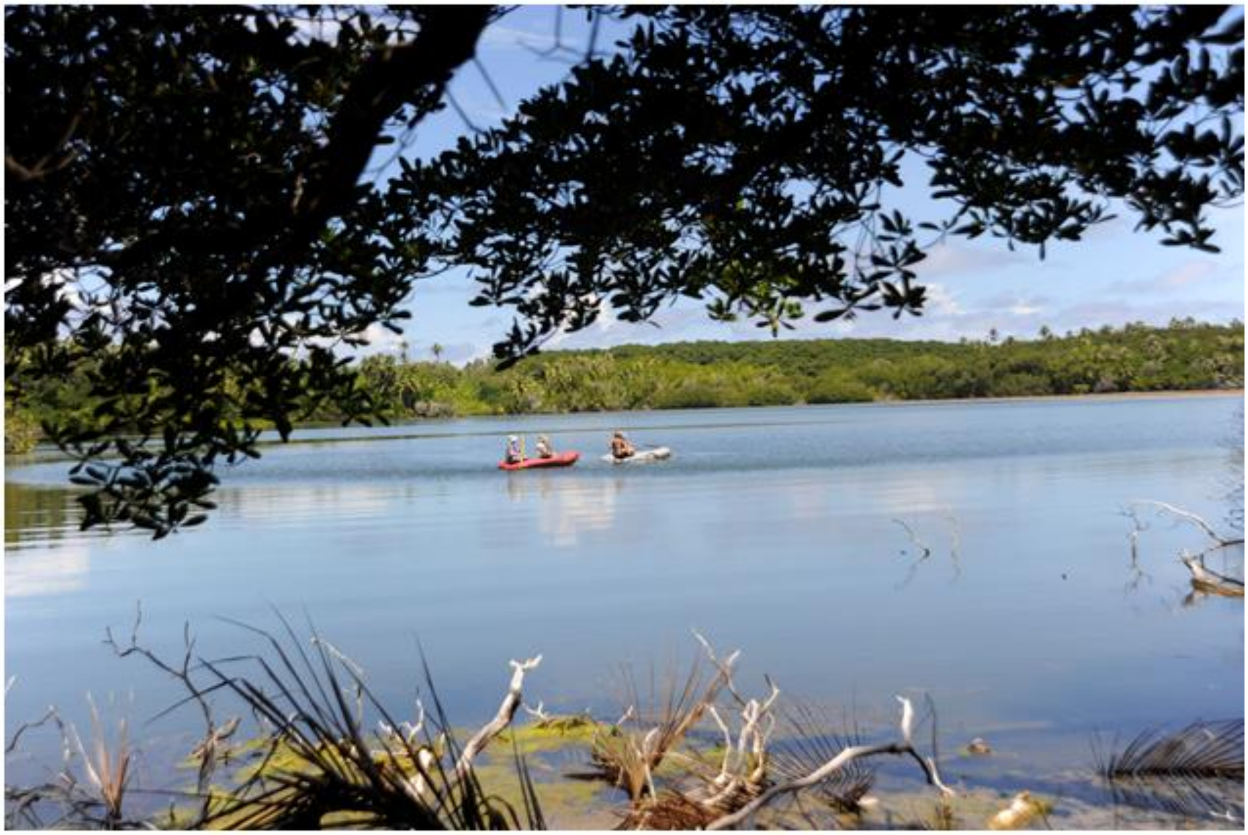

Figure S2. Inflatable row boats, used for the pond survey and coring, are shown with the lake shoreline in the foreground. The shoreline was highly vegetated and not easily accessible on foot. The view is looking toward the SE, from near the north end of Lib Pond.

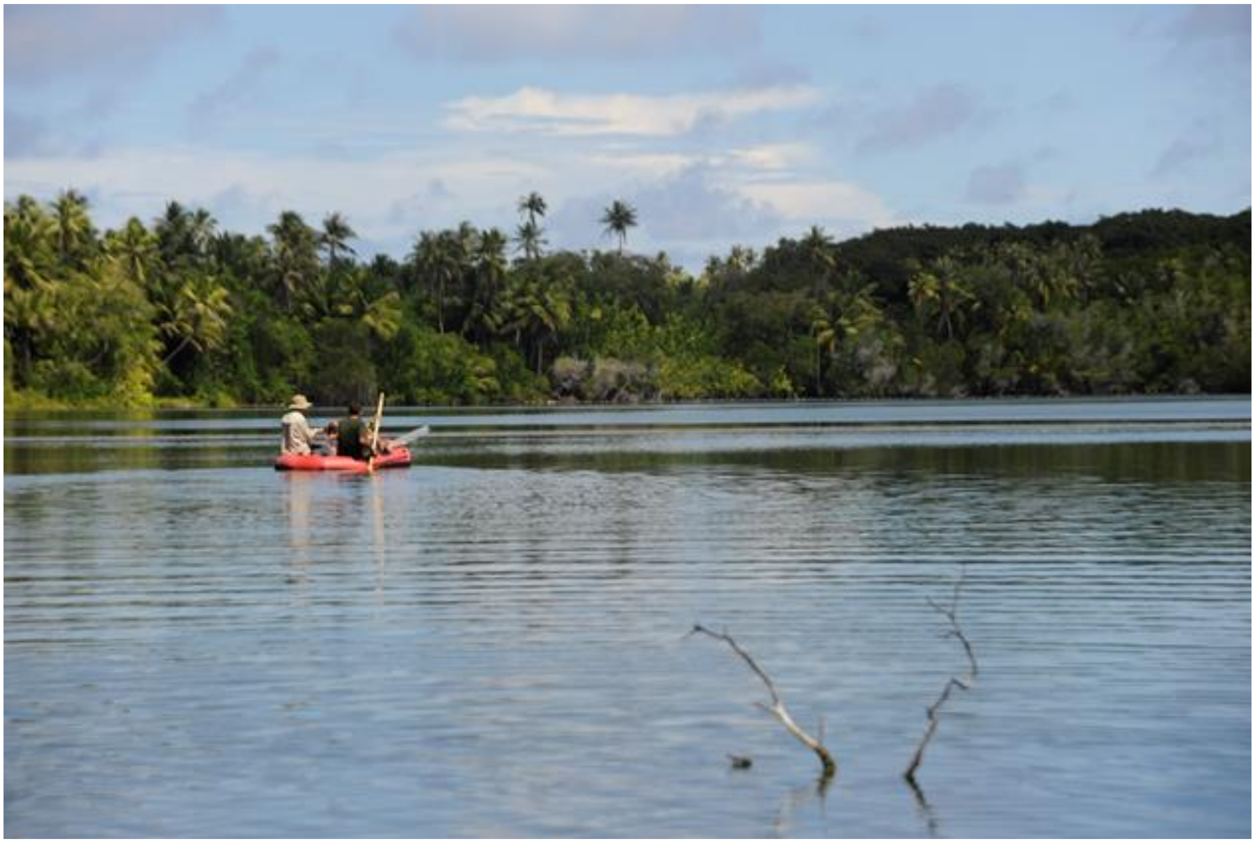

Figure S3. Lib Pond’s shallow depth is shown in the foreground by the branch, submerged but not in deep enough water to obscure it. The view is looking toward the SE, from near the north end of Lib Pond.

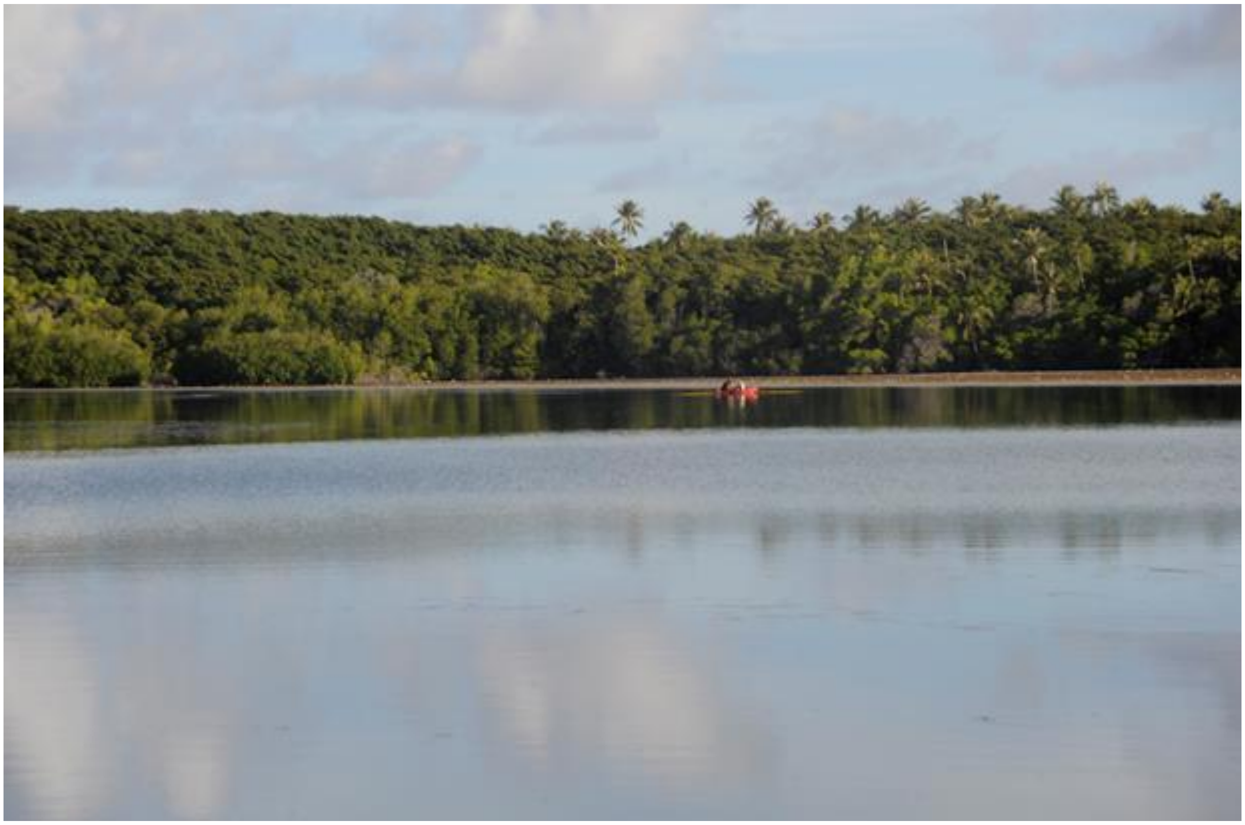

Figure S4. The south end of Lib Pond, as seen from the north shoreline (slightly west). In the right hand side of the pond, note the red-tan area which is the demarcation of the shallow floodplain assigned a uniform depth of 0.2 m in the bathymetric model. The view is looking south.

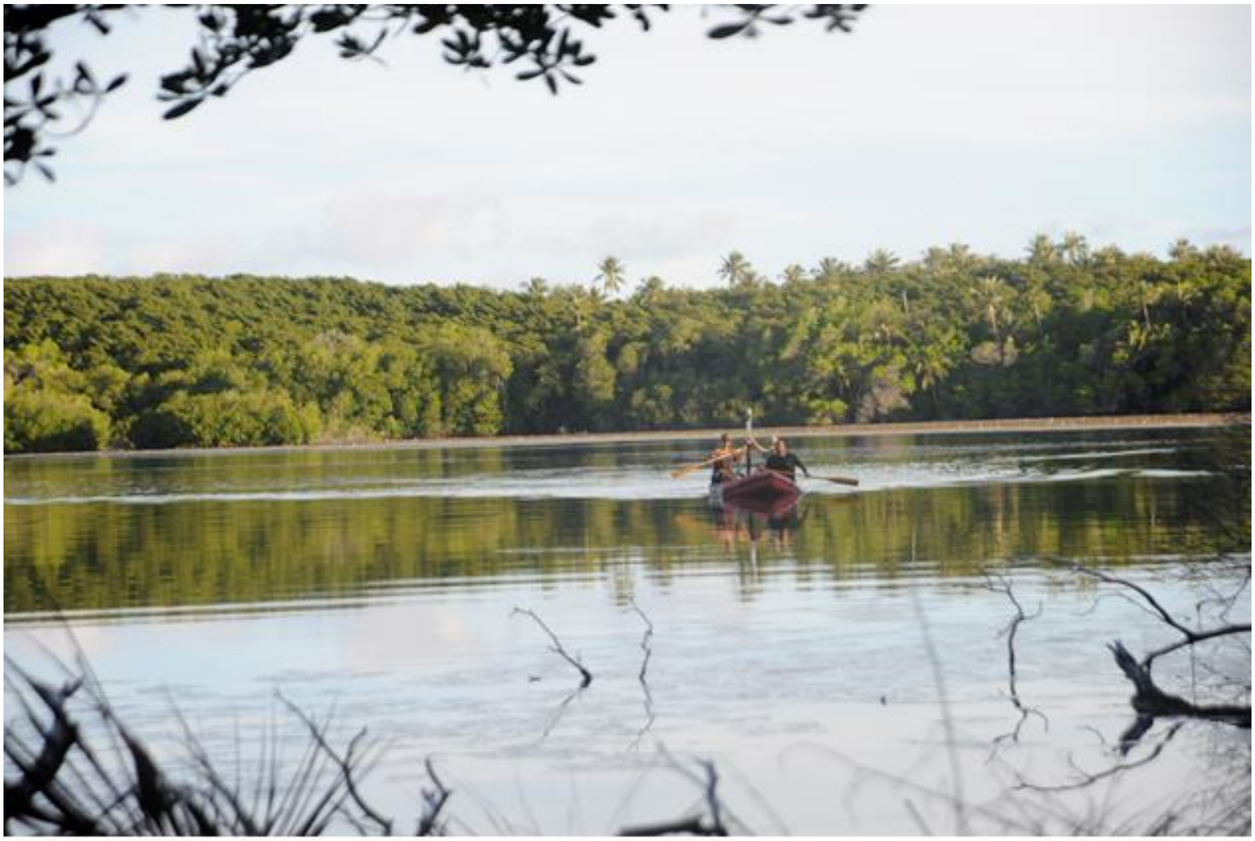

Figure S5. Returning with a Ucore, from the pond center where most of the cores were taken. Again, in the background note the long stretch of shallow floodplain. The out of focus vegetation toward the edges of the foreground is the same location as the previous photograph with vegetation in the foreground (**Figure S2**).

Lib Island in Context: Prior Sachs Studies

Field studies in the tropical Pacific islands carried out over the past decade by the Sachs Lab reveal regional rainfall patterns associated with ITCZ movement during the last 2 ka (2,000 years). These tropical paleoclimate records were constructed using measurements of the hydrogen isotopic composition ($^2H/^1H$, abbreviated in this section as δD) of lipid biomarkers in rapidly accumulating lake sediments.

Sediment cores from a freshwater lake on Washington Island (4°43'N) were collected and underwent δD analysis. δD values indicated that that Washington island was arid from 1450-1630 A.D., possibly until the late 18th century, coinciding with the LIA. δD analysis of dinosterol from sediments in Spooky Lake of Palau (7°09' N, 134°22' E) suggested that island was also arid during the LIA. δD studies were also conducted in El Junco lake on San Cristóbal Island in the Galápagos (0°54' S, 89°29' W), currently south of the ITCZ and arid. δD values designate the inverse climatic shift in the Galapagos, with wet conditions during the LIA. These observations together led to the conclusion of a southern displacement of the ITCZ by as much as 500 km during the LIA, between ~ 1430 and 1850 A.D. [1].

δD values of lipid biomarkers of phytoplankton and cyanobacteria can generate proxies for local paleohydrology. During photosynthesis, H_2O is converted to O_2 and H^+. This H^+ is incorporated into all biomolecules. Generally, δD values in lipids strongly correlate to ambient water δD values, with an offset associated with the biosynthetic pathway [2]. Under the environmental conditions observed in freshwater lakes in the equatorial Pacific, water δD values essentially produce a signal for the magnitude of precipitation minus evaporation (P-E). This is because the reservoir evaporative water loss exhibits fractionation favoring the lighter hydrogen

isotope over the heavier isotope, so high rates of evaporation result in residual water masses enriched with deuterium (higher δD values) [3].

The lake algae records the P-E signal as it builds lipids. When an alga dies it falls to the water body bottom incorporates into the sediment layer. Thus, over time a record of this P-E signal persists down the depth of sediment, and coupled with sediment chronologies produces a rainfall pattern history, provided that the sediment is undisturbed, thus maintaining the sedimentary law of superposition.

However, locally specific environmental factors – system inputs and outputs, salinity, and other growth rates – complicate a model for interpreting lipid δD measurements. For example, salinity can produce an additive signal in measured δD values based on the algal response of decreased D/H fractionation with extreme salinities [4]. So it was first necessary to establish the modern physico-chemical characteristics and variability on Christmas Island, which vary dramatically on small spatial scales, in conjunction with pond isolation and degree of communication with seawater [5]. Under the conditions observed in the meromictic Spooky Lake (Palau), intrusion of the saline sub-layer, varying in part as a function of P-E, adds an additional δD signature to the reservoir [6].

So because conditions specific to an aquatic environment affect the interpretation of lipid hydrogen isotope ratios – and by extension indications of paleoclimate – constraints on the current variability of lake hydrology need to be accurately defined. Sediment cores collected from Lib Island provide constraints for the general Marshall Islands region, and water bodies elsewhere with similar latitudes or geochemical properties. Field studies had never been conducted at Lib Island [7] and thus no hydrologic data existed for Lib Pond.

Prior Mentions of Lib in the Scientific Literature

Lib Island was briefly summarized by renowned 20th century tropics researcher Francis Raymond Fosberg in June 1988 [7]:

Lib Island

This island or table reef lies at 08° 19' N, 167° 25' E, south of Kwajalein. It has a large fresh-water pond in the eastern half, apparently containing some mangroves (Bruguiera ?). No scientific information is available, but I examined the island briefly from the air in 1960. It is inhabited and partly planted to coconuts. However, there is considerable native forest remaining on the north side

85

and around the pond. Tournefortia, Scaevola, Calophyllum, Pandanus, Hibiscus tiliaceus, Bruguiera, Artocarpus and Cocos, could be identified with some confidence from the air. This island would well repay a visit and careful study. I know of no collections of plants, birds, or other scientific specimens from Lib.

Figure S6.

Apart from that, we know of no description of Lib Island in the literature despite an extensive search. With this geochemical study, we have aimed to undertake the study Fosberg suggested.

Kwajalein Weather and Climate Data

Kwajalein Atoll Annual Rainfall vs. SOI

The total annual rainfall over Kwajalein Atoll (1951-2007, dotted red line) superimposed on the Southern Oscillation Index (1951-2009, solid black line). Given that the precipitation appears to closely match SOI oscillations Kwajalein, and therefore Lib, has local characteristics influenced by larger, regional trends in the equatorial Pacific Ocean.

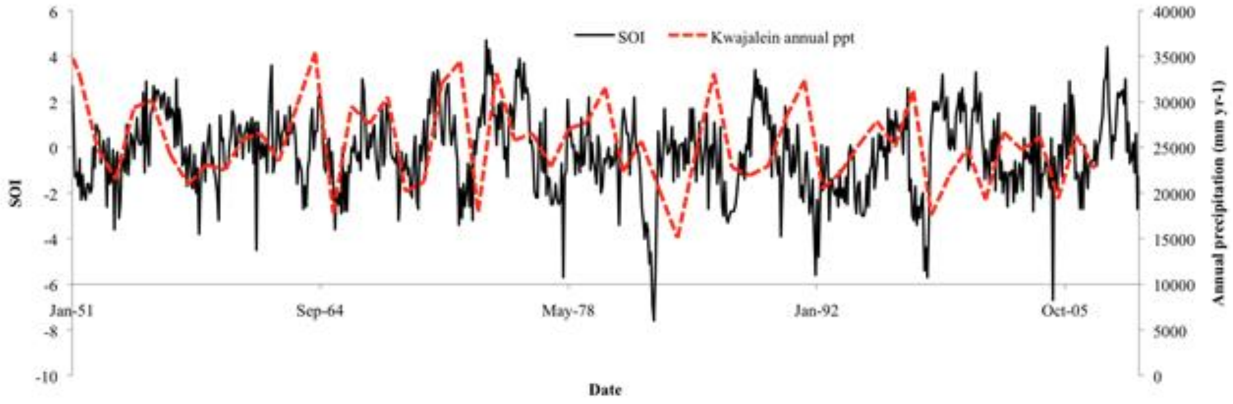

Figure S7. Sources: [8] and the Southern Oscillation Index, 2009.

Kwajalein Tide Tables

Kwajalein Atoll Tide Tables, July 2009

Date	Time	Tide	Height (feet)
July 21	03:27 AM	High	4.7
	10:01 AM	Low	-0.6
	04:00 PM	High	3.5
	09:49 PM	Low	-0.4
July 22	04:13 AM	High	5.0
	10:43 AM	Low	-0.9
	04:43 PM	High	3.8
	10:34 PM	Low	-0.6
July 23	04:55 AM	High	5.2
	11:22 AM	Low	-1.0
	05:23 PM	High	4.0
	11:16 PM	Low	-0.7
July 24	05:36 AM	High	5.2
	12:00 PM	Low	-1.0
	06:02 PM	High	4.1
	11:57 PM	Low	-0.6
July 25	06:14 AM	High	4.9
	12:36 PM	Low	-0.8
	06:41 PM	High	4.0
July 26	12:38 AM	Low	-0.4

Table S1.

Source #1: Here we show Kwajalein tide tables during the Lib Pond study duration (July 21-25), used as a reference for lake levels we measured at Lib Pond, which inferred tidal change.

Note from data source, Kwajalein RTS-WX Meteorological Station website: *“All times are listed in Local Standard Time. All heights are in feet referenced to Mean Lower Low Water (MLLW). Data courtesy of NOAA Tides and Currents and ATSC.”*

NOAA Tide Predictions

Kwajalein Atoll (Namur Island),,2009

Times and Heights of High and Low Waters

StationId:TPT2697
Source:NOAA/NOS/CO-OPS
Station Type:Subordinate
Time Zone:LST/LDT
Datum:mean lower low water (MLLW) which is the chart datum of soundings

July

Day	Time (h m)	Height (ft)	Height (cm)
1 W	06:13 AM	1.2	37
	12:03 PM	2.5	76
	06:17 PM	0.8	24
2 Th	01:13 AM	3.1	94
	07:50 AM	1.1	34
	01:34 PM	2.3	70
	07:28 PM	0.9	27
3 F	02:21 AM	3.3	101
	09:03 AM	0.8	24
	02:50 PM	2.4	73
	08:31 PM	0.7	21
4 Sa	03:14 AM	3.6	110
	09:53 AM	0.5	15
	03:44 PM	2.5	76
	09:22 PM	0.6	18
5 Su	03:57 AM	3.8	116
	10:31 AM	0.2	6
	04:26 PM	2.7	82
	10:05 PM	0.3	9
6 M	04:34 AM	4.0	122
	11:05 AM	0.0	0
	05:01 PM	2.9	88
	10:42 PM	0.1	3
7 Tu	05:09 AM	4.2	128
	11:35 AM	-0.2	-6
	05:34 PM	3.1	94
	11:17 PM	0.0	0
8 W	05:41 AM	4.3	131
	12:05 PM	-0.3	-9
	06:04 PM	3.3	101
	11:49 PM	-0.1	-3
9 Th	06:12 AM	4.4	134
	12:34 PM	-0.4	-12
	06:34 PM	3.4	104
10 F	12:20 AM	-0.1	-3
	06:42 AM	4.4	134
	01:03 PM	-0.3	-9
	07:04 PM	3.4	104
11 Sa	12:51 AM	-0.1	-3
	07:11 AM	4.3	131
	01:31 PM	-0.2	-6
	07:34 PM	3.4	104
12 Su	01:22 AM	0.1	3
	07:40 AM	4.1	125
	01:59 PM	-0.1	-3
	08:06 PM	3.4	104
13 M	01:55 AM	0.3	9
	08:10 AM	3.9	119
	02:30 PM	0.0	0
	08:41 PM	3.3	101
14 Tu	02:33 AM	0.5	15
	08:45 AM	3.6	110
	03:04 PM	0.2	6
	09:23 PM	3.2	98
15 W	03:21 AM	0.8	24
	09:27 AM	3.2	98
	03:46 PM	0.5	15
	10:19 PM	3.1	94
16 Th	04:29 AM	1.0	30
	10:27 AM	2.7	82
	04:45 PM	0.7	21
	11:37 PM	3.1	94
17 F	06:11 AM	1.1	34
	12:02 PM	2.4	73
	06:09 PM	0.8	24
18 Sa	01:11 AM	3.3	101
	08:00 AM	0.8	24
	01:54 PM	2.4	73
	07:42 PM	0.7	21
19 Su	02:31 AM	3.7	113
	09:16 AM	0.3	9
	03:15 PM	2.7	82
	08:56 PM	0.4	12
20 M	03:33 AM	4.2	128
	10:10 AM	-0.2	-6
	04:11 PM	3.1	94
	09:54 PM	0.0	0
21 Tu	04:25 AM	4.7	143
	10:56 AM	-0.6	-18
	04:58 PM	3.5	107
	10:43 PM	-0.4	-12
22 W	05:10 AM	5.0	152
	11:38 AM	-0.9	-27
	05:41 PM	3.8	116
	11:28 PM	-0.6	-18
23 Th	05:53 AM	5.2	158
	12:17 PM	-1.0	-30
	06:21 PM	4.0	122
24 F	12:11 AM	-0.7	-21
	06:34 AM	5.2	158
	12:55 PM	-1.0	-30
	07:00 PM	4.1	125
25 Sa	12:52 AM	-0.6	-18
	07:12 AM	4.9	149
	01:31 PM	-0.8	-24
	07:38 PM	4.0	122
26 Su	01:33 AM	-0.4	-12
	07:49 AM	4.5	137
	02:06 PM	-0.5	-15
	08:17 PM	3.9	119
27 M	02:14 AM	0.0	0
	08:25 AM	4.0	122
	02:40 PM	-0.1	-3
	08:57 PM	3.6	110
28 Tu	02:58 AM	0.5	15
	09:02 AM	3.4	104
	03:13 PM	0.3	9
	09:41 PM	3.3	101
29 W	03:50 AM	0.9	27
	09:41 AM	2.7	82
	03:51 PM	0.7	21
	10:39 PM	3.0	91
30 Th	05:08 AM	1.3	40
	10:40 AM	2.2	67
	04:46 PM	1.1	34
31 F	12:09 AM	2.8	85
	07:29 AM	1.3	40
	12:58 PM	1.9	58
	06:33 PM	1.2	37

Table S2.

Source #2: Reformatted NOAA tide table estimates for July 2009, Kwajalein Atoll. July 21 – 25 highlighted in blue, which were compared against recorded Lib Pond tide measurements. These NOAA estimates differ slightly from the meteorology station estimates in time, but not in tide height (with offsets of under an hour in each instance).

Kwajalein Atoll Tide Tables, July 2009

Date	Time	Tide	Height (feet)
July 21	03:29 AM	High	5.87
	10:04 AM	Low	0.62
	04:01 PM	High	4.60
	09:48 PM	Low	0.82
July 22	04:13 AM	High	6.20
	10:44 AM	Low	0.32
	04:42 PM	High	4.90
	10:32 PM	Low	0.58
July 23	04:54 AM	High	6.35
	11:22 AM	Low	0.19
	05:22 PM	High	5.10
	11:14 PM	Low	0.48
July 24	05:33 AM	High	6.29
	11:58 AM	Low	0.21
	06:00 PM	High	5.18
	11:55 PM	Low	0.55
July 25	06:11 AM	High	6.04
	12:33 PM	Low	0.39
	06:47 PM	High	5.13
July 26	12:34 AM	Low	0.77

Table S3. Source #3.

This third source differs in tide height, and not in time, compared to Source #1 (**Table S1**).

Source:

http://tides.mobilegeographics.com/calendar/month/3203.html?y=2009&m=7&d=24

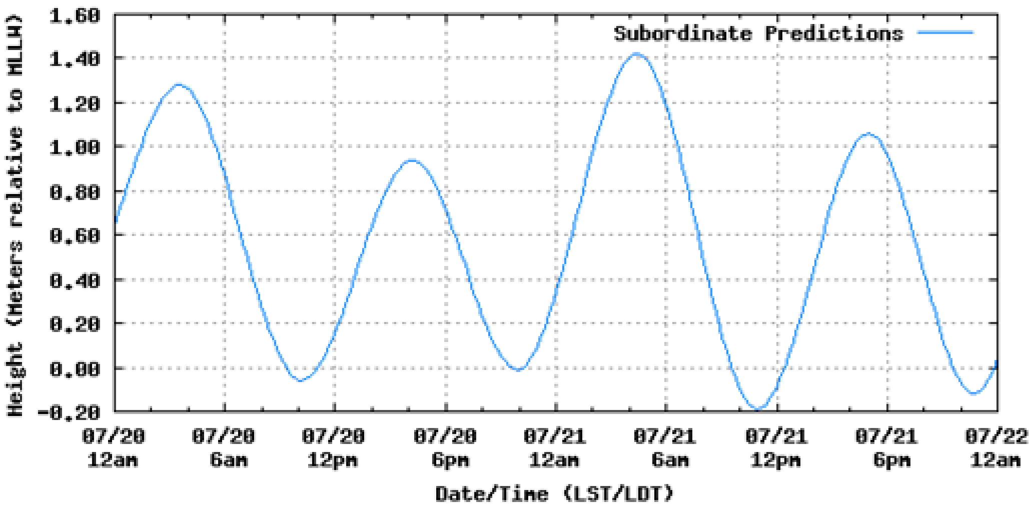

Figure S8. Tide predictions on Kwajalein Atoll for 7/20/09 to 7/22/09, the start of Lib Pond tide measurements, from NOAA estimates (figure is reprinted).

Three sources of external tide data for Kwajalein each gave different values, so all variations are displayed. Source #1 and Source #2 had similar tide magnitudes, but different times. Source #1 and Source #3 had similar times, but different magnitudes. Source #2 and Source #3 had different times and different magnitudes compared to each other.

Lib Pond Bathymetry Data

The GPS coordinates were imported from the Garmin GPS units to GoogleEarth. However, they needed to be offset given the GPS error, which placed several pond depth coordinates beyond the body of Lib Pond, and past the shoreline on land. Since the original pond orientation top was not true north, the lake satellite image from Google Earth was reoriented and rotated in Mathematica using the Lambert Azmuthal projection. There was only one good fit within the pond dimensions, given that we traversed the pond edge and hugged the shore at a section on the SE and NE shore. The outline of Lib Pond was drawn in Mathematica and exported back into GoogleEarth, along with the original depth pairings. For images showing this, please contact the corresponding author.

Satellite Image Alignment

The satellite image has a crosshair reticle at a specified point in the lake (Latitude, Longitude). It was aligned such that the crosshair was centered over the coordinates (0,0) in a Lambert Azmuthal projection in Mathematica, which has a built in function for displaying that particular projection. Thus, georeferencing error is negligible. Below, the red points indicate points at which the depth was taken, and the pink line indicates the extent of the floodplain from the lower left quadrant and out. The blue line are the perimeters of Lib Pond.

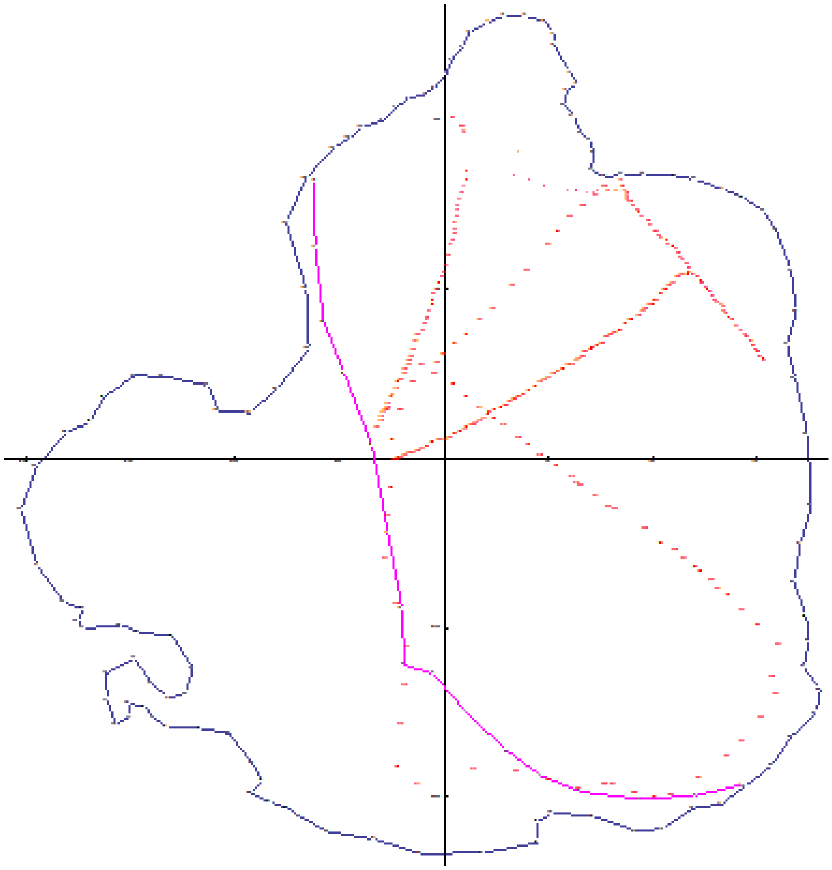

Figure S9.

Lib Pond GPS Coordinates and Depth Pairings

This original dataset was used to construct the bathymetric map of Lib Pond (positions are before the offset correction from GPS error.) The transects were composed of 263 GPS coordinate and depth pairs. Data points were taken on July 21st and 23rd at comparable tidal periods.

Table S4:

**Time**	**GPS Coordinates**	**Depth (meters)**
21-JUL-09	N8.31524 E167.38141	2.9
21-JUL-09	N8.31511 E167.38140	2.5
21-JUL-09	N8.31509 E167.38146	2.7
21-JUL-09	N8.31506 E167.38153	2.7
21-JUL-09	N8.31503 E167.38163	2.7
21-JUL-09	N8.31503 E167.38167	2.6
21-JUL-09	N8.31502 E167.38171	2.4
21-JUL-09	N8.31502 E167.38174	2
21-JUL-09	N8.31503 E167.38176	1.4
21-JUL-09	N8.31503 E167.38180	1.6
21-JUL-09	N8.31504 E167.38184	1.9
21-JUL-09	N8.31505 E167.38187	2.1
21-JUL-09	N8.31509 E167.38186	1.6
21-JUL-09	N8.31506 E167.38187	1.8
21-JUL-09	N8.31504 E167.38188	2
21-JUL-09	N8.31502 E167.38189	2.2
21-JUL-09	N8.31501 E167.38189	2.2
21-JUL-09	N8.31500 E167.38189	2.3
21-JUL-09	N8.31499 E167.38189	2.3
21-JUL-09	N8.31498 E167.38190	2.4
21-JUL-09	N8.31497 E167.38191	2.6
21-JUL-09	N8.31495 E167.38192	2.7
21-JUL-09	N8.31493 E167.38193	2.6
21-JUL-09	N8.31492 E167.38194	2.7
21-JUL-09	N8.31490 E167.38195	2.8
21-JUL-09	N8.31487 E167.38198	2.8
21-JUL-09	N8.31485 E167.38199	2.8
21-JUL-09	N8.31482 E167.38201	2.9
21-JUL-09	N8.31481 E167.38202	2.9

21-JUL-09	N8.31479 E167.38203	3
21-JUL-09	N8.31478 E167.38204	2.9
21-JUL-09	N8.31477 E167.38206	3
21-JUL-09	N8.31475 E167.38207	3
21-JUL-09	N8.31473 E167.38209	3
21-JUL-09	N8.31472 E167.38210	3
21-JUL-09	N8.31470 E167.38211	3.1
21-JUL-09	N8.31468 E167.38212	3
21-JUL-09	N8.31466 E167.38213	3.1
21-JUL-09	N8.31464 E167.38215	3
21-JUL-09	N8.31462 E167.38216	3
21-JUL-09	N8.31459 E167.38218	2.9
21-JUL-09	N8.31457 E167.38220	2.9
21-JUL-09	N8.31455 E167.38221	3.1
21-JUL-09	N8.31454 E167.38222	3.1
21-JUL-09	N8.31452 E167.38223	3
21-JUL-09	N8.31450 E167.38224	3.1
21-JUL-09	N8.31449 E167.38225	3.1
21-JUL-09	N8.31447 E167.38226	3
21-JUL-09	N8.31445 E167.38227	3.1
21-JUL-09	N8.31444 E167.38228	3.1
21-JUL-09	N8.31442 E167.38230	3.2
21-JUL-09	N8.31441 E167.38231	3.2
21-JUL-09	N8.31439 E167.38231	3.2
21-JUL-09	N8.31438 E167.38232	3.1
21-JUL-09	N8.31436 E167.38233	3.1
21-JUL-09	N8.31434 E167.38235	3.1
21-JUL-09	N8.31431 E167.38237	3
21-JUL-09	N8.31429 E167.38238	2.9
21-JUL-09	N8.31427 E167.38240	2.9
21-JUL-09	N8.31425 E167.38241	2.9
21-JUL-09	N8.31423 E167.38243	2.8
21-JUL-09	N8.31421 E167.38244	2.8
21-JUL-09	N8.31419 E167.38245	2.8
21-JUL-09	N8.31417 E167.38246	2.6
21-JUL-09	N8.31415 E167.38247	2.2
21-JUL-09	N8.31414 E167.38248	1.6
21-JUL-09	N8.31413 E167.38249	1.1
21-JUL-09	N8.31542 E167.38113	2.7
21-JUL-09	N8.31538 E167.38117	2.7

21-JUL-09	N8.31537 E167.38118	2.7
21-JUL-09	N8.31536 E167.38118	2.6
21-JUL-09	N8.31534 E167.38118	2.6
21-JUL-09	N8.31513 E167.38119	2.6
21-JUL-09	N8.31509 E167.38119	2.5
21-JUL-09	N8.31506 E167.38118	2.4
21-JUL-09	N8.31502 E167.38117	2.4
21-JUL-09	N8.31499 E167.38116	2.3
21-JUL-09	N8.31496 E167.38115	2.4
21-JUL-09	N8.31493 E167.38115	2.4
21-JUL-09	N8.31489 E167.38115	2.5
21-JUL-09	N8.31486 E167.38114	2.4
21-JUL-09	N8.31483 E167.38114	2.4
21-JUL-09	N8.31480 E167.38114	2.4
21-JUL-09	N8.31477 E167.38113	2.4
21-JUL-09	N8.31474 E167.38112	2.3
21-JUL-09	N8.31469 E167.38111	2.3
21-JUL-09	N8.31463 E167.38110	2.4
21-JUL-09	N8.31460 E167.38109	2.4
21-JUL-09	N8.31457 E167.38108	2.4
21-JUL-09	N8.31454 E167.38107	2.3
21-JUL-09	N8.31451 E167.38106	2.5
21-JUL-09	N8.31448 E167.38105	2.4
21-JUL-09	N8.31445 E167.38104	2.3
21-JUL-09	N8.31443 E167.38104	2.3
21-JUL-09	N8.31439 E167.38103	2.2
21-JUL-09	N8.31436 E167.38102	2.1
21-JUL-09	N8.31434 E167.38102	2.2
21-JUL-09	N8.31432 E167.38101	2.2
21-JUL-09	N8.31429 E167.38100	2.2
21-JUL-09	N8.31426 E167.38098	2.1
21-JUL-09	N8.31423 E167.38097	2
21-JUL-09	N8.31419 E167.38096	1.9
21-JUL-09	N8.31415 E167.38095	1.9
21-JUL-09	N8.31413 E167.38094	1.9
21-JUL-09	N8.31411 E167.38094	1.9
21-JUL-09	N8.31408 E167.38092	1.8
21-JUL-09	N8.31406 E167.38092	1.8
21-JUL-09	N8.31403 E167.38090	1.7
21-JUL-09	N8.31401 E167.38089	1.7

21-JUL-09	N8.31398 E167.38088	1.7
21-JUL-09	N8.31395 E167.38087	1.6
21-JUL-09	N8.31393 E167.38086	1.6
21-JUL-09	N8.31390 E167.38085	1.6
21-JUL-09	N8.31388 E167.38085	1.5
21-JUL-09	N8.31387 E167.38085	1.3
21-JUL-09	N8.31386 E167.38084	1.2
21-JUL-09	N8.31385 E167.38083	1
21-JUL-09	N8.31383 E167.38082	0.9
21-JUL-09	N8.31381 E167.38082	0.8
21-JUL-09	N8.31377 E167.38080	0.8
21-JUL-09	N8.31460 E167.38216	3
21-JUL-09	N8.31459 E167.38215	3
21-JUL-09	N8.31458 E167.38213	3
21-JUL-09	N8.31457 E167.38211	3
21-JUL-09	N8.31454 E167.38209	3
21-JUL-09	N8.31453 E167.38207	3.1
21-JUL-09	N8.31451 E167.38206	3.1
21-JUL-09	N8.31450 E167.38205	3.1
21-JUL-09	N8.31448 E167.38204	3.1
21-JUL-09	N8.31445 E167.38202	3.1
21-JUL-09	N8.31445 E167.38201	3.2
21-JUL-09	N8.31443 E167.38200	3.1
21-JUL-09	N8.31442 E167.38199	3.1
21-JUL-09	N8.31440 E167.38197	3
21-JUL-09	N8.31439 E167.38196	3
21-JUL-09	N8.31437 E167.38194	3
21-JUL-09	N8.31435 E167.38191	3
21-JUL-09	N8.31434 E167.38190	2.9
21-JUL-09	N8.31432 E167.38188	2.8
21-JUL-09	N8.31429 E167.38186	2.9
21-JUL-09	N8.31428 E167.38184	2.8
21-JUL-09	N8.31427 E167.38182	2.8
21-JUL-09	N8.31425 E167.38180	2.9
21-JUL-09	N8.31422 E167.38178	3
21-JUL-09	N8.31421 E167.38176	2.9
21-JUL-09	N8.31420 E167.38174	3
21-JUL-09	N8.31418 E167.38173	2.9
21-JUL-09	N8.31417 E167.38171	2.7
21-JUL-09	N8.31415 E167.38170	2.6

21-JUL-09	N8.31414 E167.38168	2.5
21-JUL-09	N8.31413 E167.38167	2.5
21-JUL-09	N8.31411 E167.38165	2.4
21-JUL-09	N8.31410 E167.38163	2.4
21-JUL-09	N8.31408 E167.38161	2.4
21-JUL-09	N8.31407 E167.38159	2.3
21-JUL-09	N8.31406 E167.38157	2.3
21-JUL-09	N8.31404 E167.38155	2.2
21-JUL-09	N8.31402 E167.38153	2.2
21-JUL-09	N8.31401 E167.38151	2.1
21-JUL-09	N8.31399 E167.38149	2.1
21-JUL-09	N8.31397 E167.38146	2
21-JUL-09	N8.31395 E167.38144	2
21-JUL-09	N8.31393 E167.38142	2
21-JUL-09	N8.31392 E167.38140	2
21-JUL-09	N8.31391 E167.38138	1.9
21-JUL-09	N8.31389 E167.38136	1.9
21-JUL-09	N8.31388 E167.38134	1.9
21-JUL-09	N8.31386 E167.38131	2
21-JUL-09	N8.31385 E167.38129	1.8
21-JUL-09	N8.31384 E167.38128	1.9
21-JUL-09	N8.31382 E167.38127	1.9
21-JUL-09	N8.31381 E167.38125	1.8
21-JUL-09	N8.31380 E167.38123	1.8
21-JUL-09	N8.31379 E167.38122	1.8
21-JUL-09	N8.31378 E167.38121	1.7
21-JUL-09	N8.31377 E167.38119	1.7
21-JUL-09	N8.31376 E167.38118	1.6
21-JUL-09	N8.31375 E167.38116	1.6
21-JUL-09	N8.31374 E167.38114	1.6
21-JUL-09	N8.31372 E167.38112	1.6
21-JUL-09	N8.31371 E167.38110	1.6
21-JUL-09	N8.31370 E167.38107	1.6
21-JUL-09	N8.31369 E167.38104	1.6
21-JUL-09	N8.31367 E167.38102	1.6
21-JUL-09	N8.31366 E167.38100	1.7
21-JUL-09	N8.31365 E167.38098	1.7
21-JUL-09	N8.31364 E167.38096	1.6
21-JUL-09	N8.31364 E167.38094	1.5
21-JUL-09	N8.31362 E167.38092	1.3

21-JUL-09	N8.31361 E167.38090	1.1
21-JUL-09	N8.31361 E167.38089	0.9
21-JUL-09	N8.31360 E167.38087	0.9
23-JUL-09	N8.31412 E167.38106	2.1
23-JUL-09	N8.31416 E167.38109	2.2
23-JUL-09	N8.31422 E167.38114	2.3
23-JUL-09	N8.31427 E167.38119	2.4
23-JUL-09	N8.31434 E167.38125	2.5
23-JUL-09	N8.31440 E167.38130	2.7
23-JUL-09	N8.31453 E167.38139	2.8
23-JUL-09	N8.31461 E167.38145	2.8
23-JUL-09	N8.31475 E167.38155	3
23-JUL-09	N8.31482 E167.38159	3.1
23-JUL-09	N8.31489 E167.38164	2.9
23-JUL-09	N8.31495 E167.38170	3
23-JUL-09	N8.31501 E167.38175	2
23-JUL-09	N8.31505 E167.38179	0.8
23-JUL-09	N8.31410 E167.38100	2.1
23-JUL-09	N8.31406 E167.38105	2.2
23-JUL-09	N8.31400 E167.38113	2.2
23-JUL-09	N8.31393 E167.38119	2.1
23-JUL-09	N8.31388 E167.38125	2.1
23-JUL-09	N8.31382 E167.38131	2.1
23-JUL-09	N8.31378 E167.38135	2.1
23-JUL-09	N8.31372 E167.38142	2.1
23-JUL-09	N8.31368 E167.38146	2.2
23-JUL-09	N8.31363 E167.38151	2.2
23-JUL-09	N8.31357 E167.38158	2.3
23-JUL-09	N8.31351 E167.38165	2.3
23-JUL-09	N8.31348 E167.38167	2.4
23-JUL-09	N8.31346 E167.38169	2.5
23-JUL-09	N8.31341 E167.38175	2.6
23-JUL-09	N8.31335 E167.38181	2.7
23-JUL-09	N8.31334 E167.38184	2.8
23-JUL-09	N8.31324 E167.38197	3
23-JUL-09	N8.31316 E167.38204	3.1
23-JUL-09	N8.31312 E167.38210	3.1
23-JUL-09	N8.31308 E167.38214	3.2
23-JUL-09	N8.31303 E167.38219	3.2
23-JUL-09	N8.31301 E167.38221	3.1

23-JUL-09	N8.31296 E167.38226	3.2
23-JUL-09	N8.31292 E167.38229	3.1
23-JUL-09	N8.31288 E167.38233	2.8
23-JUL-09	N8.31280 E167.38239	2.2
23-JUL-09	N8.31272 E167.38246	0.9
23-JUL-09	N8.31262 E167.38255	2.2
23-JUL-09	N8.31245 E167.38252	2.8
23-JUL-09	N8.31236 E167.38254	2
23-JUL-09	N8.31223 E167.38247	2
23-JUL-09	N8.31210 E167.38239	0.8
23-JUL-09	N8.31202 E167.38232	0.3
23-JUL-09	N8.31191 E167.38220	0.3
23-JUL-09	N8.31182 E167.38208	0.1
23-JUL-09	N8.31181 E167.38201	0.1
23-JUL-09	N8.31183 E167.38192	1.2
23-JUL-09	N8.31188 E167.38183	1.6
23-JUL-09	N8.31187 E167.38179	0.1
23-JUL-09	N8.31185 E167.38168	0.1
23-JUL-09	N8.31190 E167.38155	0.2
23-JUL-09	N8.31194 E167.38141	0.1
23-JUL-09	N8.31195 E167.38122	0.1
23-JUL-09	N8.31188 E167.38098	0.1
23-JUL-09	N8.31197 E167.38089	0.1
23-JUL-09	N8.31220 E167.38090	0.2
23-JUL-09	N8.31240 E167.38092	0.2
23-JUL-09	N8.31261 E167.38093	0.2
23-JUL-09	N8.31283 E167.38088	0.3
23-JUL-09	N8.31308 E167.38084	0.3
23-JUL-09	N8.31331 E167.38085	0.6
23-JUL-09	N8.31345 E167.38086	0.8
23-JUL-09	N8.31360 E167.38088	1.2
23-JUL-09	N8.31371 E167.38087	1.7
23-JUL-09	N8.31387 E167.38090	1.9
23-JUL-09	N8.31401 E167.38096	2.2

GPS Settings
Grid: Lat/Lon hddd.ddddd⁻ **Datum:** WGS 84

Field Data – Geochemistry

Table S5. Field Equipment Used

Measurement	Device and Model #	Other Notes
Pond depths	NorCross infrared depth sounder	Paired with GPS waypoints
GPS coordinates	Garmin 76CSx and Garmin Etrex GPS trackers	Paired with depth waypoints
Pond profile measurements (pH, salinity, DO etc.)	Eureka Environmental Manta2 Water Quality Multiprobe	Data was logged directly with the Eureka Amphibian field display program on a battery-powered PDA.
Tide recording	Measured the vertical distance between pond water surface and a marker positioned on a fixed tree branch 2 m from shore.	Measured at GPS coordinates 08° 18.818’ N, 167° 22.455’ E

Table S6. Lib Pond Tide Data, July 21-25 2009

Date	Time	Tide (cm)	Notes
July 21	11:16 am	0	Tide gauge set.
July 21	2:19 pm	1.1-	
July 21	4:07 pm	0.5-	
July 22	11:24 pm	2+	
July 22	1:55 pm	1.6+	
July 22	6:15 pm	1.9+	
July 22	6:44 pm	1.9+	
July 23	10:11 am	6.7+	During rain event.
July 23	7:29 pm	6.9+	During rain event.
July 24	11:25 pm	25.8+	After long rain event (previous evening and overnight).
July 24	7:30 pm	25+	
July 25	9:36 am	25.5+	New tape mark set 25.5 cm above previous one; subsequent measurements are distance above new mark + 25.5 cm.
July 25	4:05 pm	28+	

Location of some of Sediment Cores and Geochemistry Profiles

For a zoomed-in satellite image of Lib pond, labeling core sampling and water profiling sites with available GPS data, please contact the corresponding author (image is a GoogleEarth screenshot).

Table S7. Raw Data of Pond Geochemistry Profiles

Profile	Date	Time	Depth (m)	Salinity (psu)	pH	Temperature (°C)	DO (mg L^{-1})	DO (%)
1A	21-Jul-09	15:27:49	0.02	26.6	8.25	34.89	6.44	107.77
		15:28:40	0.52	26.6	8.25	34.25	6.67	110.45
		15:29:30	0.98	26.6	8.27	33.47	6.79	111.1
		15:30:36	1.48	26.5	8.27	32.94	6.72	108.9
		15:33:47	1.98	26.6	8.25	32.86	6.67	107.98
		15:38:32	2.62	26.9	8.15	32.89	4.69	76.18
1B	21-Jul-09	15:49:14	0.01	26.8	8.27	34.88	6.23	104.36
		15:49:48	0.5	26.8	8.27	34.3	6.44	106.8
		15:50:28	1.04	26.8	8.28	33.49	6.49	106.3
		15:50:58	1.46	26.7	8.28	33.05	6.43	104.46
		15:51:31	1.94	26.7	8.28	32.85	6.34	102.76
		15:52:33	2.48	27	8.27	32.92	6.59	107.05
		15:53:16	2.92	27.2	7.87	32.6	2.3	37.26
2A	22-Jul-09	15:13:13	0	26.5	8.23	33.82	5.44	89.48
		15:13:58	0.45	26.6	8.23	33.9	5.68	93.61
		15:14:38	1.01	26.7	8.25	33.57	5.75	94.28
		15:15:55	1.41	26.7	8.25	33.27	5.95	97.21
		15:20:58	1.5	26.7	8.25	33.18	6.16	100.51
2B	22-Jul-09	15:33:38	0	26.5	8.24	33.72	5.66	93
		15:34:07	0.47	26.6	8.24	33.79	5.67	93.33
		15:34:51	0.98	26.6	8.24	33.68	5.81	95.5
		15:35:23	1.41	26.7	8.25	33.33	5.98	97.71
		15:36:08	1.96	26.7	8.24	32.94	5.77	93.83
		15:36:52	2.46	26.9	8.12	32.94	4.81	78.23
3	23-Jul-09	11:07:11	0.22	26.7	8.22	32.64	5.36	86.64
		11:09:18	0.52	26.8	8.2	32.49	5.17	83.35
		11:12:17	0.96	26.7	8.2	32.31	5.18	83.32
		11:14:33	1.52	26.7	8.15	32.16	4.51	72.32
		11:16:42	2	26.7	8.12	32.01	4.15	66.3
		11:21:36	2.51	27	8.05	32.2	2.44	39.3
4	24-Jul-09	17:39:54	0	14.5	8.17	29.41	5.89	84.51
		17:40:30	0.51	25	8.24	31.76	5.77	91.16
		17:41:43	0.95	26.3	8.23	32.49	5.8	93.36
		17:42:20	0.92	26.3	8.23	32.48	5.88	94.68
		17:44:07	1.45	26.6	8.22	32.52	5.61	90.5
		17:45:42	1.75	26.7	8.2	32.59	5.65	91.37
5	25-Jul-09	14:21:13	0.12	17.2	8.19	33.71	6.64	103.49
		14:33:46	0.43	21.4	8.25	32.76	6.15	96.54
		14:23:10	0.51	25	8.26	32.7	6.43	102.86
		14:24:51	1.05	26.3	8.25	33	6.03	97.56
		14:26:40	1.52	26.5	8.25	32.87	5.86	94.71
		14:28:19	2.05	26.6	8.21	32.75	5.15	83.19

Profile	Date	Time	Depth (m)	Salinity (psu)	pH	Temperature (°C)	DO (mg L^{-1})	DO (%)
6A 10 sec	25-Jul-09	15:07:51	0.09	16.9	8.16	32.8	5.84	89.63
		15:08:01	0.11	16.9	8.16	32.8	5.8	88.92
		15:08:11	0.19	16.9	8.16	32.8	5.77	88.43
		15:08:21	0.29	16.8	8.15	32.8	5.8	88.97
		15:08:31	0.35	18.3	8.19	32.67	5.79	89.38
		15:08:41	0.46	22.6	8.23	32.41	5.7	89.61
		15:08:51	0.57	24.6	8.25	32.89	5.82	93.14
		15:09:01	0.74	25.4	8.26	33.15	5.94	95.87
		15:09:11	0.96	25.7	8.26	33.08	5.94	96.04
		15:09:21	1.1	26.2	8.25	33.11	5.9	95.71
		15:09:31	1.23	26.4	8.25	33.15	6.02	97.73
		15:09:41	1.35	26.5	8.25	33.13	6.11	99.2
		15:09:51	1.47	26.5	8.25	33.01	6.09	98.73
6B 05 sec	25-Jul-09	15:10:41	0.14	16.9	8.15	32.78	5.88	90.08
		15:10:46	0.15	16.9	8.15	32.78	5.86	89.83
		15:10:51	0.23	16.9	8.15	32.78	5.79	88.85
		15:10:56	0.27	16.9	8.15	32.78	5.79	88.82
		15:11:01	0.28	16.9	8.15	32.78	5.82	89.33
		15:11:06	0.37	17	8.15	32.78	5.83	89.46
		15:11:11	0.4	18.9	8.16	32.69	5.8	89.74
		15:11:16	0.45	23.7	8.26	32.43	5.79	91.52
		15:11:21	0.52	24.7	8.27	32.86	5.95	95.25
		15:11:26	0.58	24.6	8.27	32.94	6.06	97.08
		15:11:31	0.7	25.2	8.27	33.06	6.06	97.64
		15:11:36	0.85	25.4	8.26	33.12	6.04	97.55
		15:11:41	0.93	25.8	8.26	33.09	6.04	97.59
		15:11:46	1.05	26.1	8.25	33.1	6.01	97.26
		15:11:51	1.14	26.2	8.25	33.11	5.96	96.63
		15:11:56	1.24	26.4	8.26	33.15	5.94	96.38
		15:12:01	1.31	26.4	8.26	33.15	6.06	98.43
		15:12:06	1.36	26.4	8.25	33.04	6.13	99.36
		15:12:11	1.47	26.5	8.25	33	6.07	98.28
		15:12:16	1.56	26.5	8.25	32.95	6.04	97.8

Figure S10. pH and DO % Correlation from Geochemistry Profiles

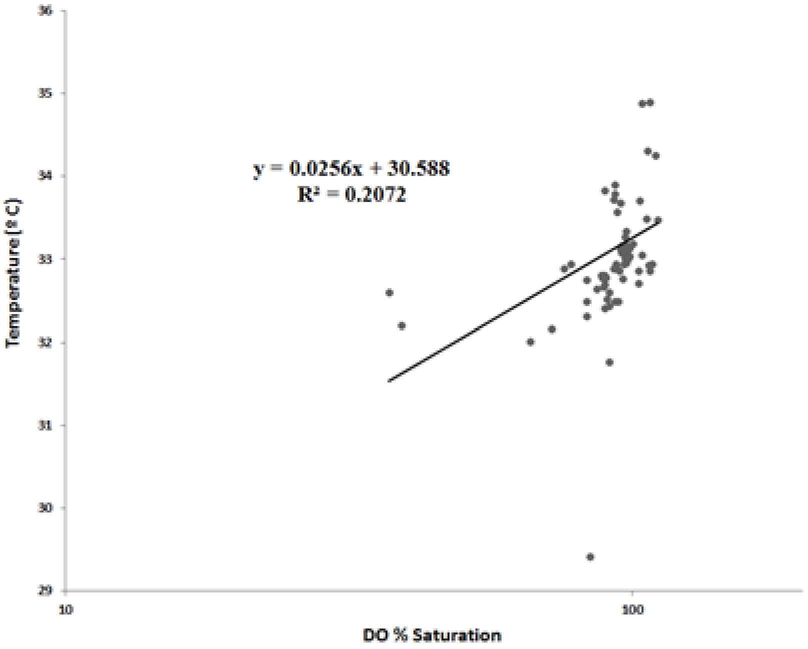

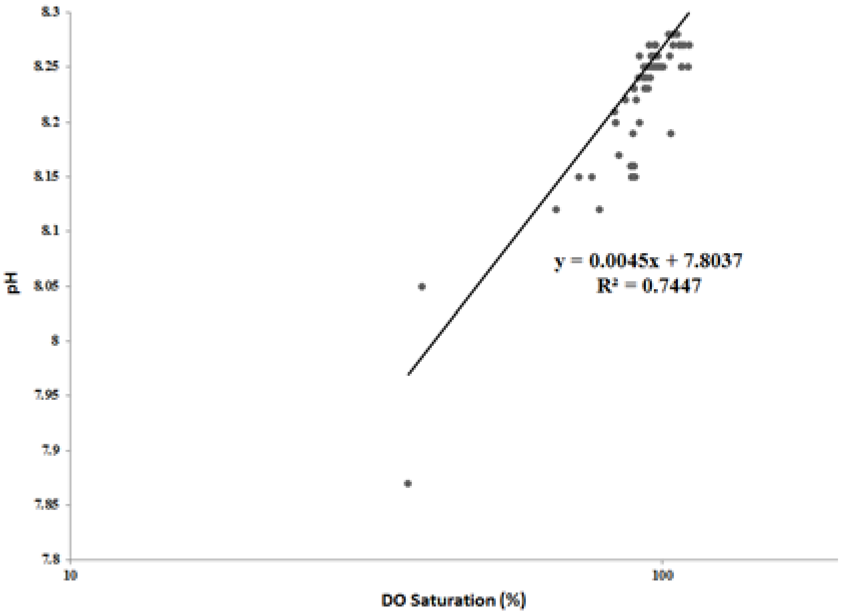

Graphic representation of the positive correlation between measured pH values and DO% saturation, with DO% reported on a log scale.

Filter System

We used a filter to check and collect plankton and other minute organic material in Lib Pond. Below is one such filter:

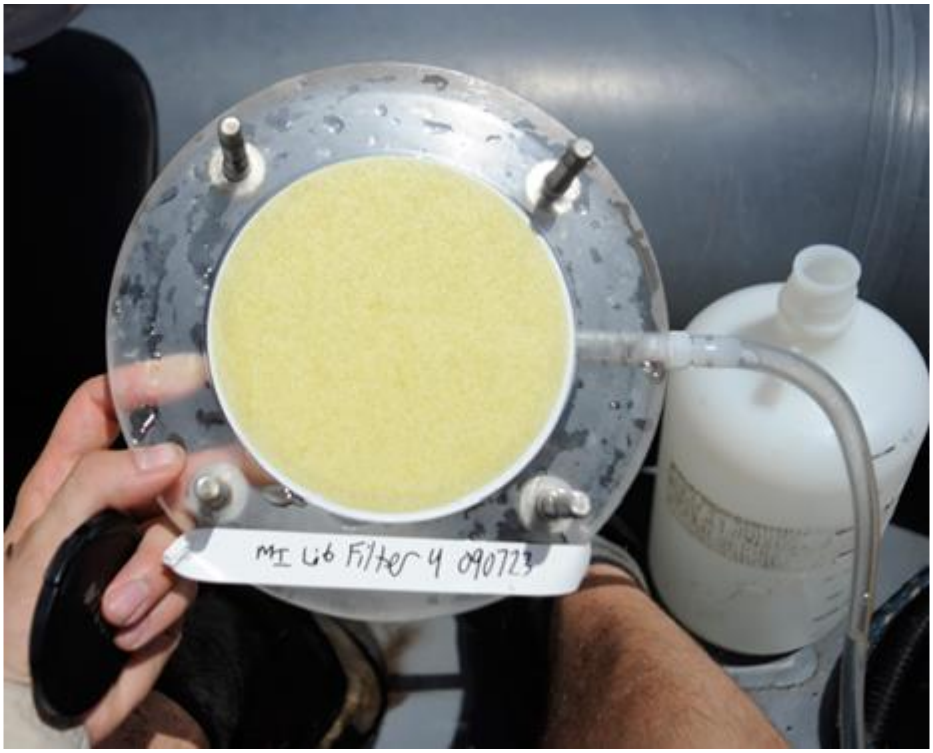

Figure S11.

P-E Box Model

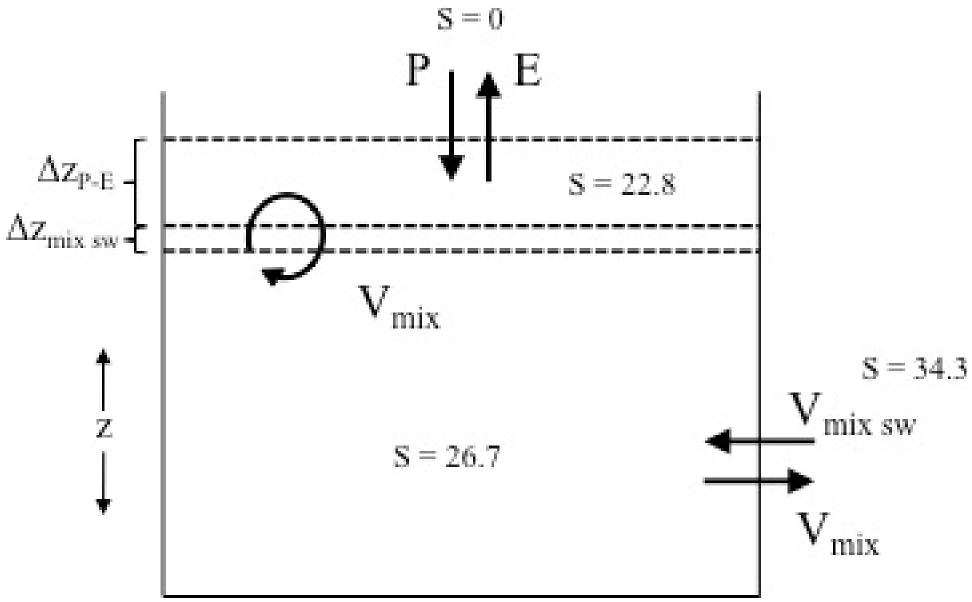

Figure S12. Box model representation of Lib Pond reservoir (Vmix), showing inputs of evaporation (E) and seawater (Vmix sw), outputs of precipitation (P) and ebb flow (Vmix), water depth, z, and lake level changes (Δz) associated with P-E and tidal flow.

Sediment Core Data

Lib Pond Bivalve Identification

Santa Barbara Museum of Natural History

Department of Invertebrate Zoology, 2559 Puesta del Sol Road, Santa Barbara, California 93105 USA

http://www.sbnature.org

From: Daniel Nelson
School of Oceanography
University of Washington
Box 355351
Seattle WA 98195
USA

Accession Number: 2009-040
Accession Date: 20 Nov 2009
Received by:
Approved by: Paul Valentich-Scott

Digitally signed by Paul Valentich-Scott
DN: cn=Paul Valentich-Scott, o=Santa Barbara Museum of Natural History, ou=Dept. of Invertebrate Zoology, email=pvscott@sbnature2.org, c=US
Date: 2009.11.23 08:07:04 -08'00'

Cat. #	Taxon	Quantity	Locality
**149234**	***Ctena bella***	Dry 1<br>Wet 0	Oceania, Marshall Islands, Lib Island, lagoon; from sediment core; 08°18'52"N, 167°22'51"E; 0-2 m.

Figure S13.

The dominant bivalve collected in the Lib Pond sediment cores is Ctena Bella, as identified from a physical sample at the Santa Barbara Museum of Natural History (excerpted certification shown above.)

Algal Mat Example

The below example shows an algal mat which was often at the surface of the cores, including several at Lib. Here, a picture of a similar core from Mejit Island, also in the Marshalls, is shown as a visual example (taken on July 28th, 2009, evidenced by the 090728 date.) It is approximately 0.5 cm thick. It is held up by a plastic sectioner.

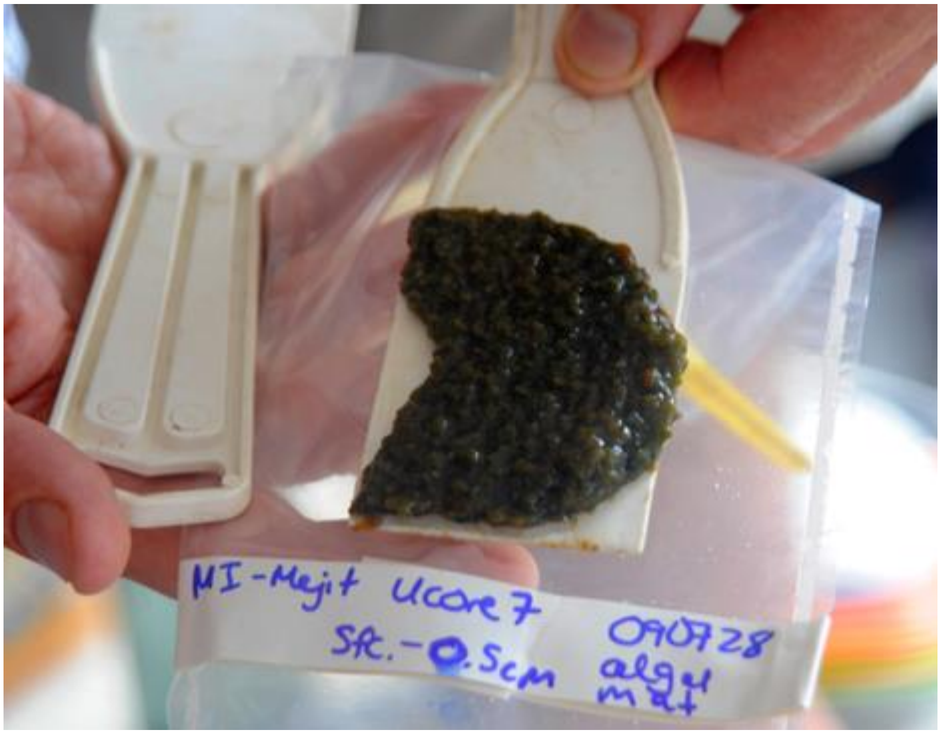

Figure S14.

Table S8. Lib Pond Sediment Core Description (Example: MI-Lib Ucore 6)

Site Name	MI-Lib 6
Core	MI- Lib Ucore 6
Coordinates	
Total core length (cm)	88

Depth (cm)	Description
sfc - 1	green algal material overlaying unconsolidated, amorphous organic material: rust-colored
1- 49*	rust-colored amorphous OM, terrigenous organic fragments; sulfitic odor
49-59	reddish-tan silt
59	sharp boundary layer
59-71	brown silt; appearance of bivalve shells
71	sharp boundary layer
71-80	amorphous mutlishade tan silt; small calcareous fragments
80-88	highly turbated, dark brown silt; extensive bivalve abundance

*denotes end of sectioned region

Site Name	MI-Lib 6
Core	MI- Lib Ucore 6
Coordinates	
Total core length (cm)	728

Depth	Description
100-104	unconsolidated rust-colored amorphous OM; sulfitic odor
104	diffuse boundary layer
104-148	brown/rust-colored silt, progressively lighter in color; diffuse banding red and brown in color
148-149	sharp red-brown bedding layer
149-165	tan amorphous silt; bivalve detected at 150 cm
165-168	indistinct bedding plane boundary
168-180	dark brown amorphous silt; discontinuous layers of tan silt
180-184	extensive bivalve abundance
184-203	decline in bivalve abundance
203-208	sharp increase in bivalve abundance
208	diffuse boundary layer
208-217	rust-colored silt; diffuse laminae; bivalve band at 213 cm
217-223	indistinct bedding plane boundary
223-230	light brown silt; small presence of bivalves
230-238	sharp increase in bivalve abundance
238-246	sharp decline in bivalve abundance; dark brown silt
246-250	sharp increase in bivalve abundance
250-256	sharp decline in bivalve abundance
256-260	sharp increase in bivalve abundance
260-275	abrupt disappearance of bivalve shells; dark reddish-brown laminated silt

275-284	dark brown amorphous silt
284-287	bivalves present
287	diffuse boundary layer
287-326	dark brown silt; diffuse lamination; carbonaceous macrofossil at 295 cm; white carbonate fragments
326-330	indistinct bedding plane boundary
330-342	tan sand layer; discontinuous beds of darker silt
342	white carbonate band
343	diffuse boundary layer
343-362	dark brown amorphous silt; bivalve layer 352-356 cm
362	sharp boundary layer
362-373	tan sand layer; extensive bivalve abundance
373-377	abrupt disappearance of bivalves
377-389	shell abundance resumed
389-393	brown silt bedding layer
393-395	tan sand layer, sharp bedding plane boundary
395	sharp bedding plane
395-410	dark brown silt; thin bedding
410	sharp bedding plane
410-412	sand; no bivalves present
412	bivalves present
412-476	lagoon sand; bivalves present
476	indistinct bedding plane boundary
476-728	finer, white lagoon sediment; decreased bivalve abundance
728	coral basement reached; coral fragments reported

Lib Sediment Core Radiocarbon Dating

Table S9. Sample Location and Characteristics

Core	Core Top (cm)	Core Bottom (cm)	Sample Midpointcore Depth (cm)	Total Depth (cm)	Sample Material
Lib 6 Piston Core 1	100	200	51.25	151.25	Twig
Lib 6 Piston Core 2	200	300	2	202	Wood
Lib Ucore 5 - Core Tube	118	233	6	124	Twig
Lib Ucore 5 - Core Tube	118	233	95.25	213.25	Woody Material
Lib Ucore 5 -Sectioned Material	0	118	20.5	20.5	Bark Material
Lib 6 Piston Core 3 10-11 cm	300	400	10.5	310.5	Bulk Sediment
Lib Ucore 7 - 3-4 cm	0		3.5	3.5	Bulk Sediment
Lib Ucore 7 - 68.5-69.5 cm	0		69	69	Bulk Sediment
Lib Ucore 7 - 10 cm	0		10	10	Macro-Bark/Wood
Lib Ucore 7 - 42-43 cm	0		42.5	42.5	Macro-Wood
Top of Core				0	

Table S10. Sample Radiocarbon Dates with Error Estimate

Core	Age (14C yrs)	14C error	Med. Cal Age	Max Cal. Age	Max. Error	Min Cal. Age	Min. Error
Lib 6 Piston Core 1	2374		2405	2655	250	2338	67
Lib 6 Piston Core 2	2307		2336	2356	20	2183	153
Lib Ucore 5 - Core Tube	1124		1018	1068	50	965	53
Lib Ucore 5 - Core Tube	2362		2365	2461	96	2338	27
Lib Ucore 5 - Sectioned Material	3736		4090	4223	133	3982	108
Lib 6 Piston Core 3 10-11 cm	2219	27	2230	2328	98	2152	78
Lib Ucore 7 - 3-4 cm	890	30	809	908	99	734	75
Lib Ucore 7 - 68.5-69.5 cm	1945	30	1895	1969	74	1823	72
Lib Ucore 7 - 10 cm	1250	30	1206	1273	67	1082	124
Lib Ucore 7 - 42-43 cm	835	30	742	793	51	685	57
Top of Core	0		-59	-59	0	-59	0

Note: the top of the core was not measured but assumed modern.

Lib Pond Age Model

A simplified age model that assumes a linear interpolation between the radiocarbon dates of our sediment cores, indicates that some of the sediment collected in the Lib Pond cores is over a thousand years old. IEEE indicates samples dated by the Xi'an AMS Center at the Insitute of Earth Environment, the Chinese Academy of Sciences (url: http://english.ieexa.cas.cn/rh/rd/200907/t20090719_23981.html). 'CAMS' indicates samples dated by the Center for Accelerator Mass Spectrometry at the Lawrence Livermore National Laboratory (https://cams.llnl.gov/).

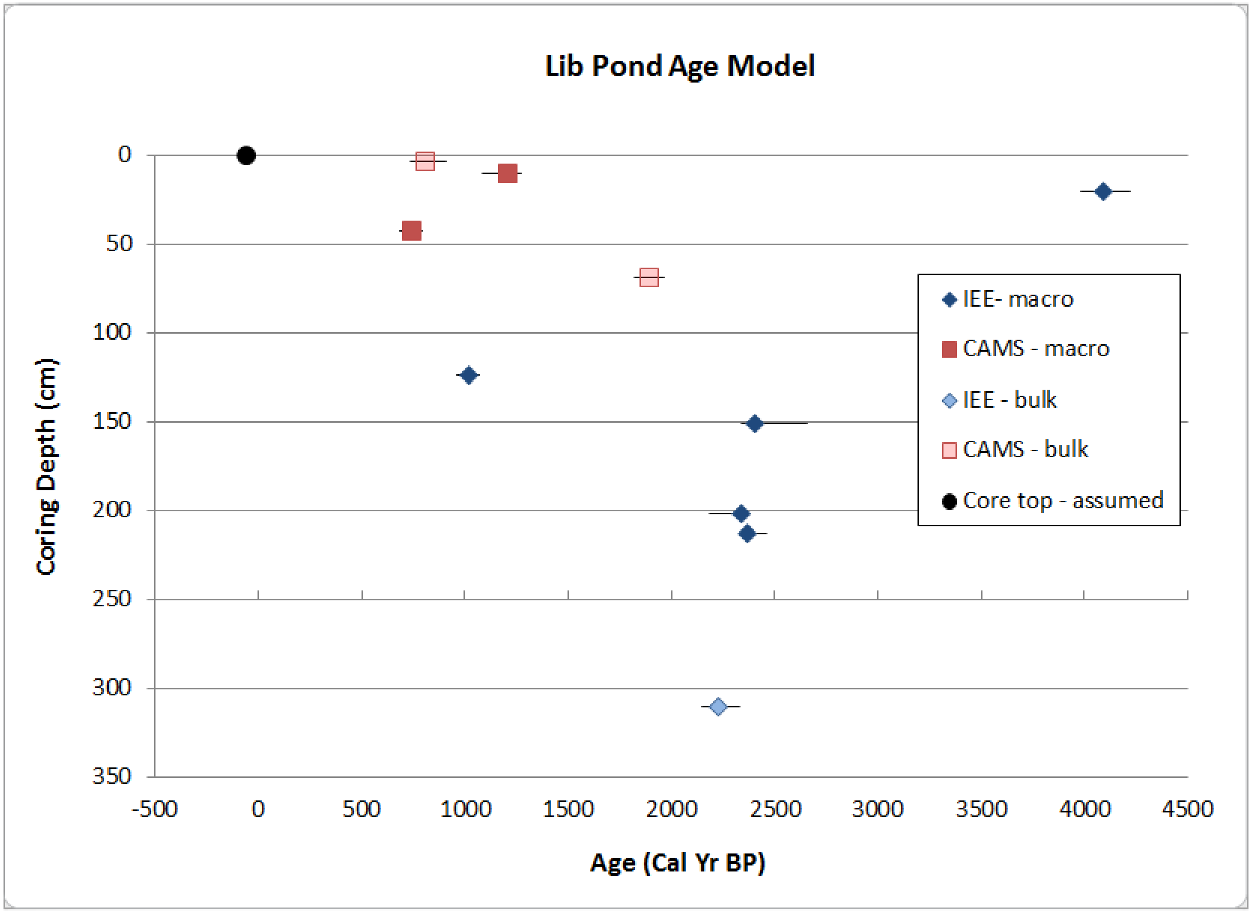

Figure S15.

Photos of the Sediment Core Collection Process

1) A lengthy Ucore is transported to the shoreline after being collected in Lib Pond. Julian Sachs holds while Alyssa Atwood (front) and S. Nemiah Ladd (back) row.

2) Julian Sachs points out sharp sediment transition in a Lib Pond Sediment Core to Dan Nelson. Visible in this photograph are several major sediment type transitions, from reddish to green with shells, to tan.

3) Fran Janny prepares to section a lengthy Ucore, the sediment color changes which can clearly be seen going down the core (i.e. back in time.)

Figure S16. 1

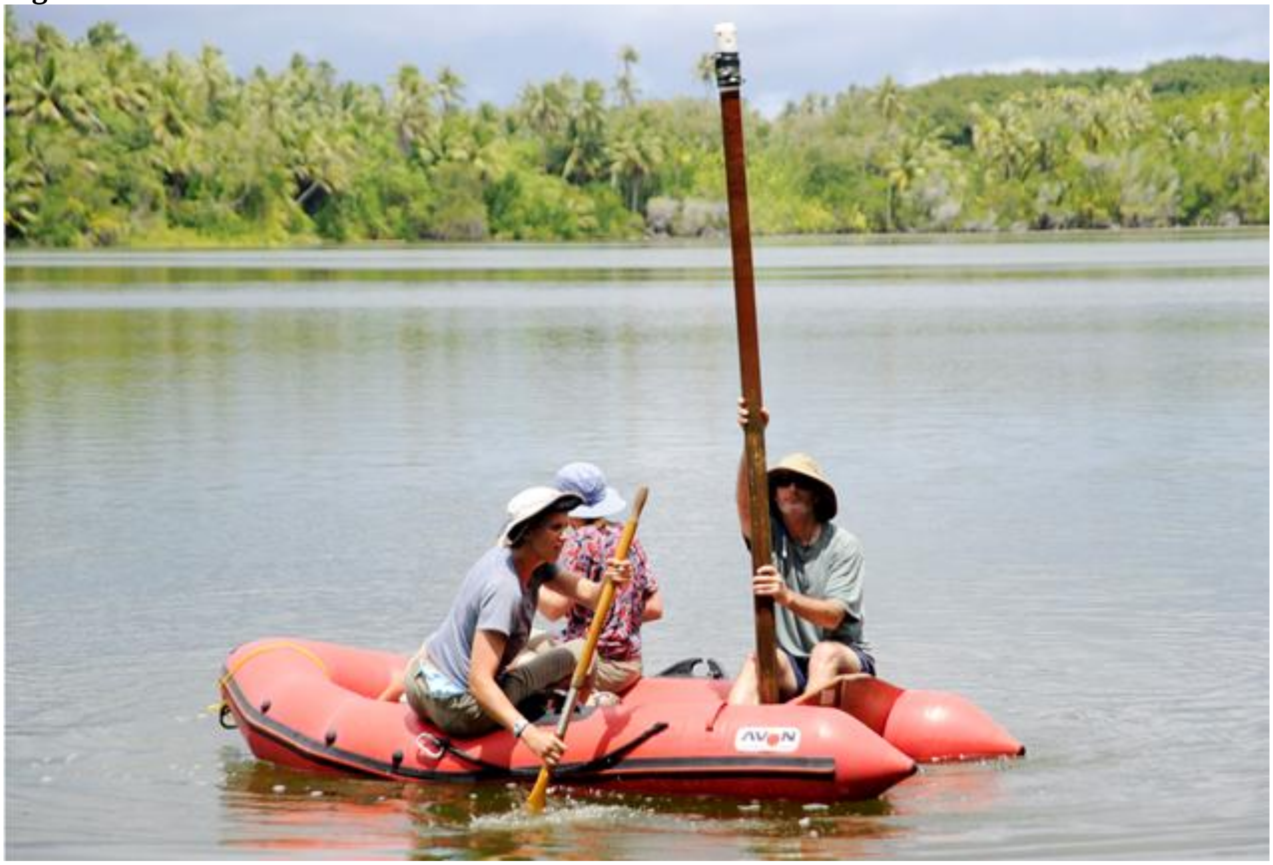

Figure S17. 2

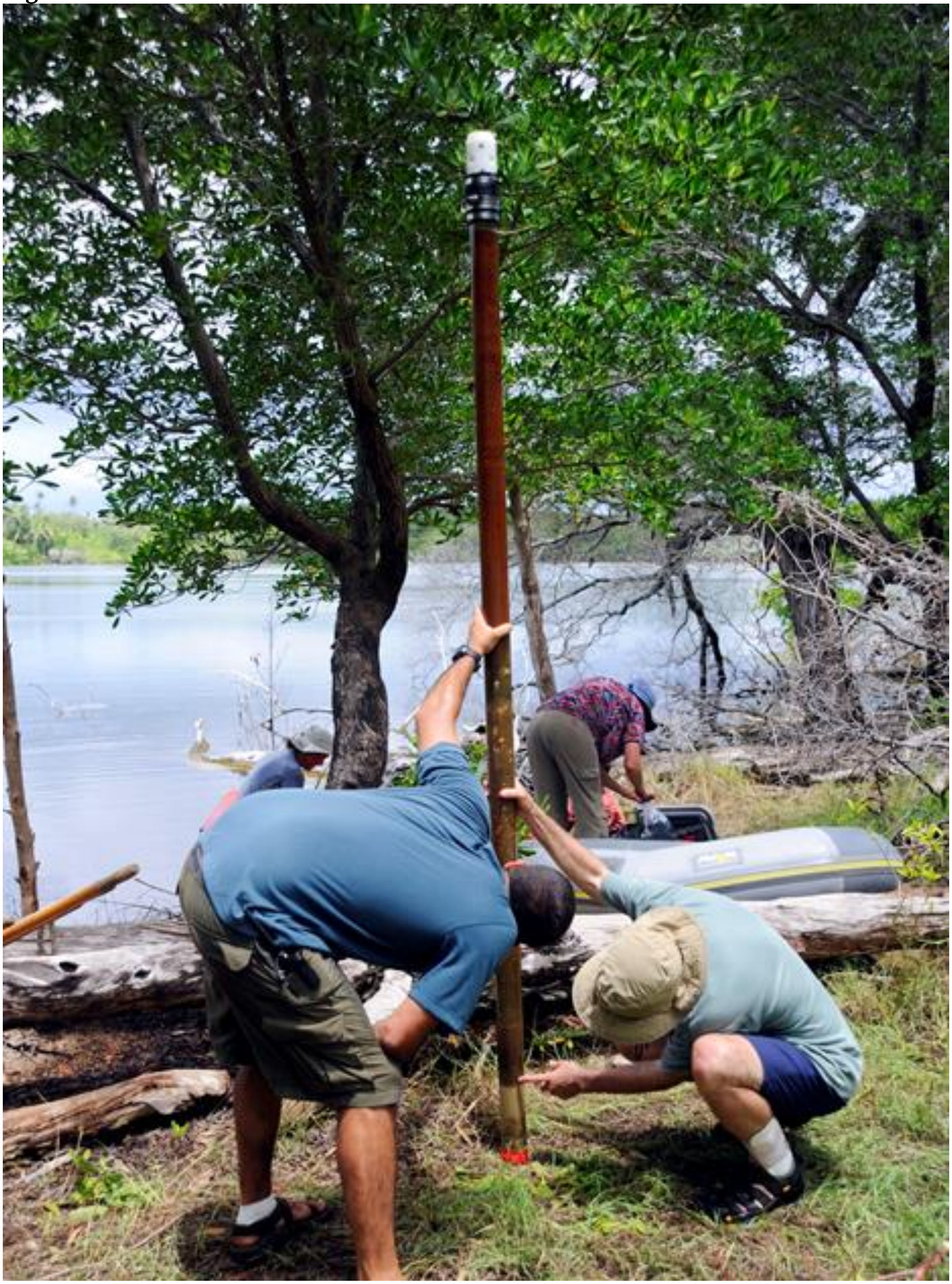

Figure S18. 3

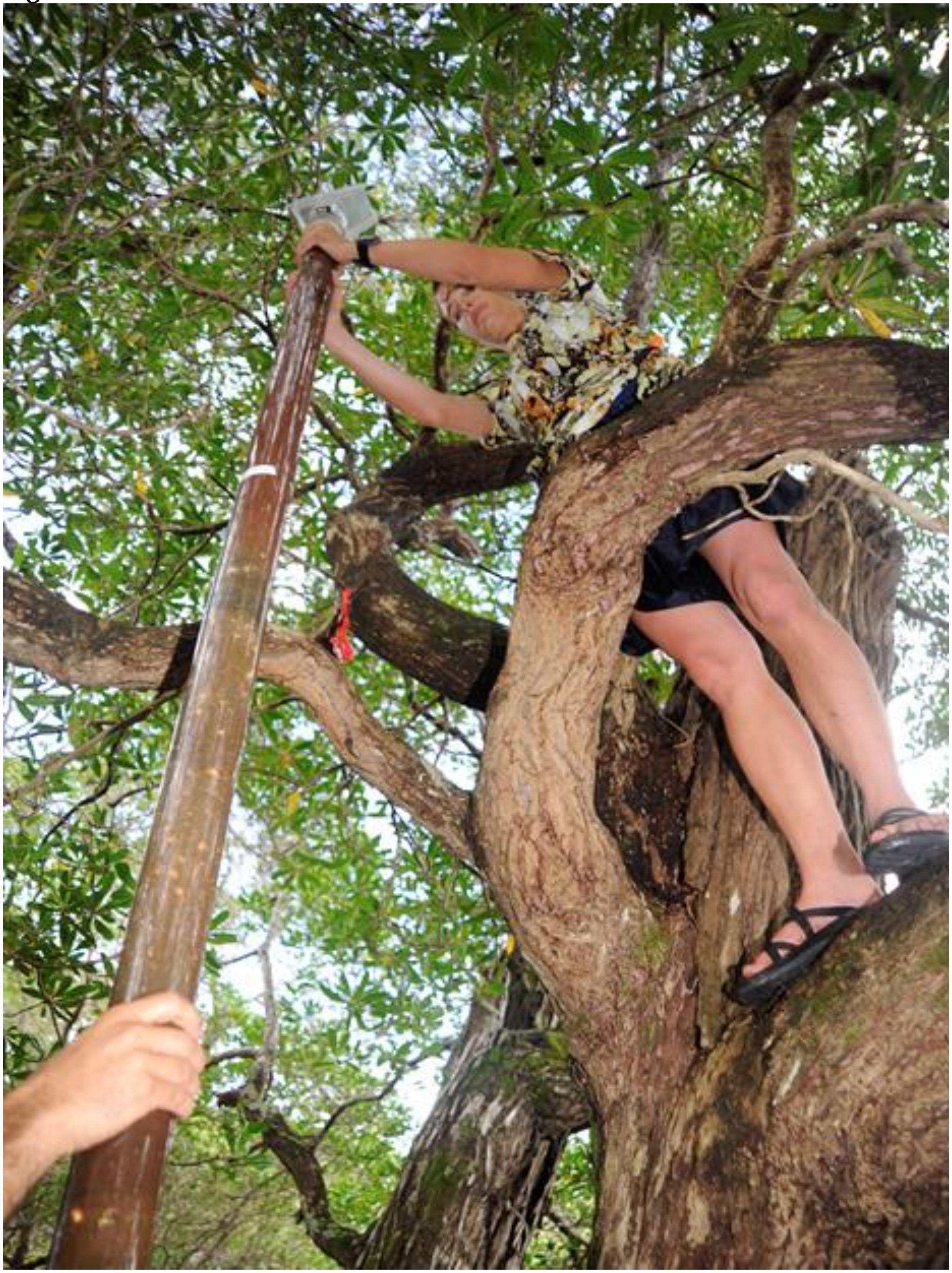

Hi-res Ucore Photograph Sequence

Lib Pond Ucore 5 Sequence, Photographed in Intervals Before Sectioning

Below are close up images, in sequence, of the fifth Ucore from Lib Pond (LI-5), after it was collected but before it was sectioned (bagged in 1 cm intervals, so that the core would not be disturbed in transit back to Seattle from the Marshall Islands). With the exception of one image (with 180 cm), all pictures show 150 cm worth of sediment core. All pictures are from the top down, in sequence (note the numbers on the ruler which overlap in each instance).

For numbering purposes for this sequence, the scale we use is the length of the tape measure; thus 0 cm starts at the top of the core (and the most recent deposition, chronologically).

Figure S19. Lib Ucore 5, Photo 1 of 17

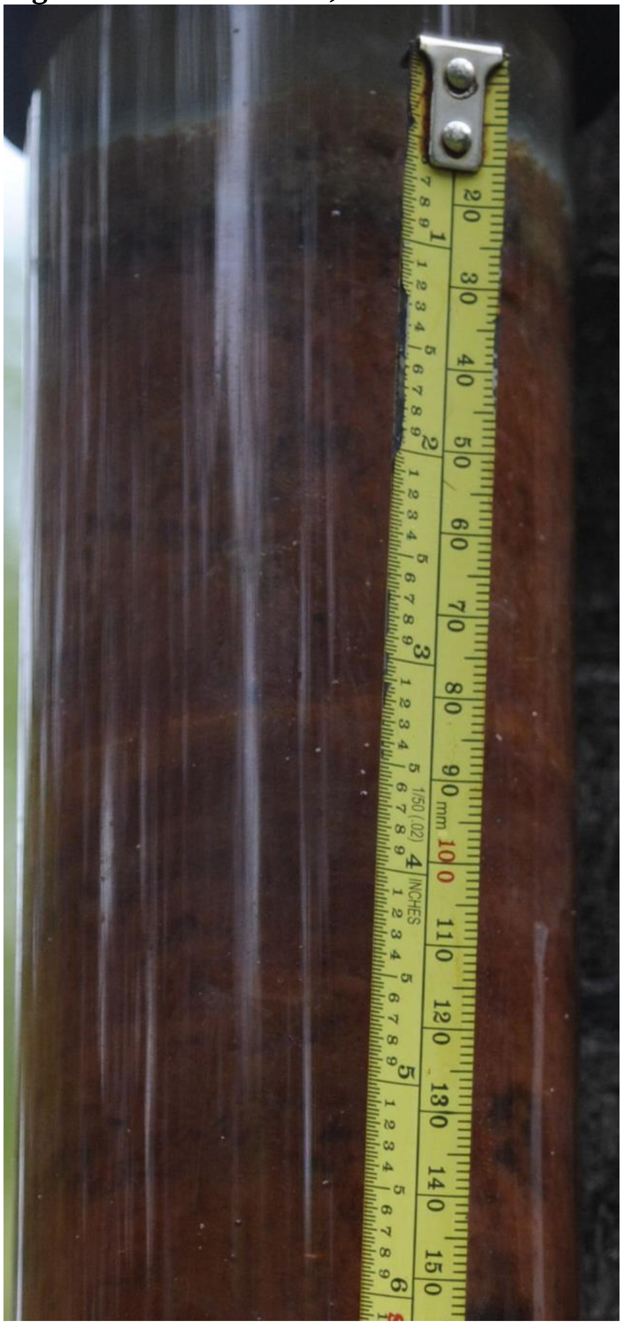

0 – 150 cm. Note the green algal mat from ~ 0-20 cm.

Figure S20. Lib Ucore 5, Photo 2 of 17

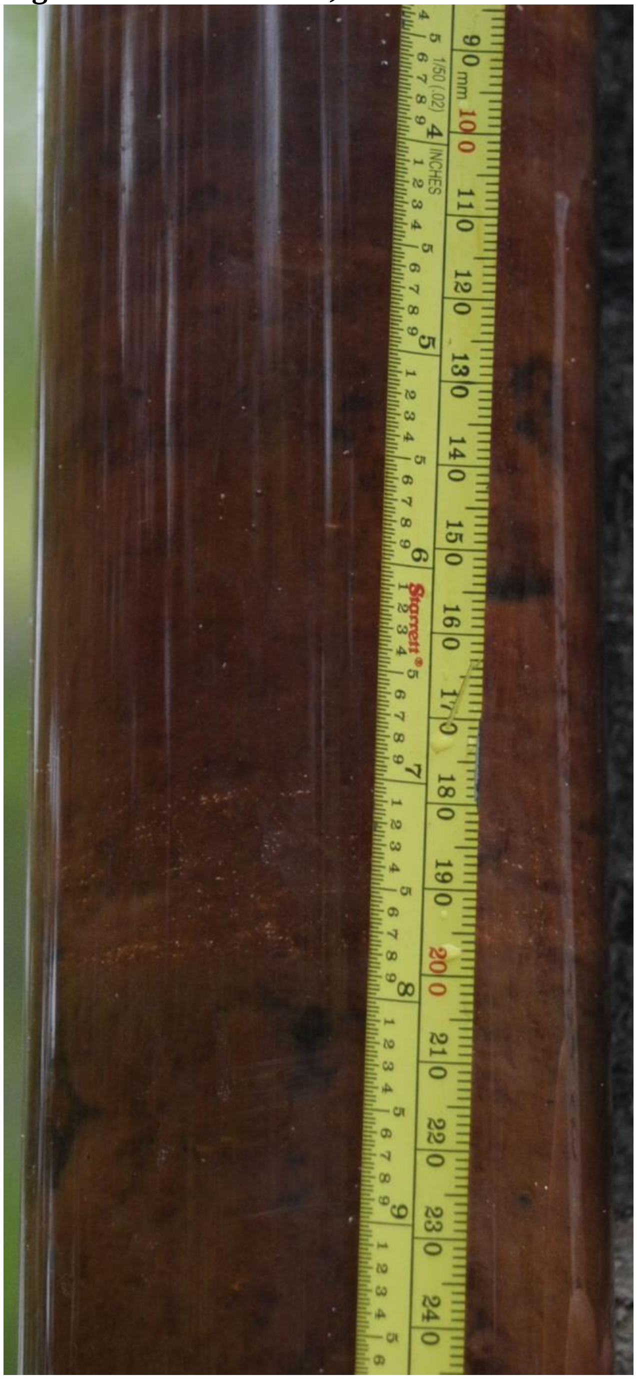

90 – 240 cm.

Figure S21. Lib Ucore 5, Photo 3 of 17

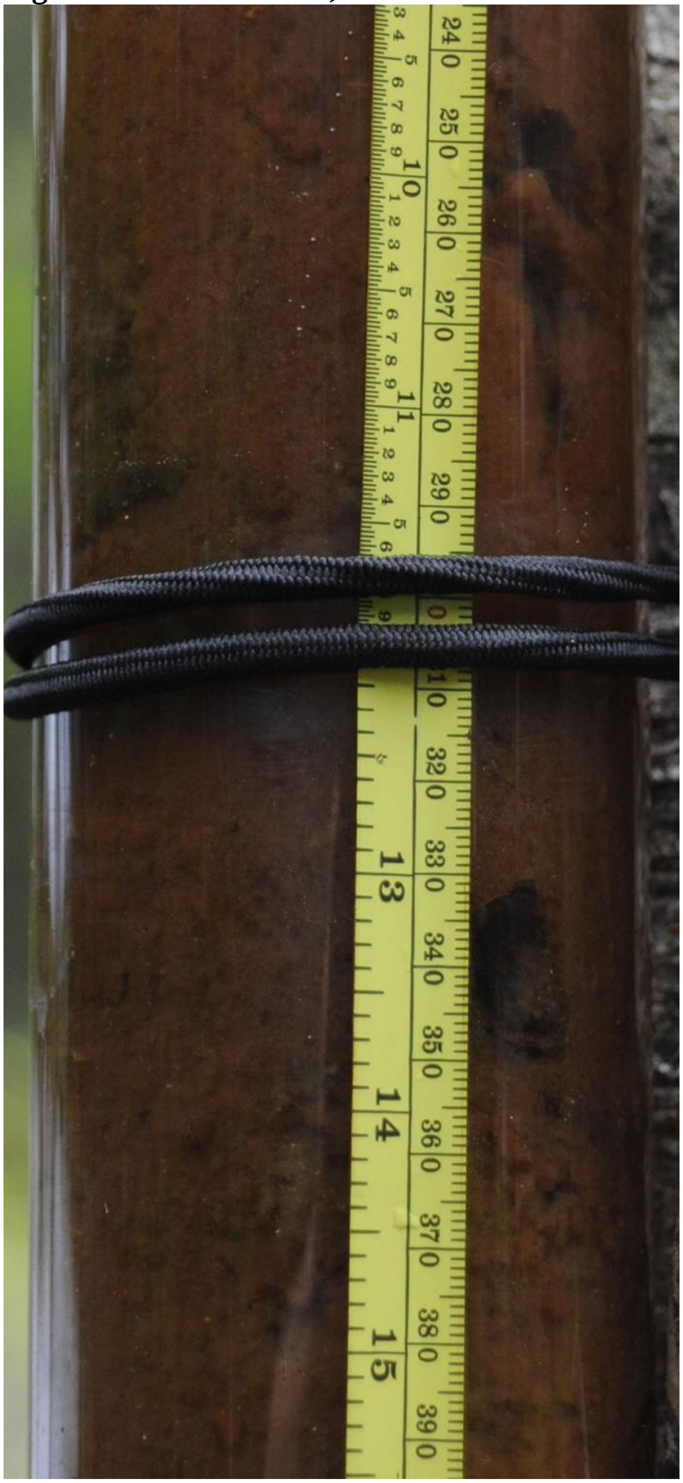

240-390 cm.

Figure S22. Lib Ucore 5, Photo 4 of 17

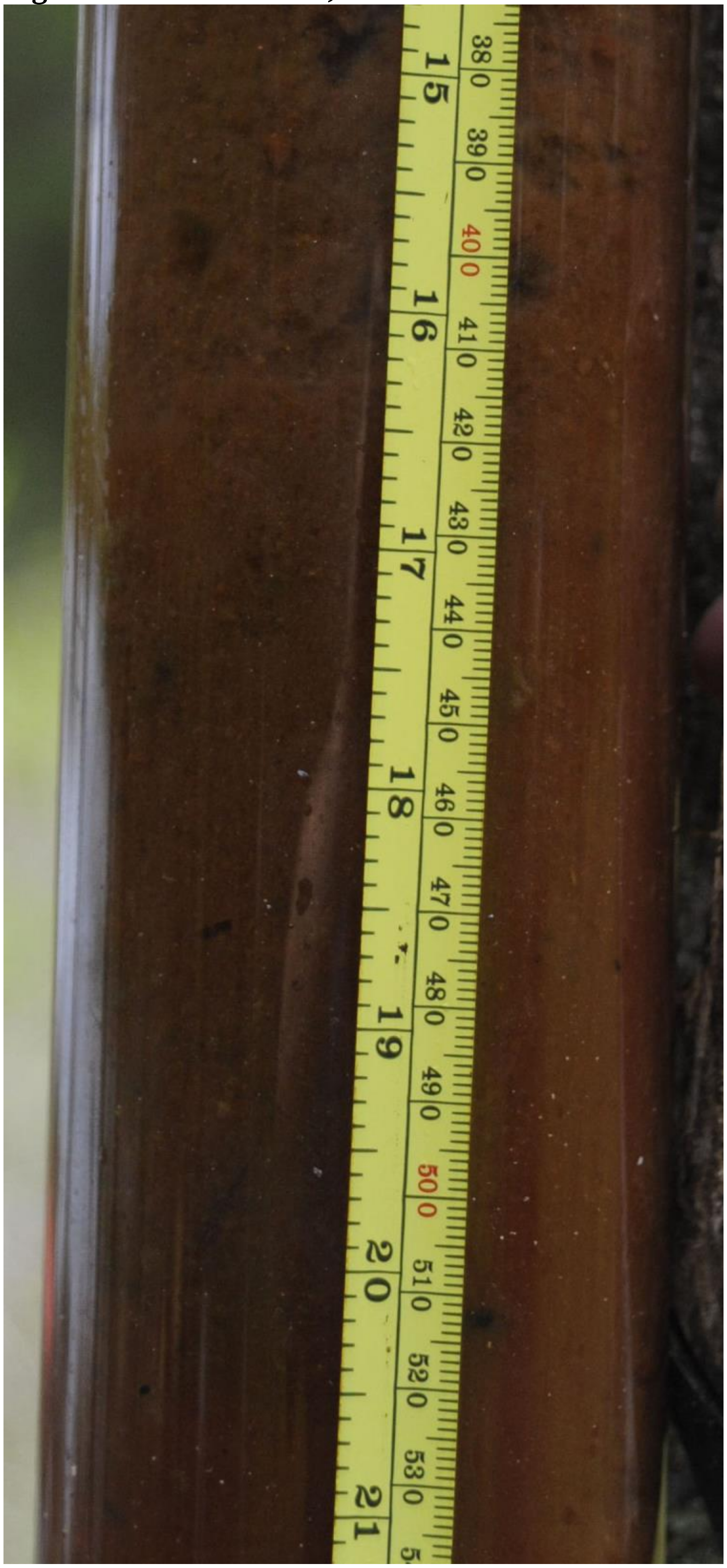

380-530 cm.

Figure S23. Lib Ucore 5, Photo 5 of 17

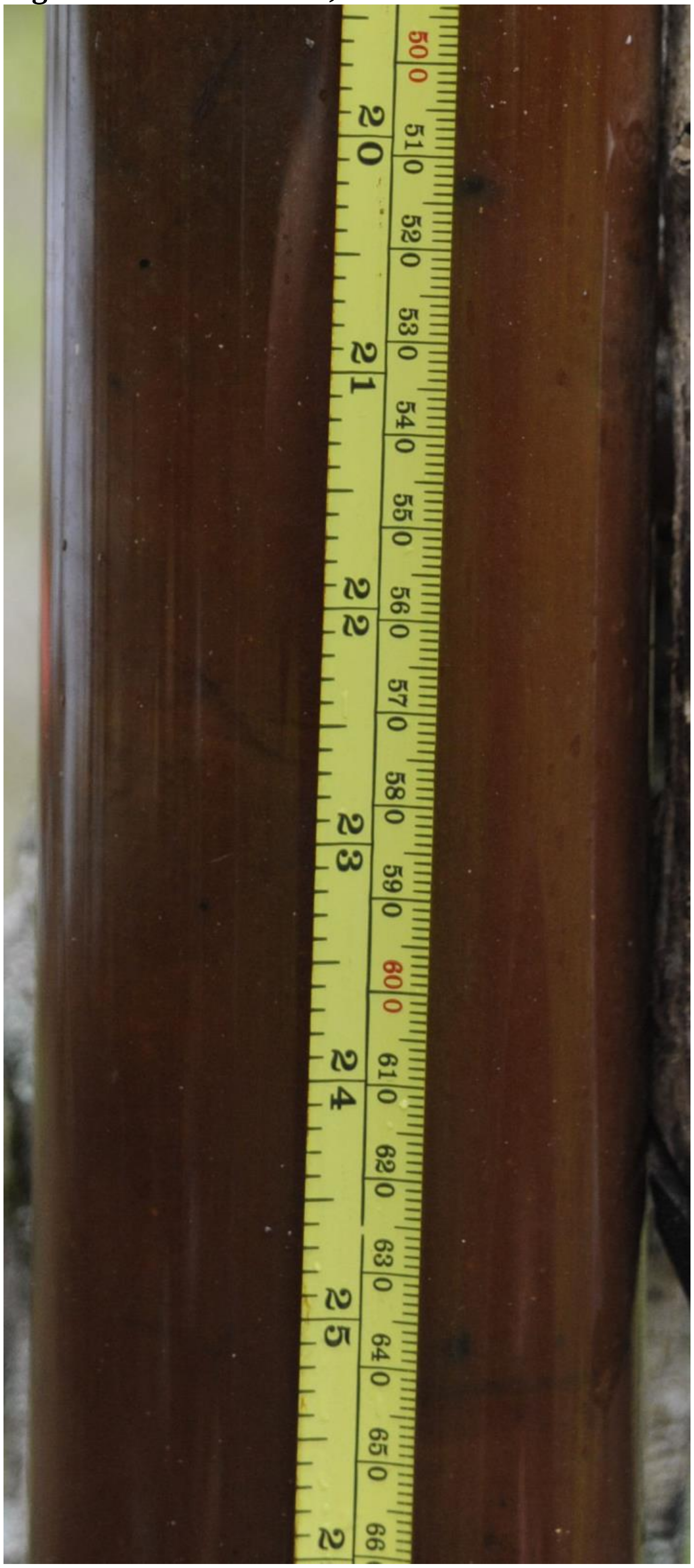

500-650 cm.

Figure S24. Lib Ucore 5, Photo 6 of 17

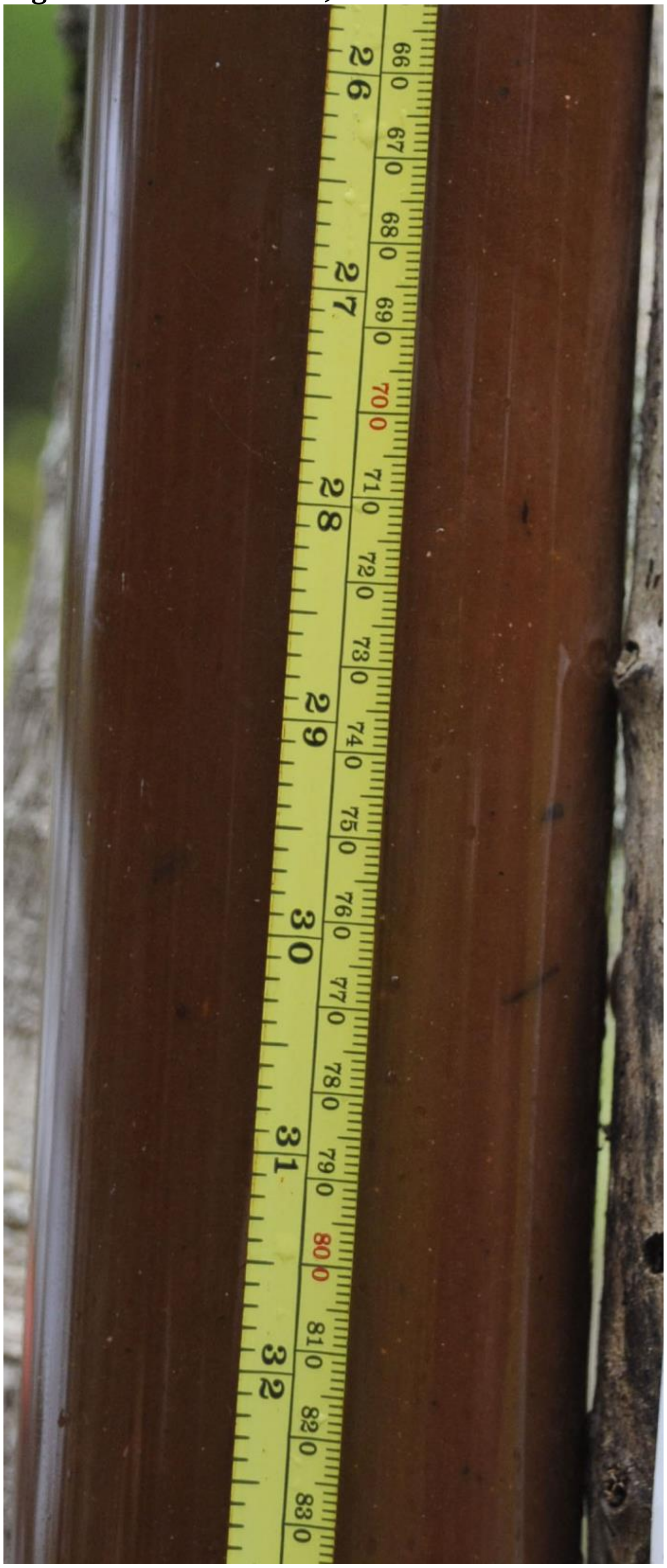

660-830 cm.

Figure S25. Lib Ucore 5, Photo 7 of 17

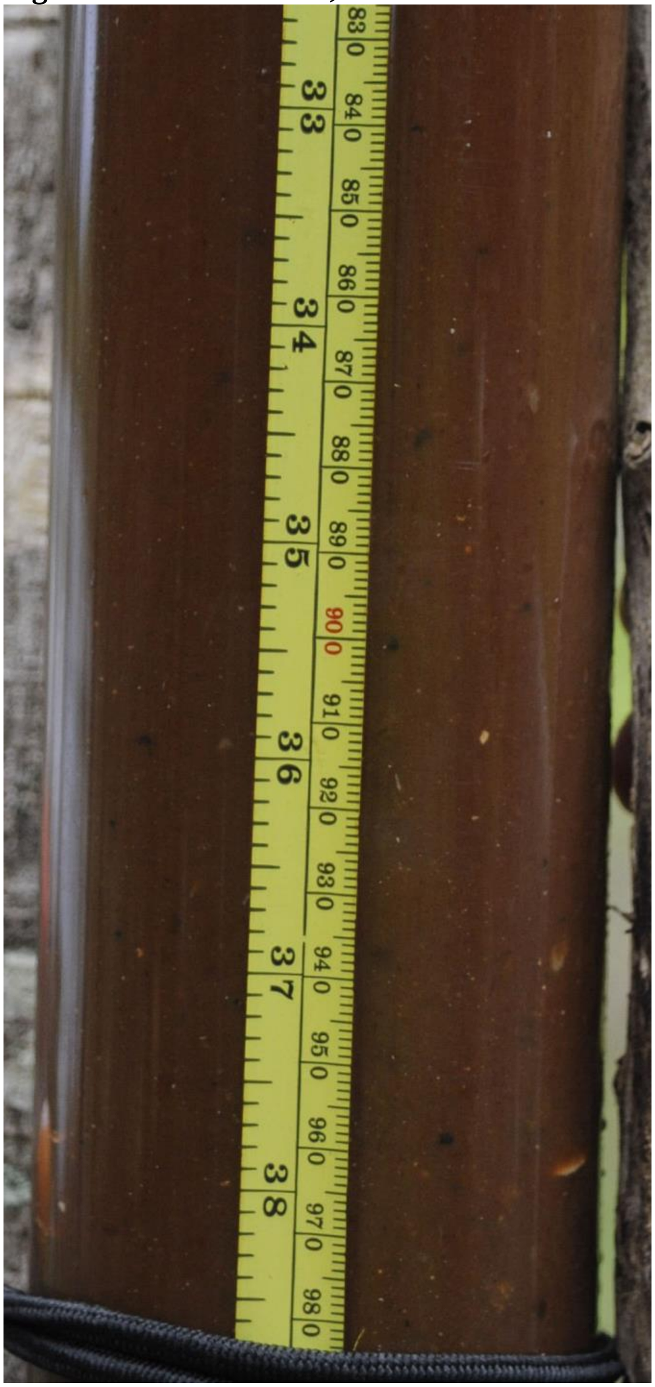

830-980 cm. Toward 940 cm, bivalves begin.

Figure S26. Lib Ucore 5, Photo 8 of 17

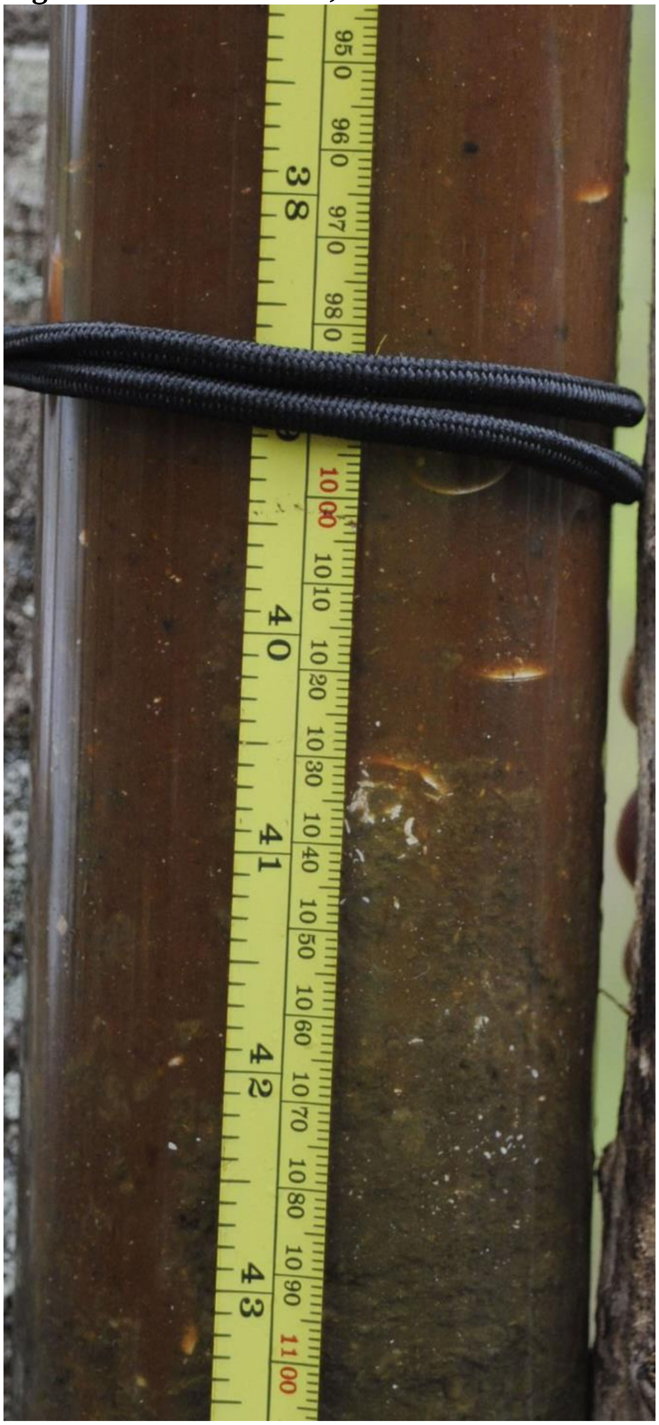

950-1100 cm. A transition from red to dark green takes place 1030-1090 cm.

Figure S27. Lib Ucore 5, Photo 9 of 17

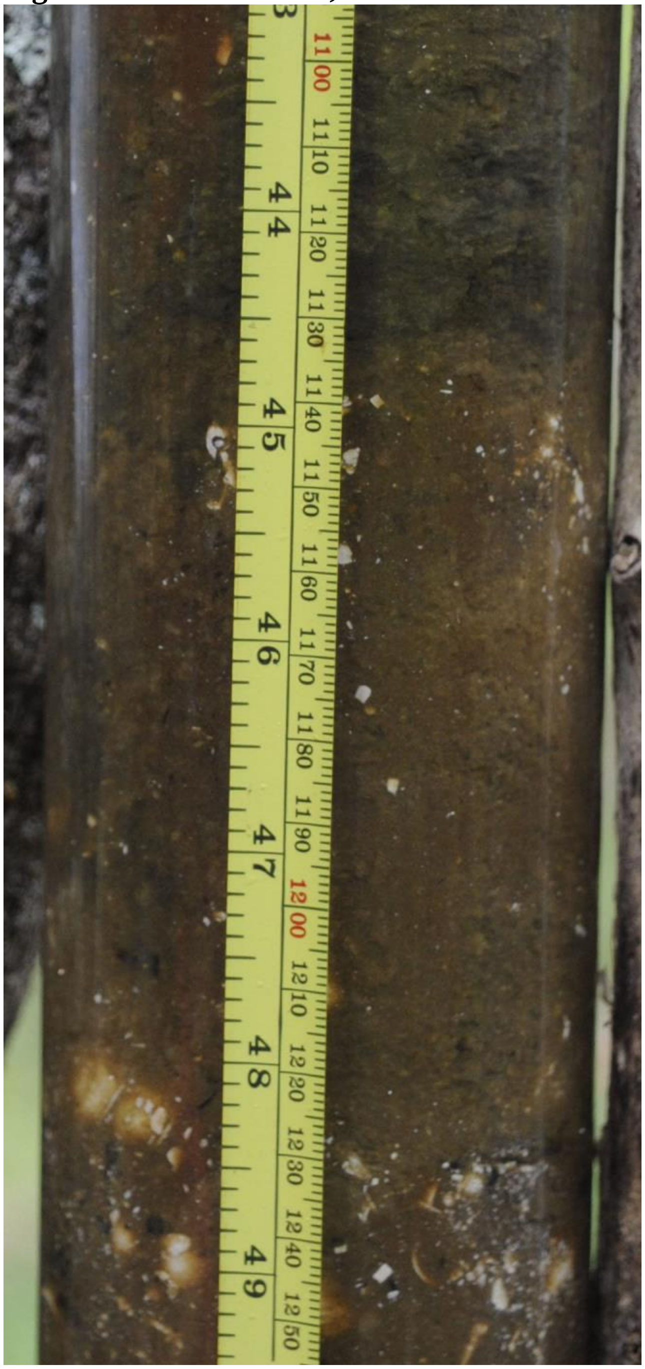

1100 – 1250 cm. The last hint of red is lefthand side, 1090-1100 cm.

Figure S28. Lib Ucore 5, Photo 10 of 17

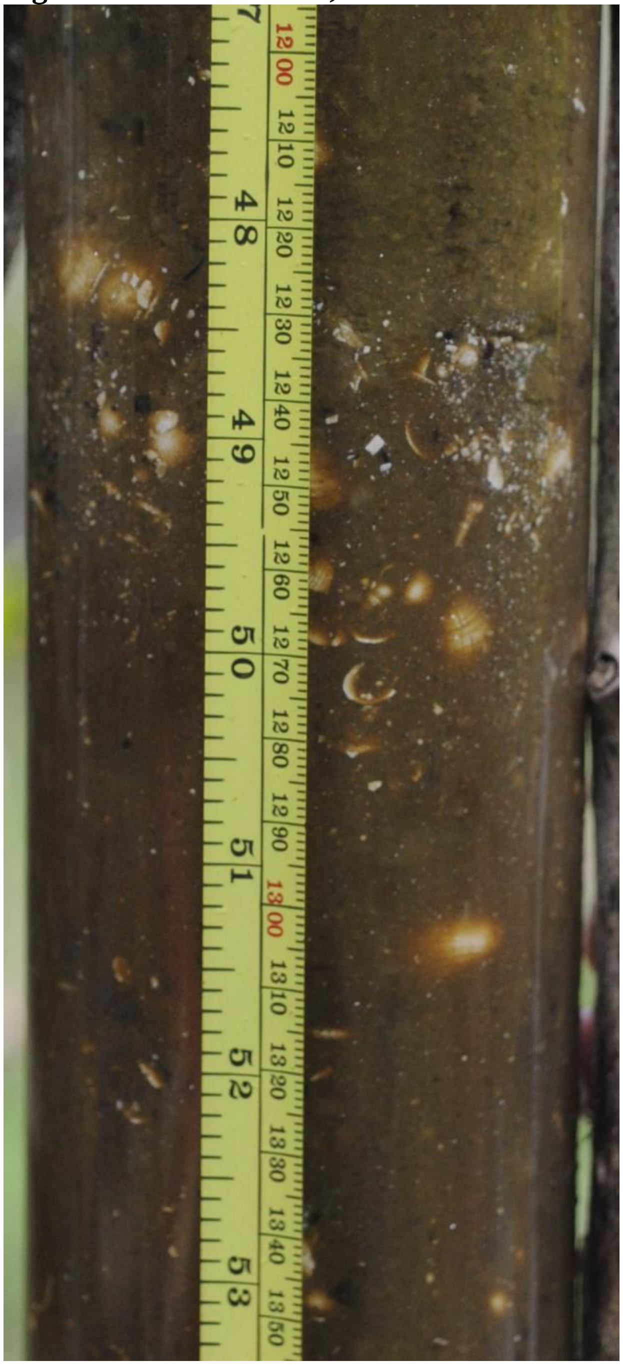

1200-1350 cm. Lots of bivalves begin at 1220 cm.

Figure S29. Lib Ucore 5, Photo 11 of 17

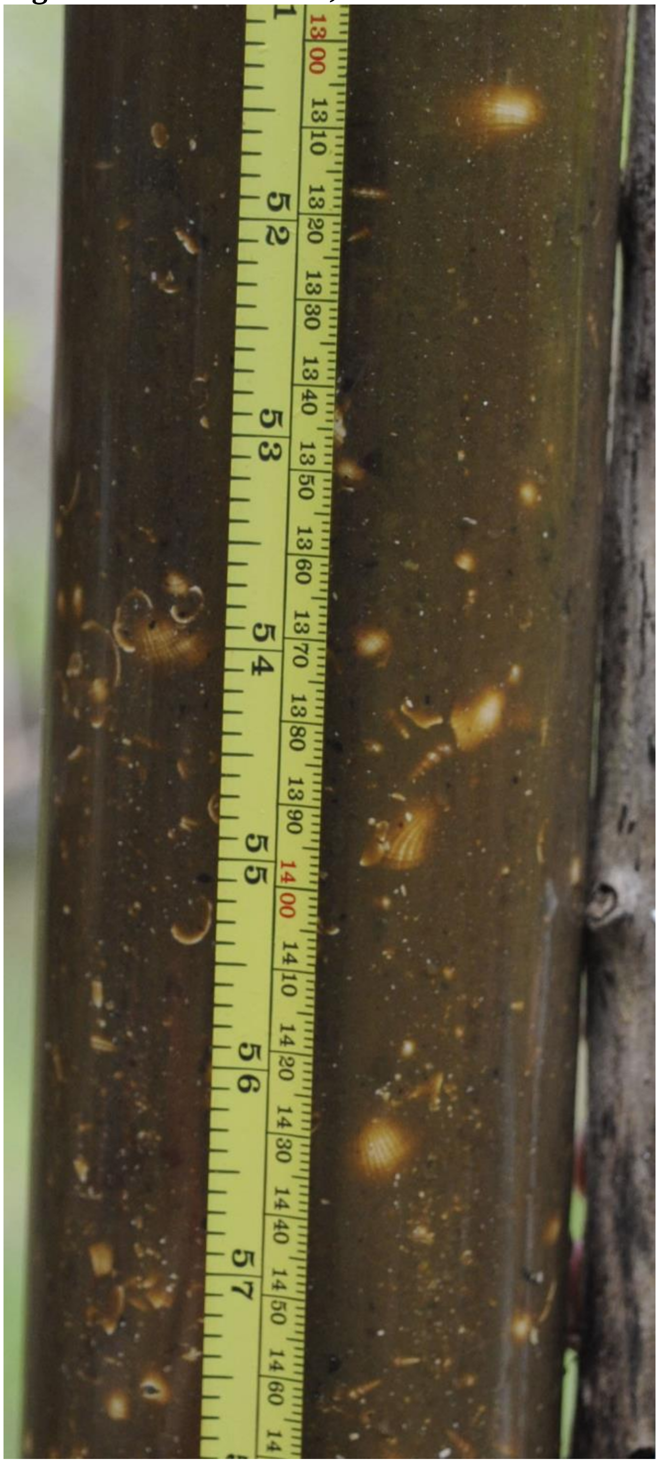

1300-1460 cm.

Figure S30. Lib Ucore 5, Photo 12 of 17

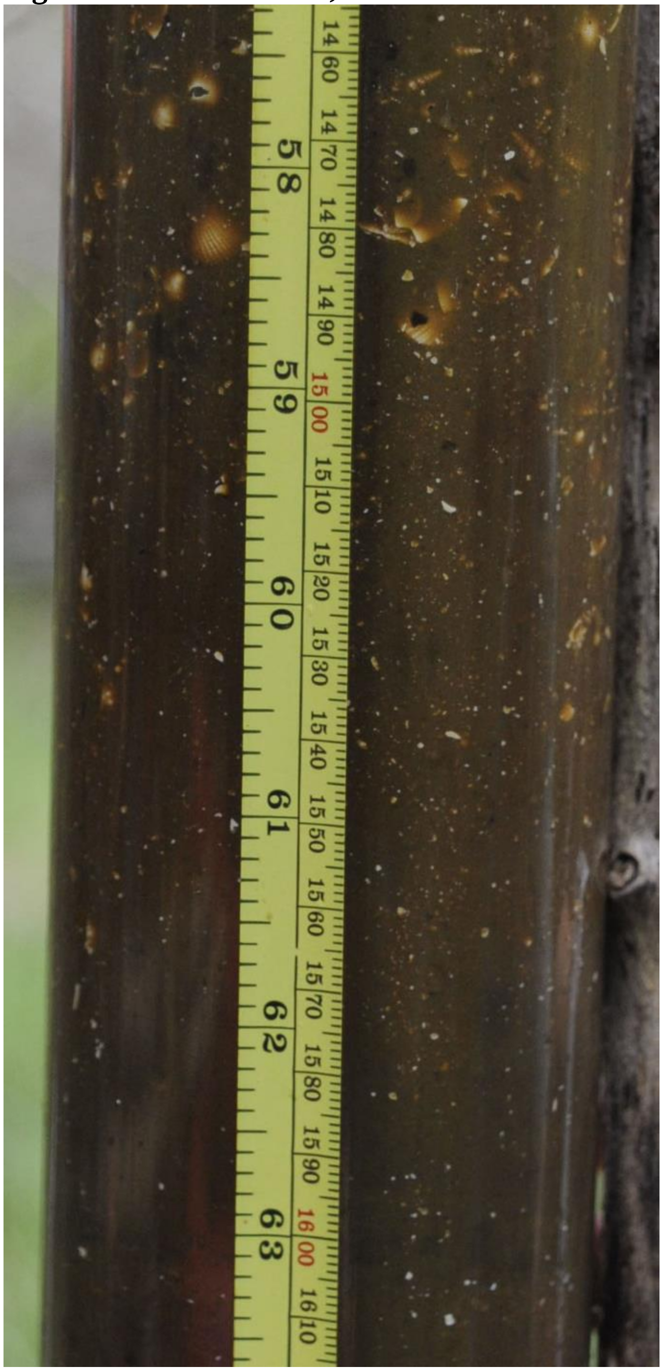

1460-1610 cm. A more consistent, homogenous dark green sediment compared to Photos 9 and 10. Bivalves dissipate ~ 1550 cm.

Figure S31. Lib Ucore 5, Photo 13 of 17

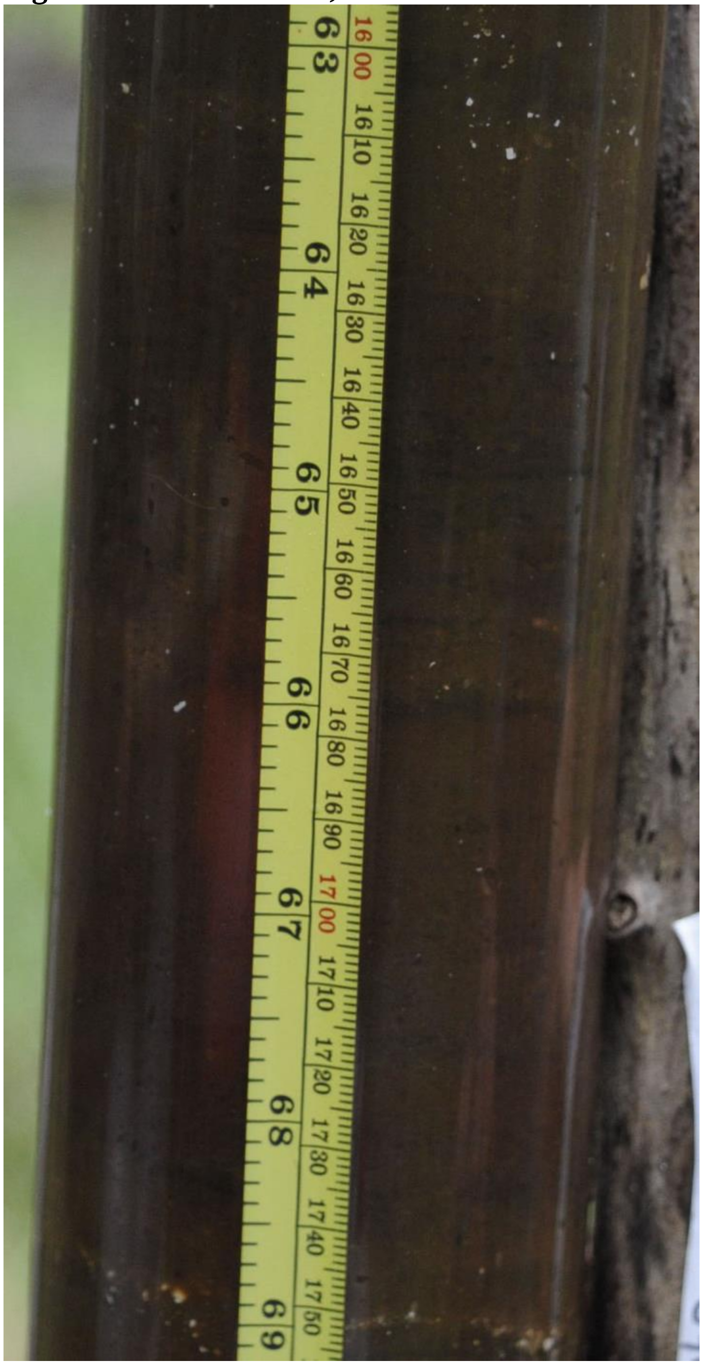

1600-1750 cm. At 1750 cm, a layer transition.

Figure S32. Lib Ucore 5, Photo 14 of 17

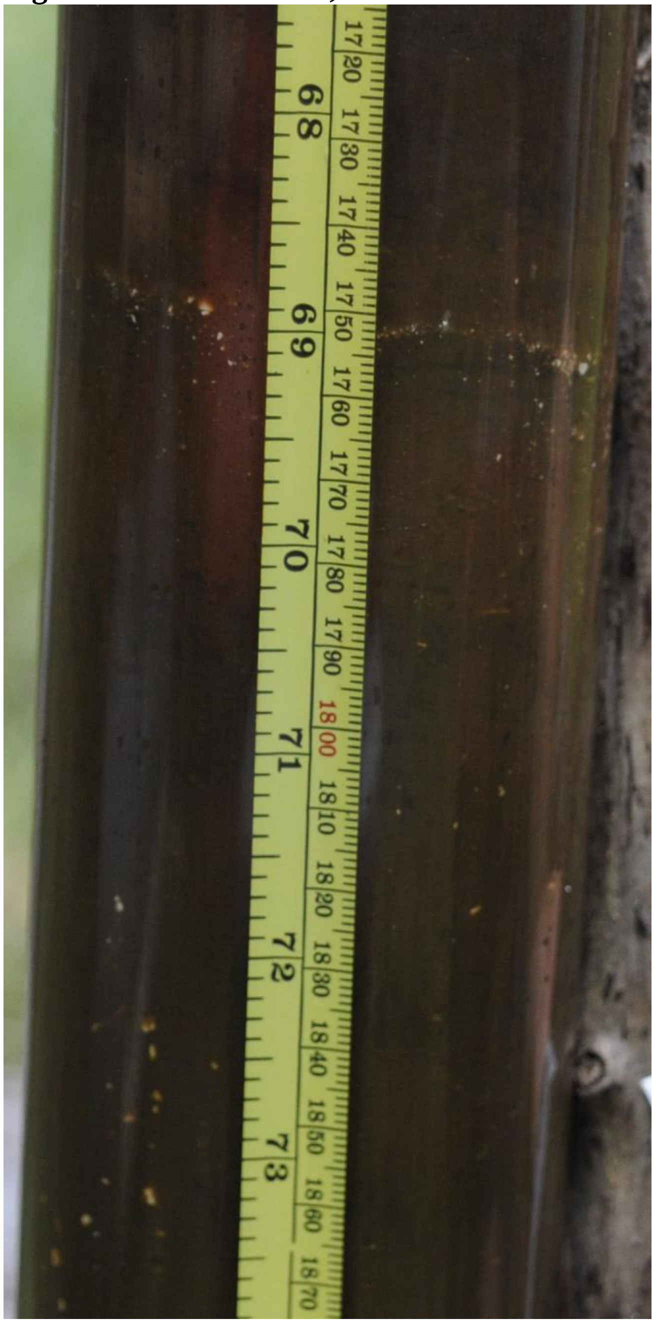

1720-1870 cm. Again, note the layer transition at 1750 cm.

Figure S33. Lib Ucore 5, Photo 15 of 17

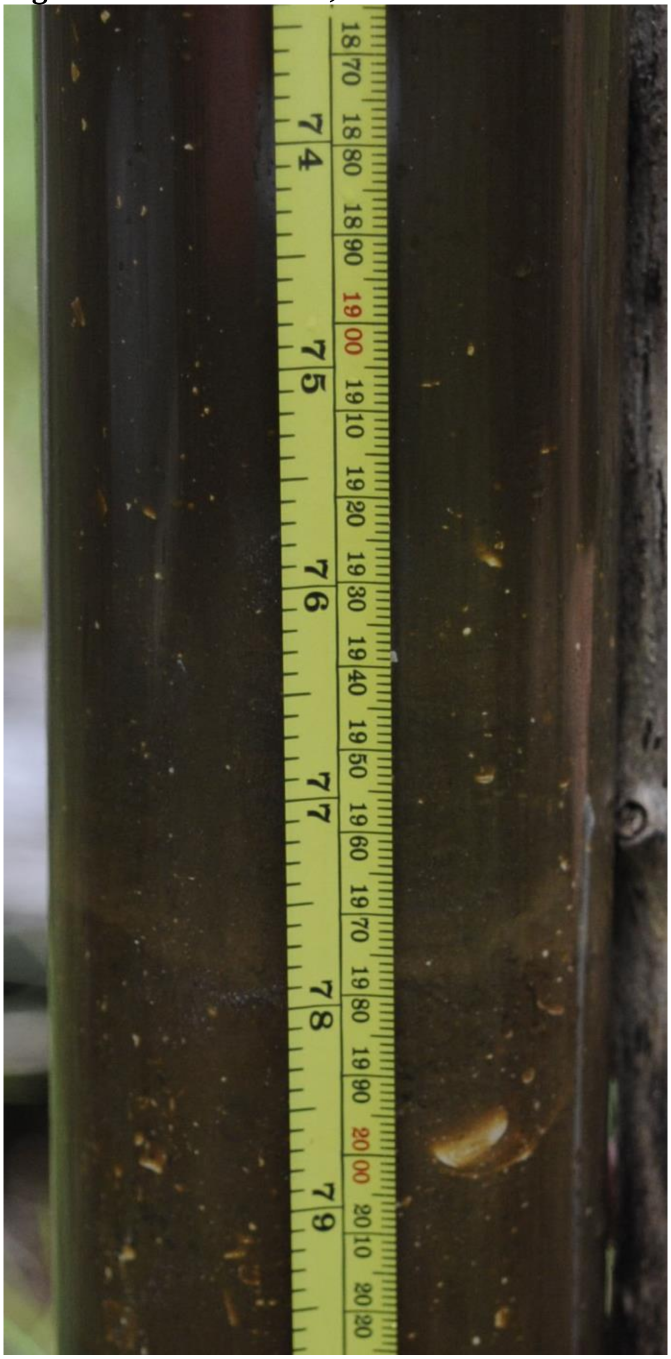

1870-2020 cm. Further down, more bivalves appear. A subtle color transition appears at 1980 cm.

Figure S34. Lib Ucore 5, Photo 16 of 17

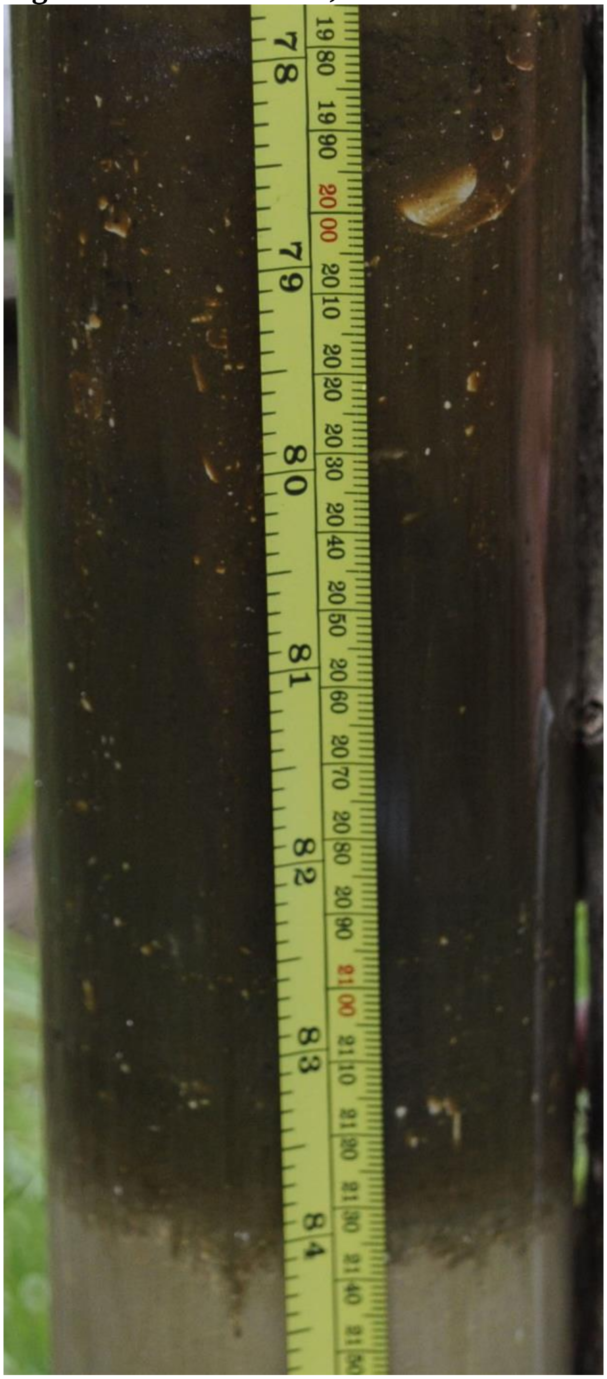

1980-2150 cm. A sharp transition between a dark green sediment color and white-gray silty sand abruptly begins at ~ 2135 cm.

Figure S35. Lib Ucore 5, Photo 17 of 17

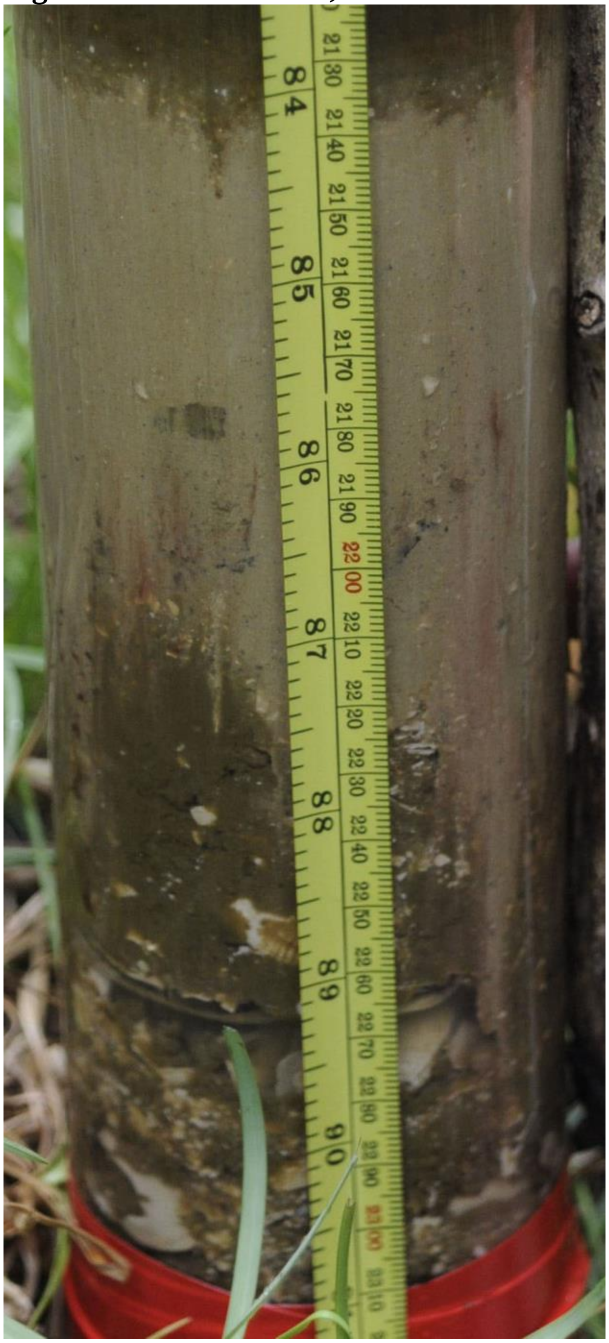

2130 – 2310 cm. Note the sand layer ends partly at 2200-2270 cm. Significant bivalve and broken shells appear at the end of the core, compared to earlier.

Sediment Core Side-by-Side Photograph Sequence

Figure S36. Lib Pond Sediment Cores Side-by-Side

Here we line up Lib Pond sediment cores in chronological order. PC = Piston core. UC = Ucore. The numbers indicate the order in which the cores were taken, and are consistent with the numbering system throughout the rest of the paper. For example, Lib 6 PC is the piston core taken at the Lib 6 site, while Lib UC6 is the ucore taken at the Lib 6 site. Note that one piston core – the sediment – was taken in several sections to comprise the entire length, owing to the maximum length of the core – the hollow metal cylinder which captures and brings the sediment to the surface.

Correlation lines linking likely similar scenarios (thus allowing coherent chronology to be established across different sediment core lengths) are detailed by dash lines.

Radiocarbon dates are also marked in their respective locations, some of which were used for the simplified age model.

For the original image files of this lineup (not embedded in this PDF), contact the author.

radiocarbon sample

piston core correlation line (more confident)

U core correlation line (less confident)

Lib 11 PC

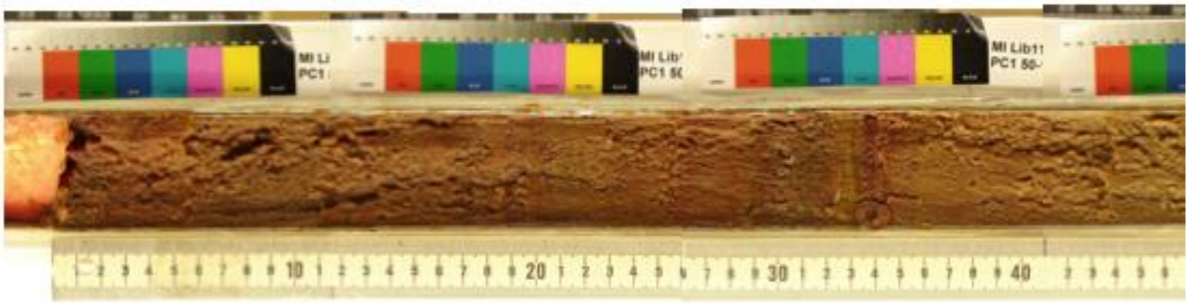

Lib 6 PC

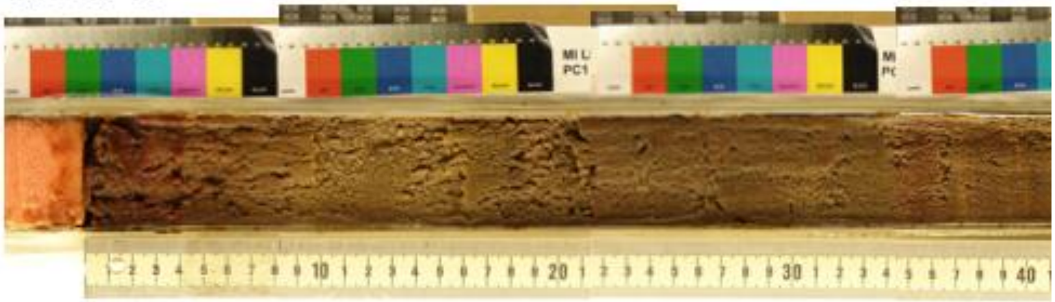

Lib 11 PC

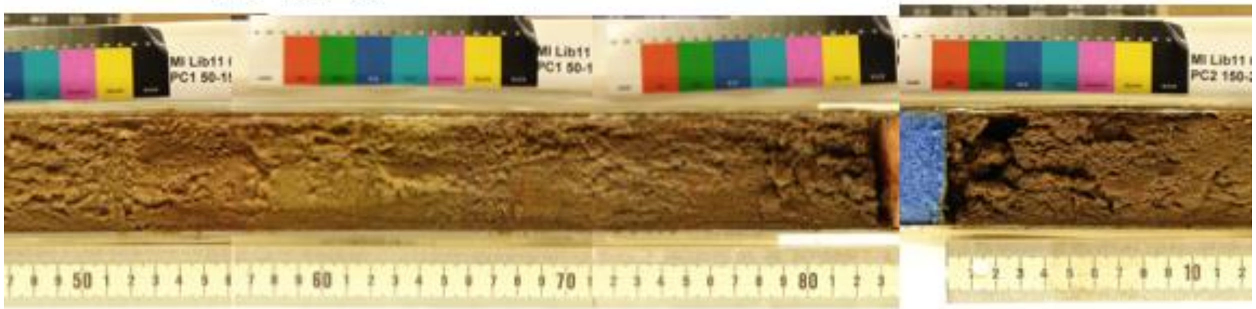

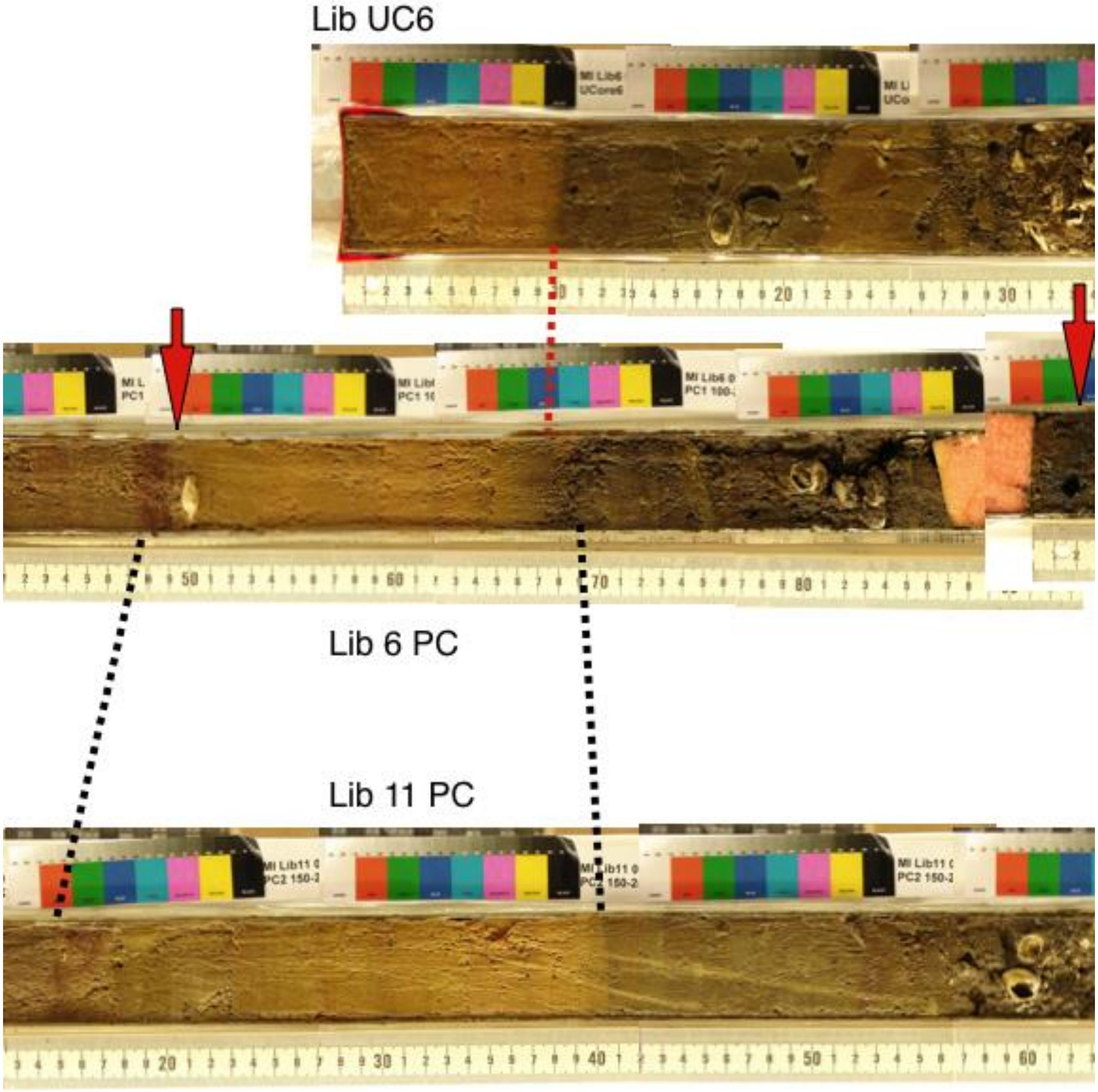

Lib UC6
Lib 6 PC
Lib 11 PC

Lib UC8

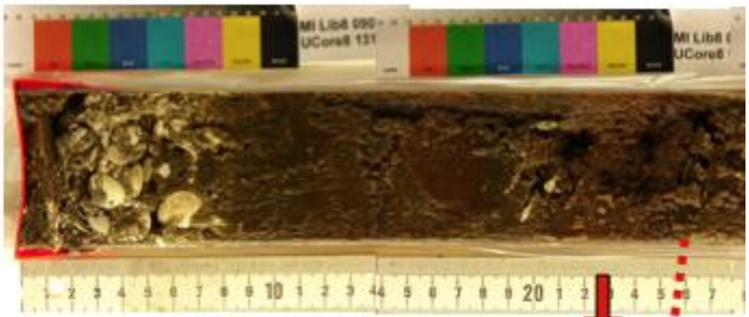

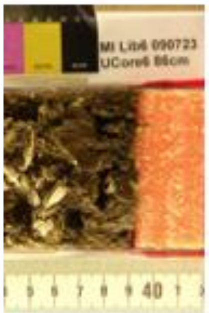

Lib UC5

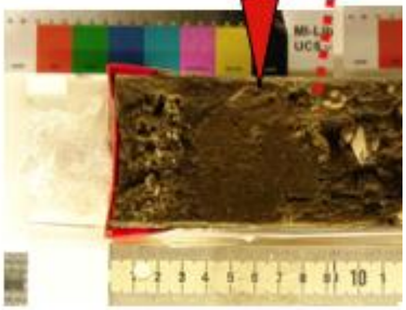

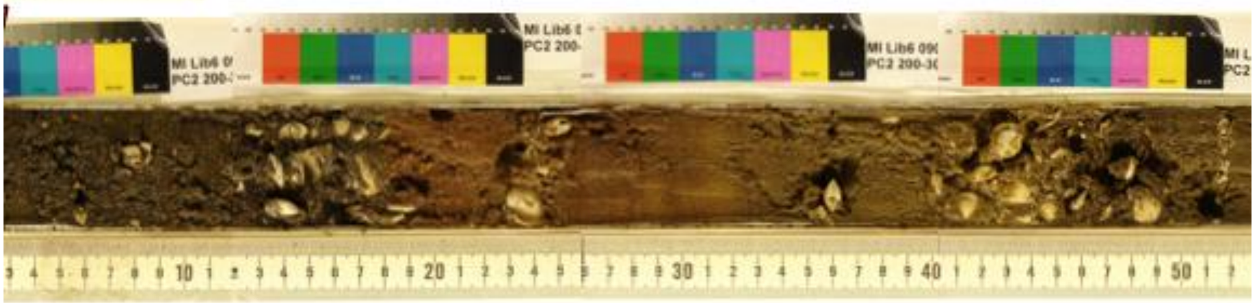

Lib 6 PC

Lib 11 PC

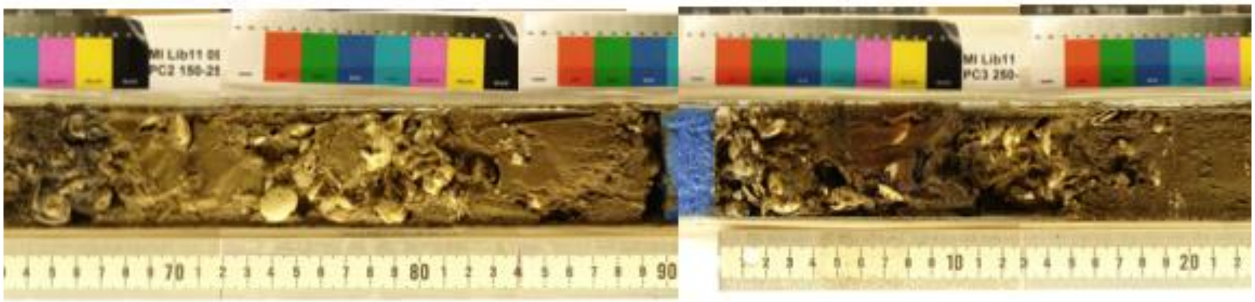

Lib UC5 (2nd from top -- no space to directly label)

Lib UC8

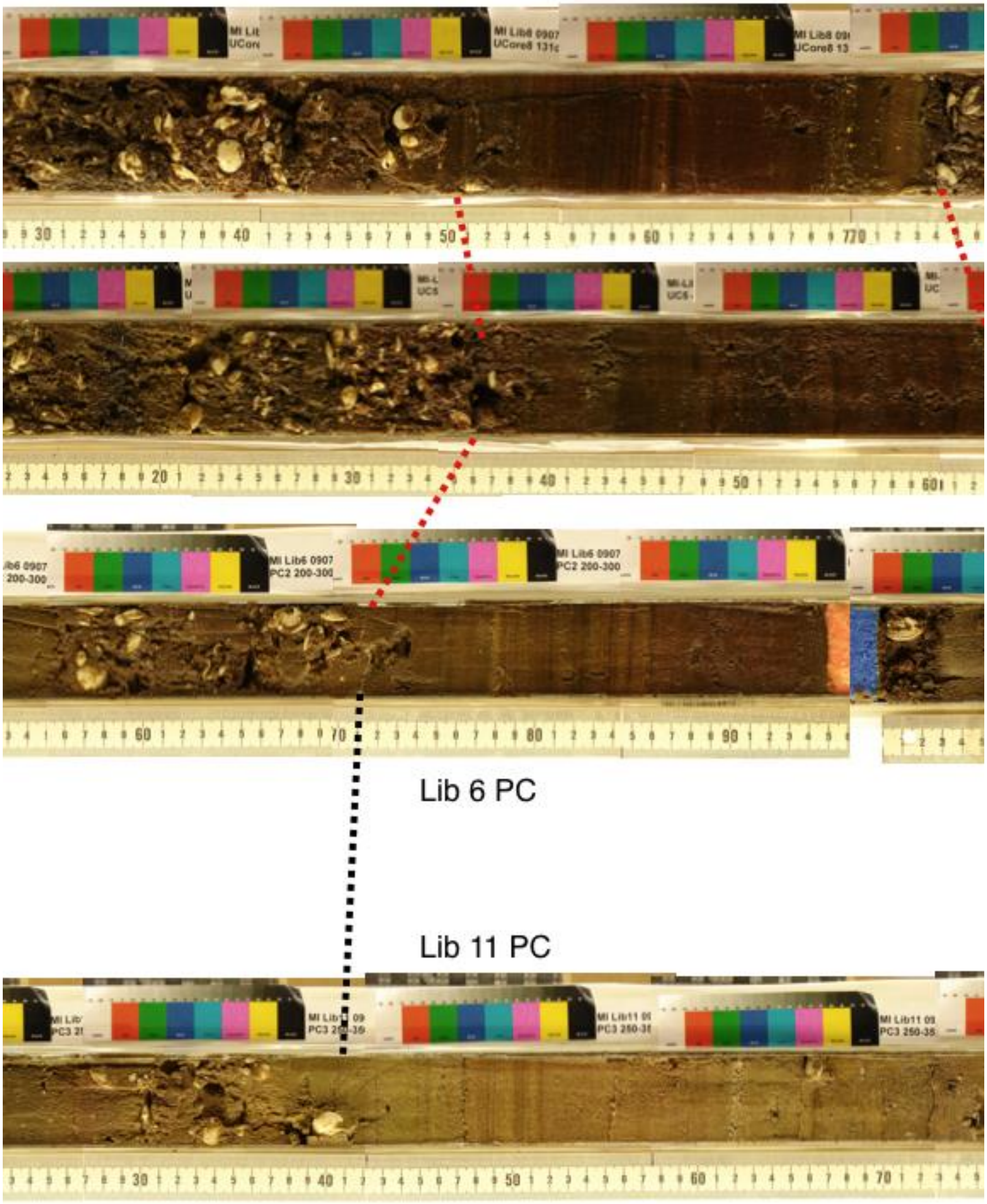

Lib 6 PC

Lib 11 PC

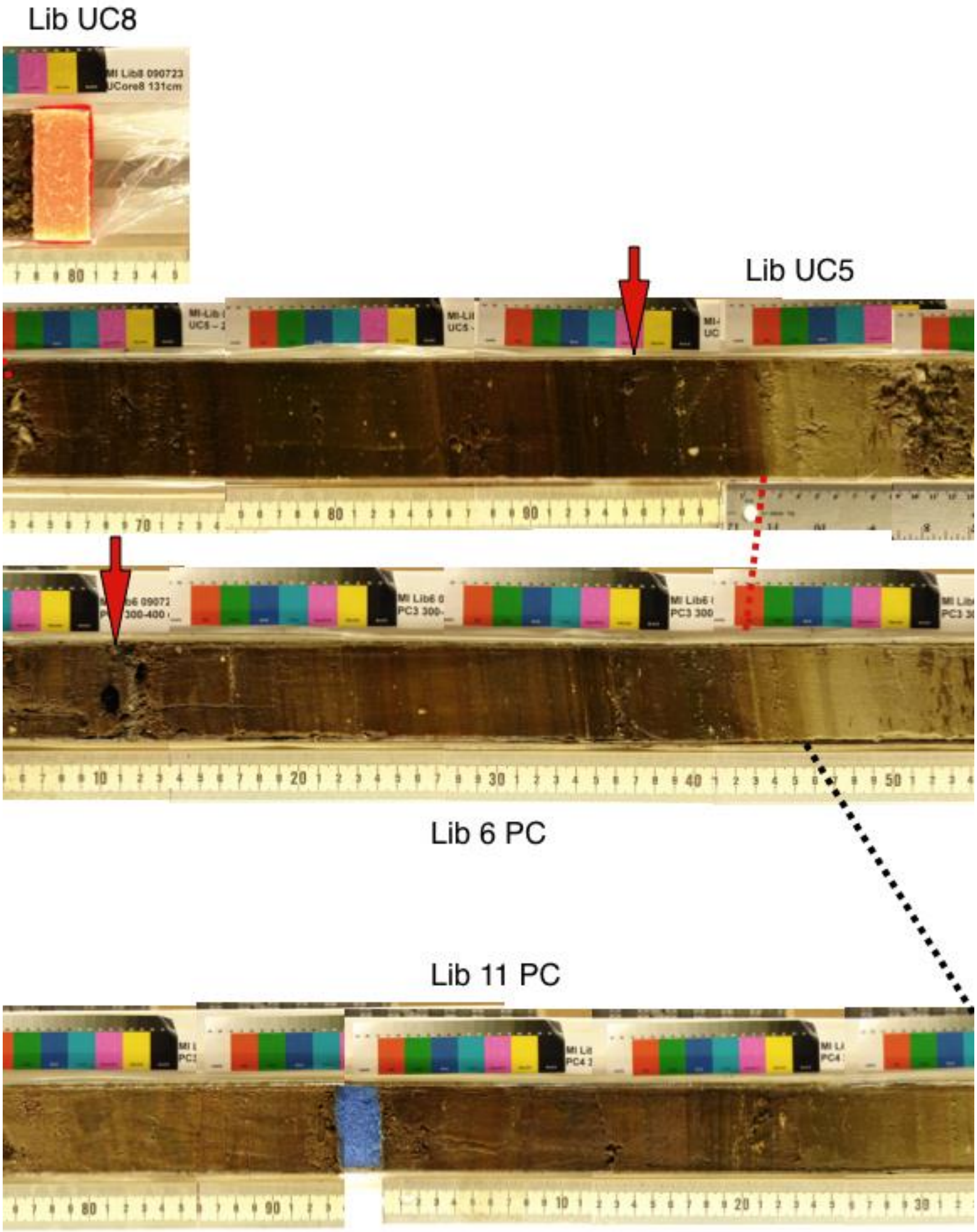
Lib UC8
MI Lib8 090723
UCore8 131cm
Lib UC5
Lib 6 PC
Lib 11 PC

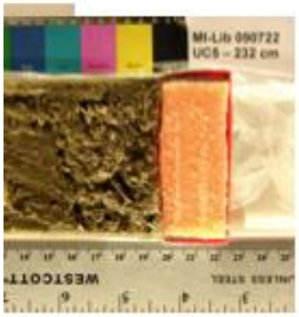

Lib UC5

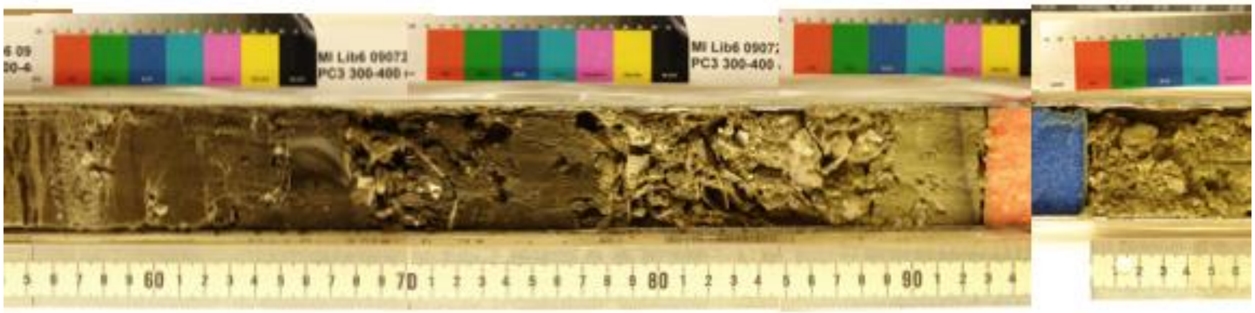

Lib 6 PC

Lib 11 PC

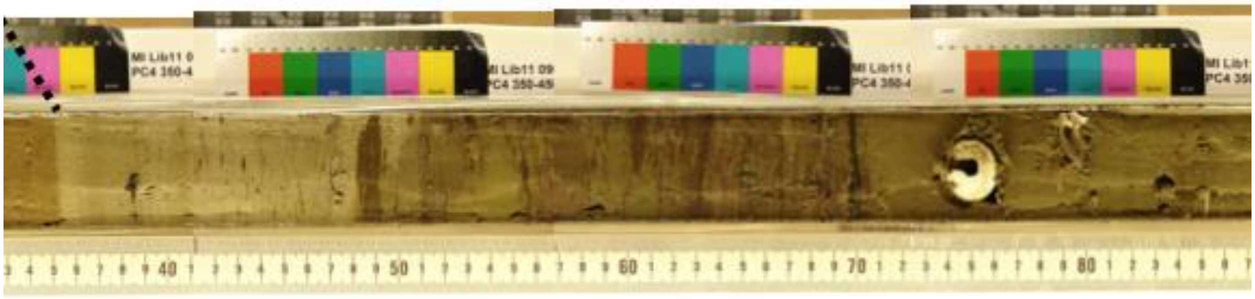

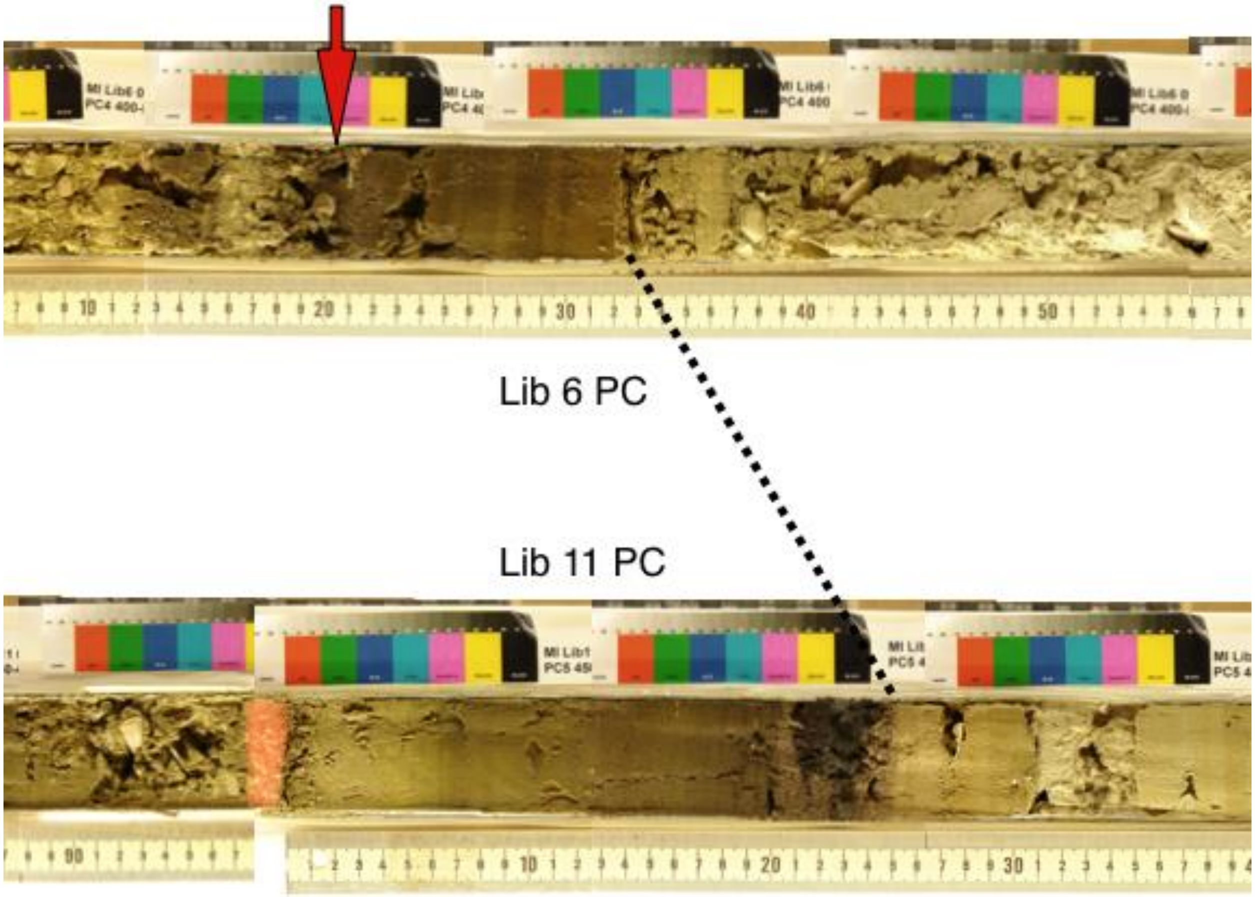

Lib 6 PC
Lib 11 PC

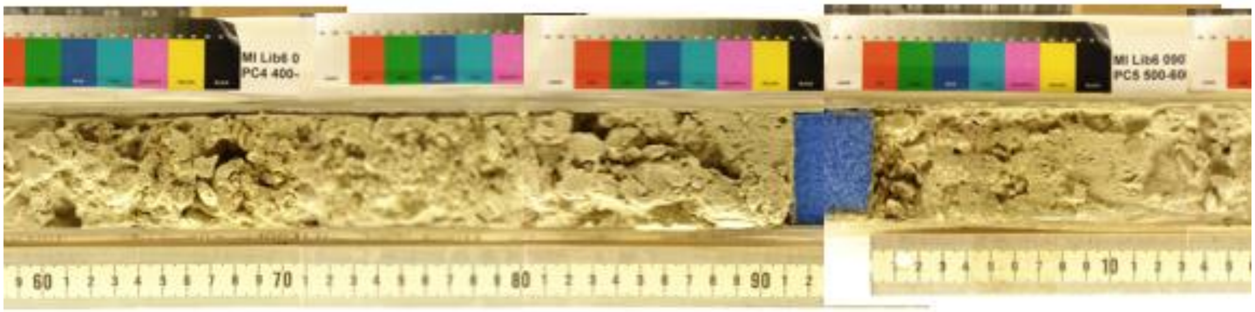

Lib 6 PC

Lib 11 PC

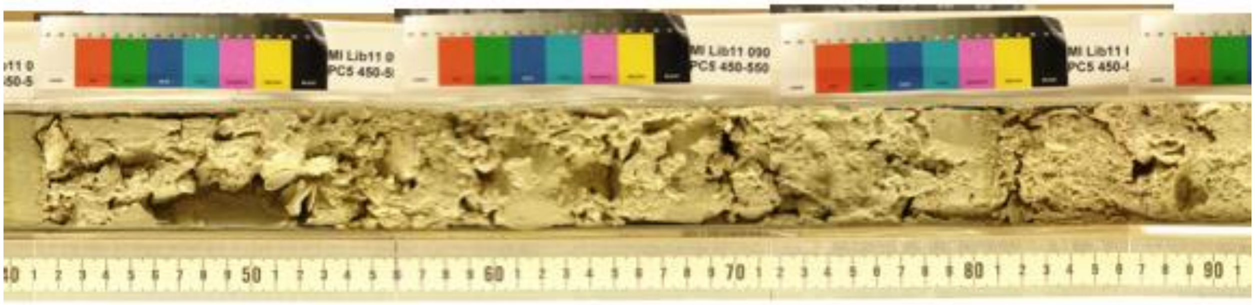

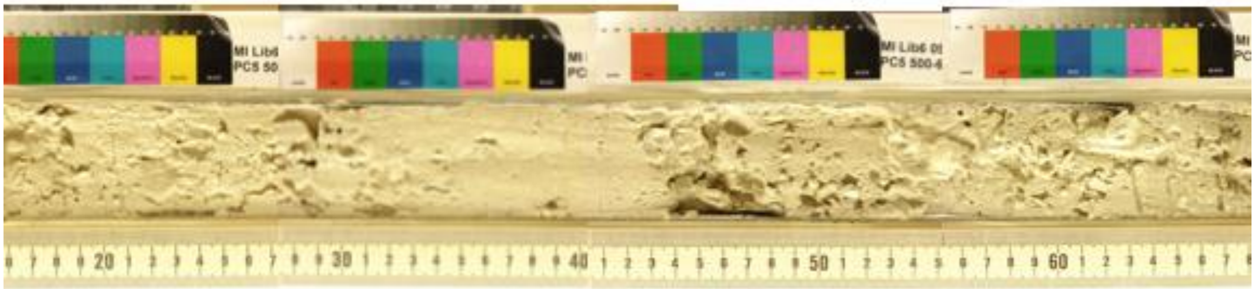

Lib 6 PC

Lib 11 PC

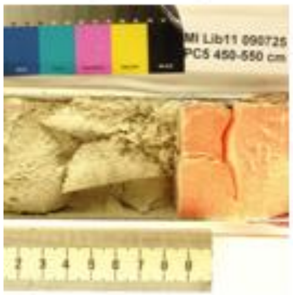

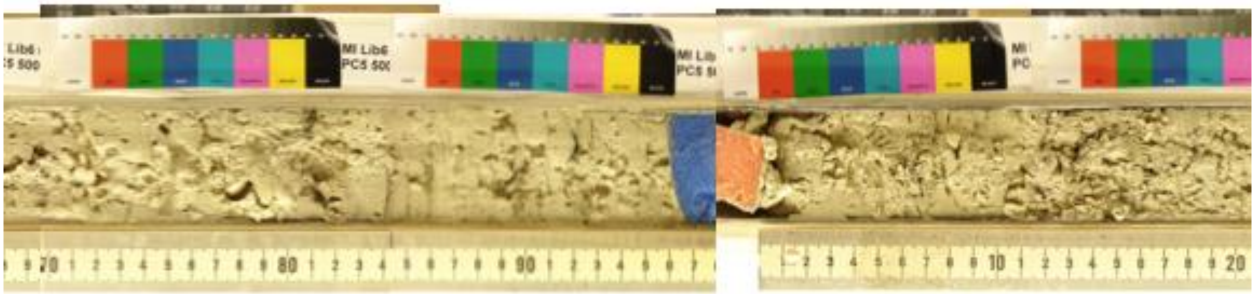

Lib 6 PC

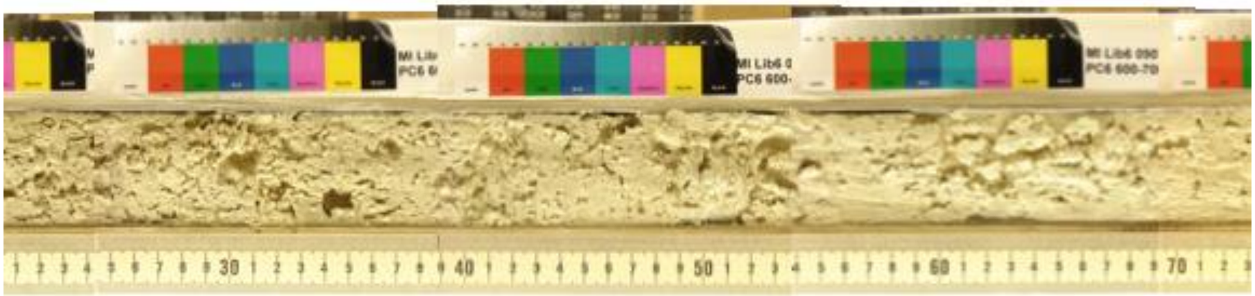

Lib 6 PC

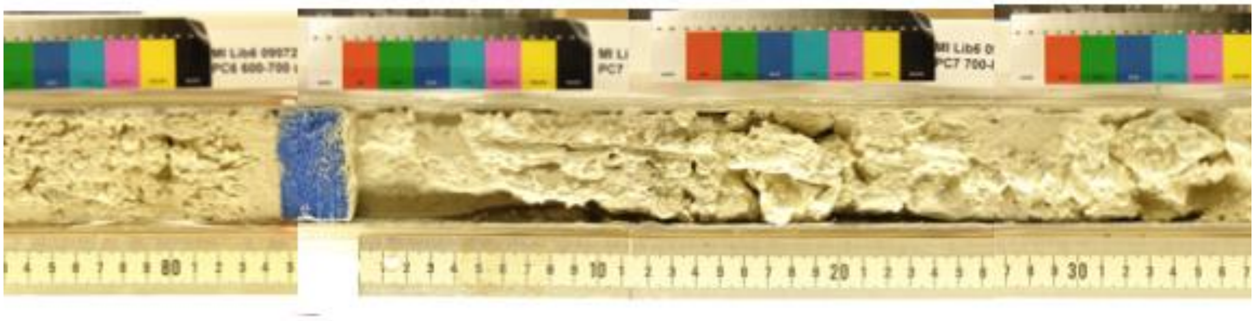

Lib 6 PC

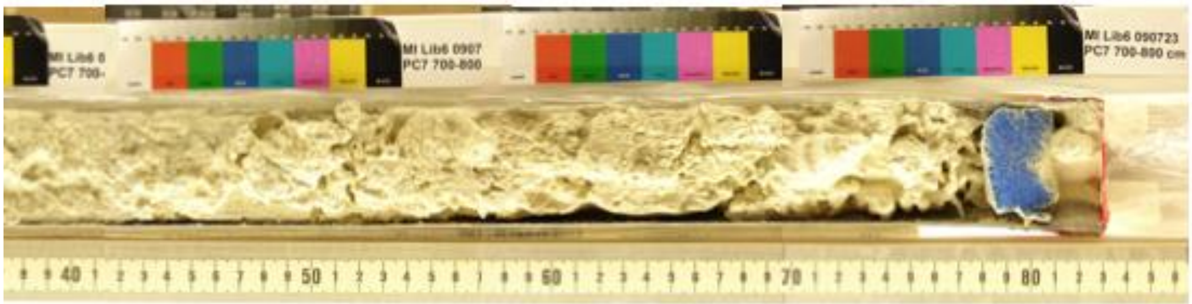

MI Lib6 0
PC7 700-
MI Lib6 0907
PC7 700-800
MI Lib6 090723
PC7 700-800 cm
40
50
60
70
80

Lib 6 PC

Lib Pond Vegetation Survey

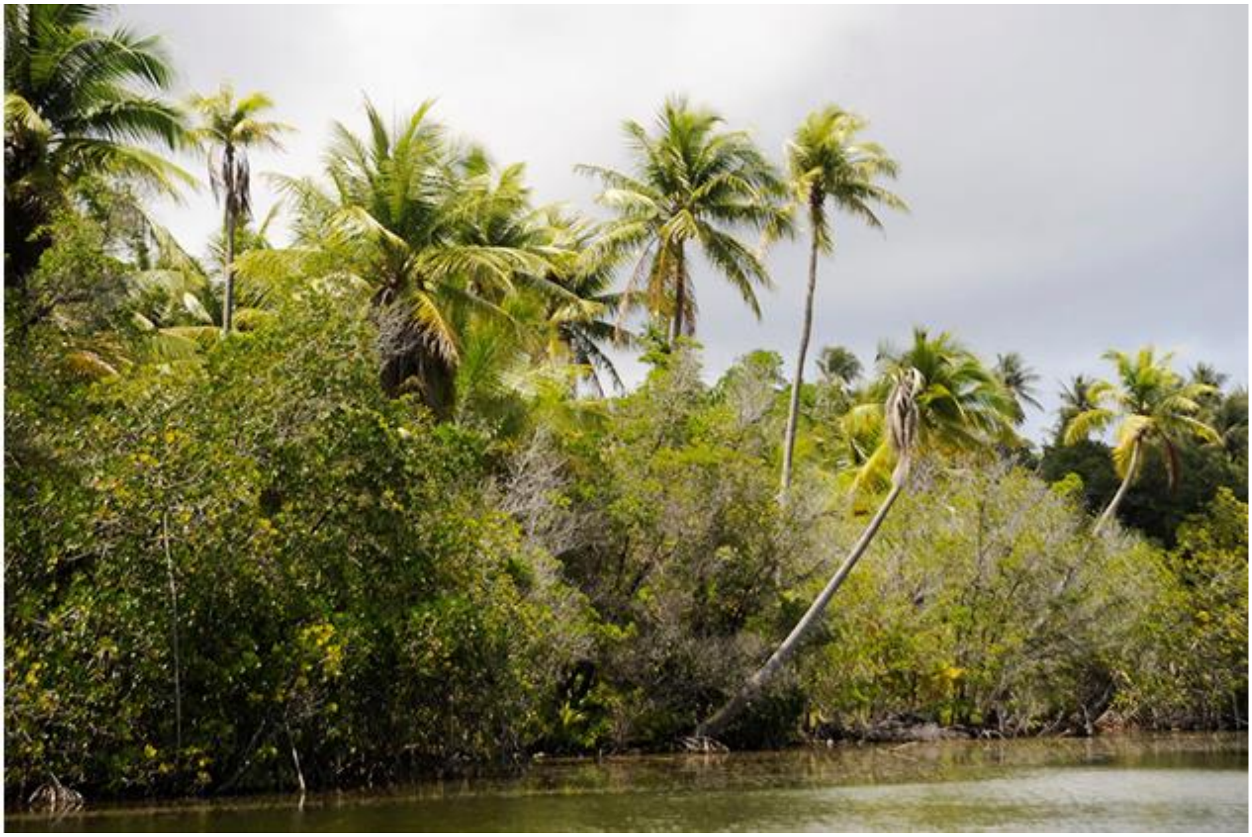

Figure S37. Example of Lib Vegetation along Shoreline.

Lib Pond Vegetative Sample Examples

12 different vegetation species were noted and sampled where possible along the north end of Lib Pond. Below are pictures of several selected species. Here we show photographs of 11 of them (the 12th is not pictured). Vegetative sample #5 was unable to be collected due to the height of the palm tree. Collection was done along the northernmost lake shoreline, on July 22nd 2009. For more information about the vegetation samples collected, contact the author.

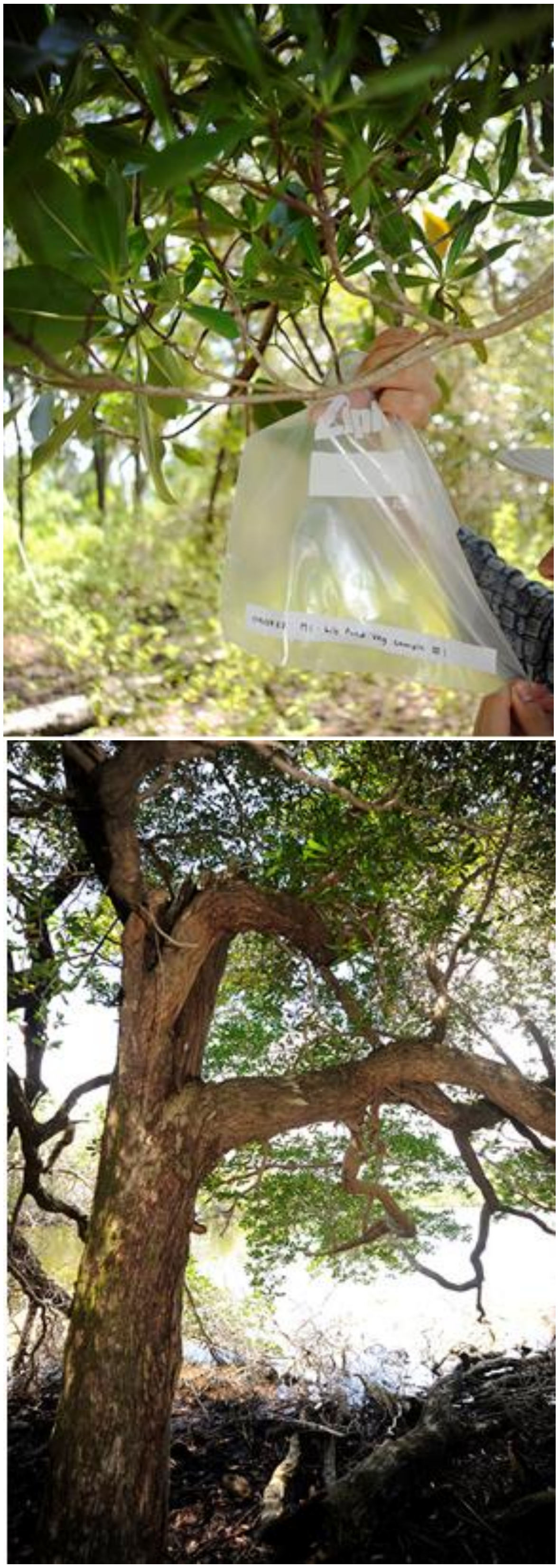

Figure S38.

Lib Pond Vegetative Sample 1

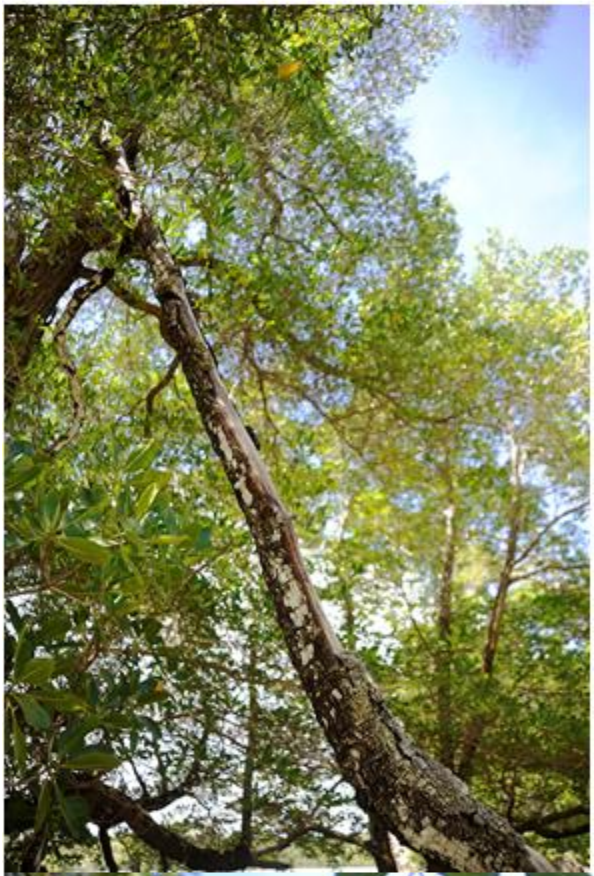

Figure S39.

Lib Pond Vegetative Sample 2

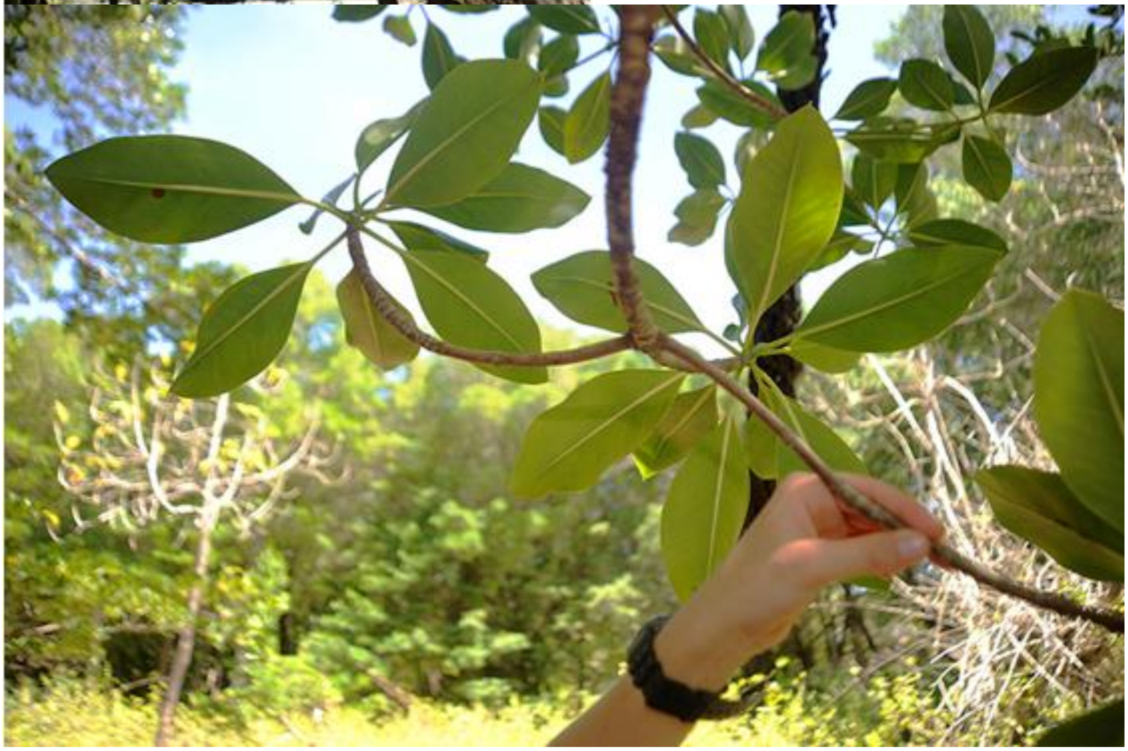

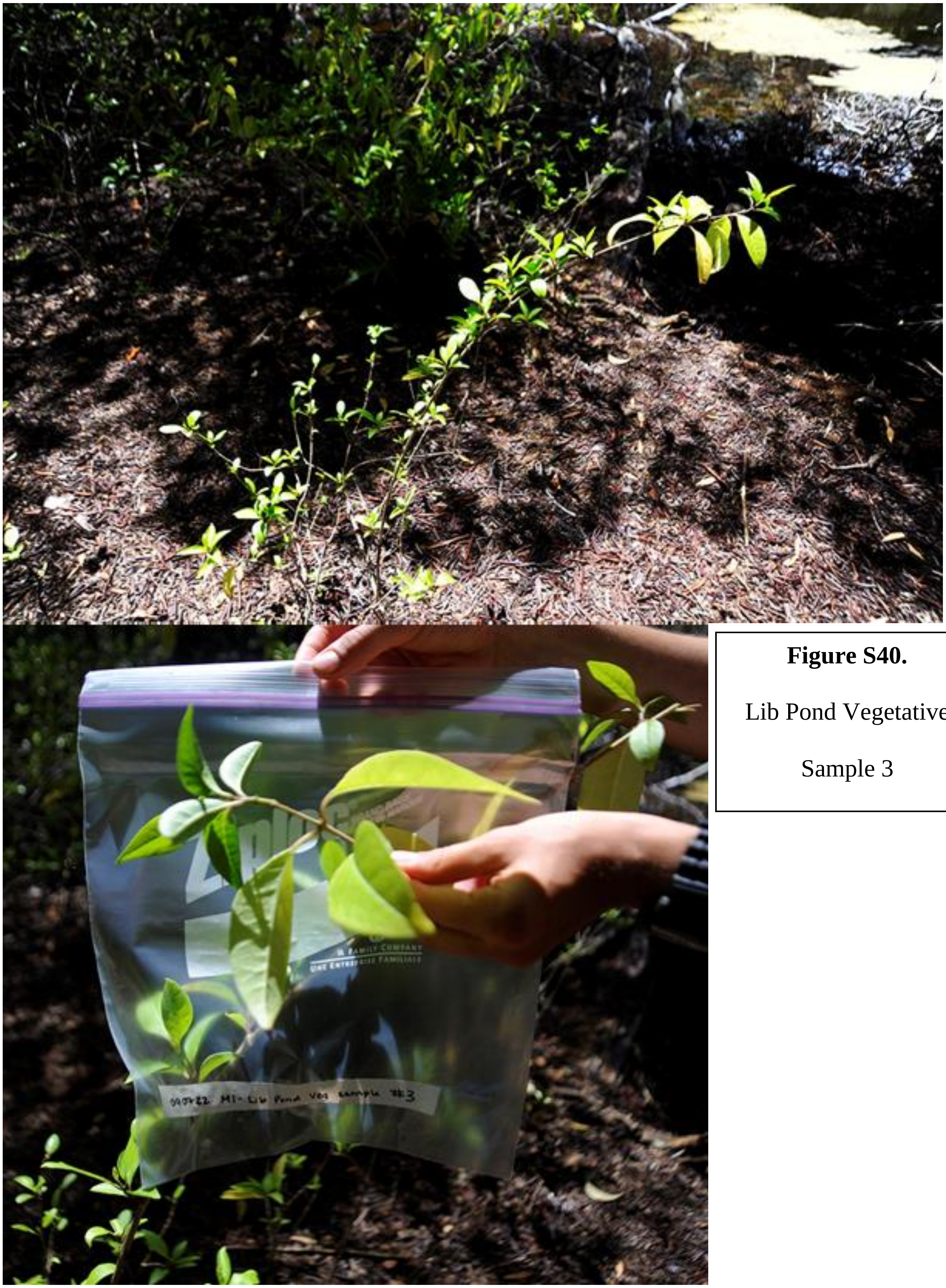

Figure S40.

Lib Pond Vegetative

Sample 3

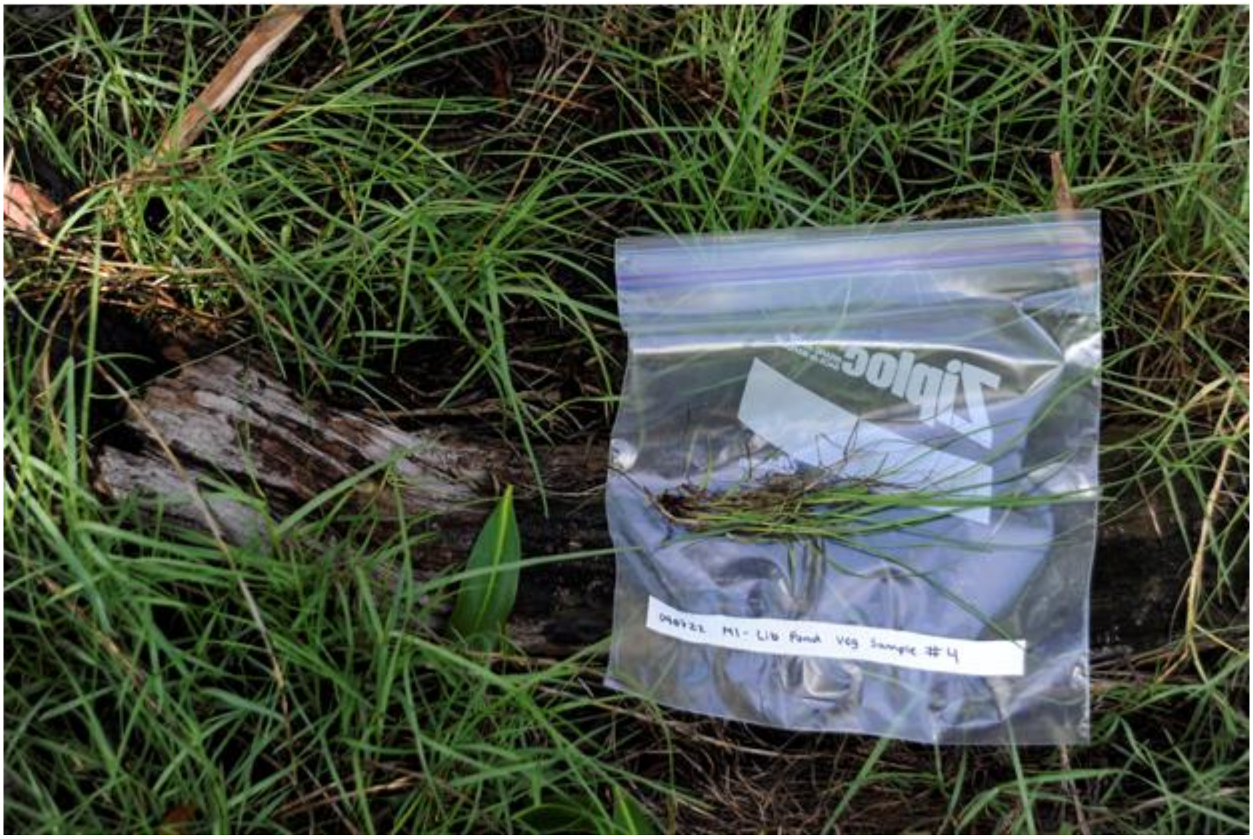

Figure S41. Lib Pond Vegetative Sample 4

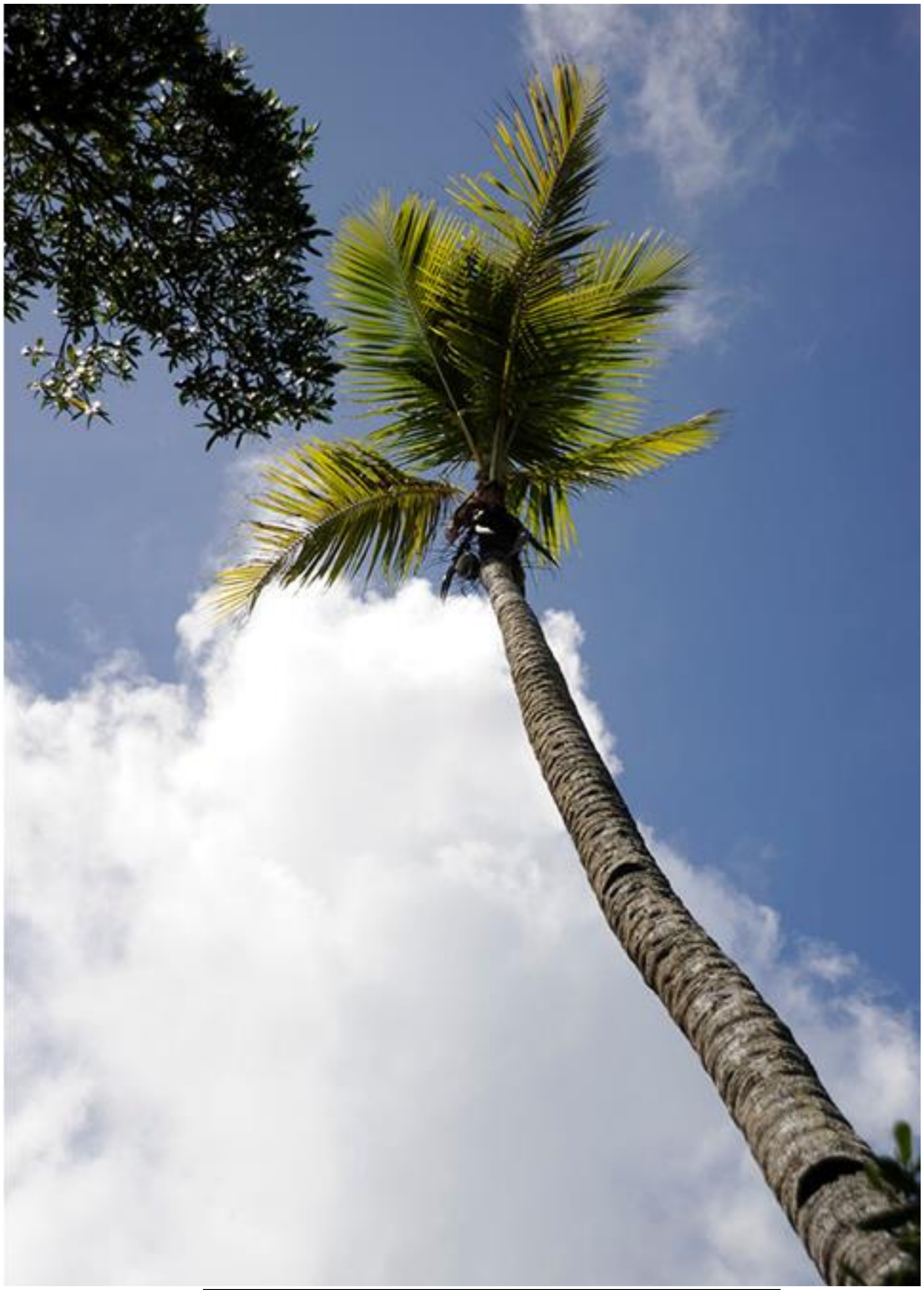

Figure S42. Lib Pond Vegetative Sample 5

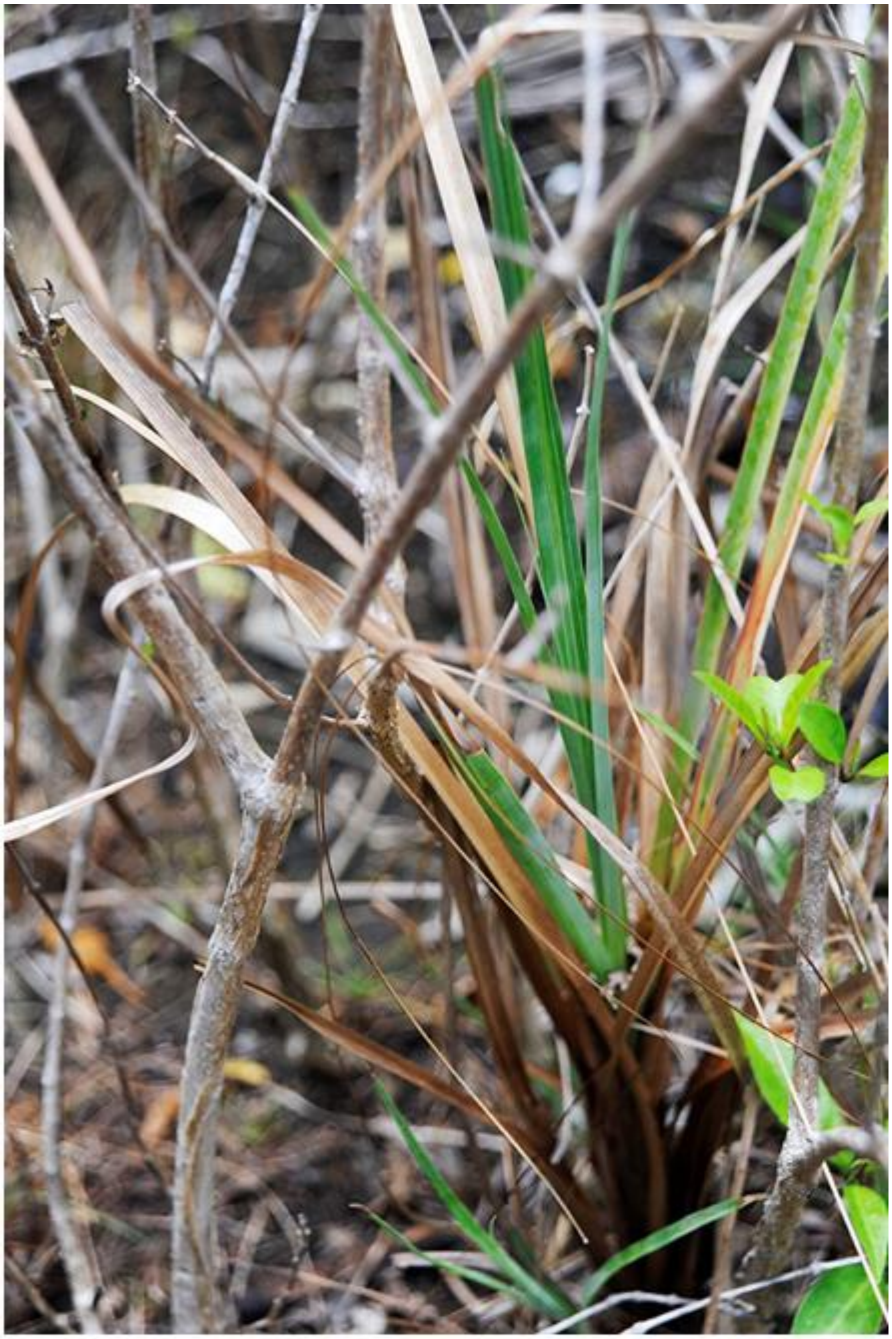

Figure S43. Lib Pond Vegetative Sample 6

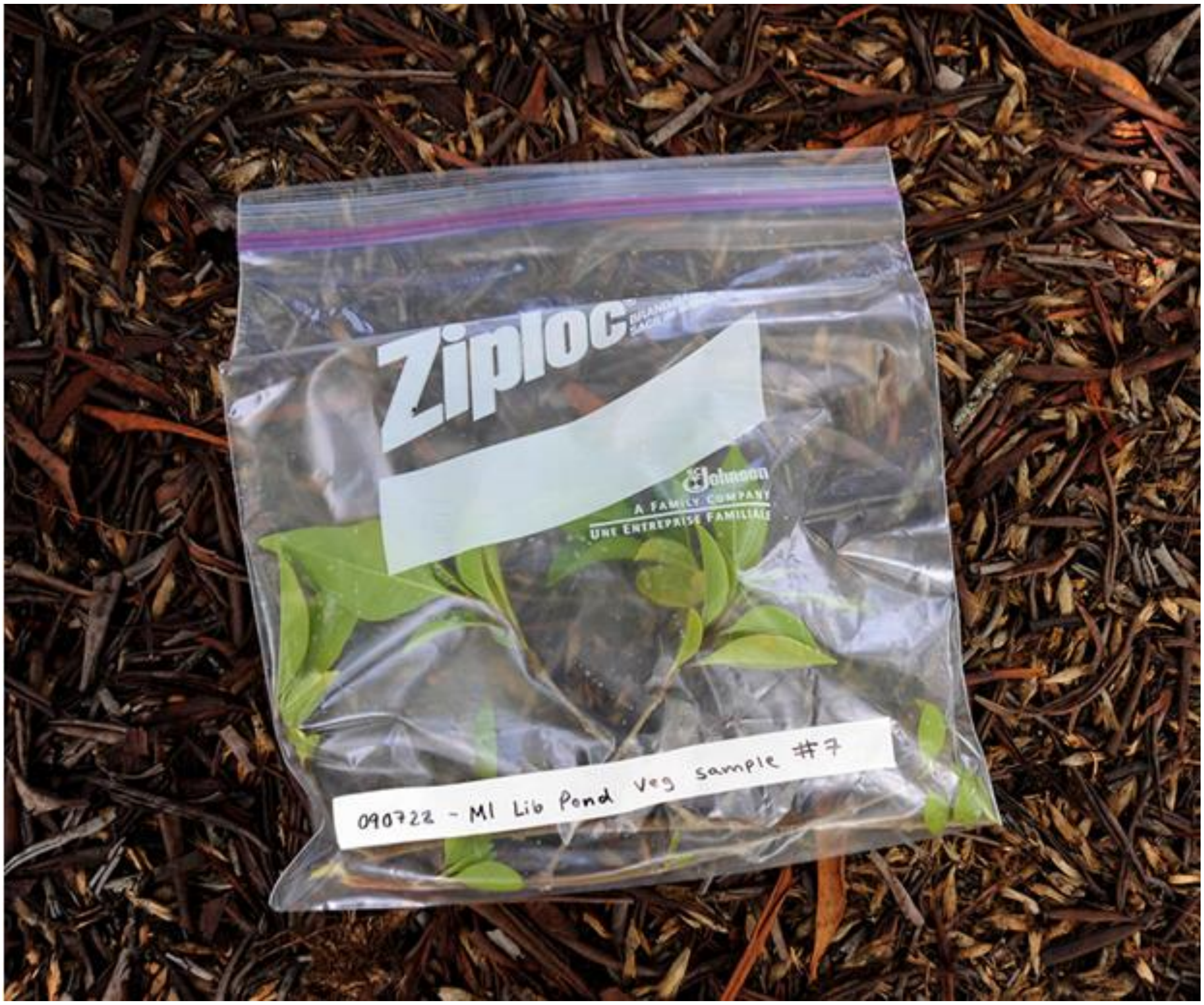

Figure S44. Lib Pond Vegetative Sample 7

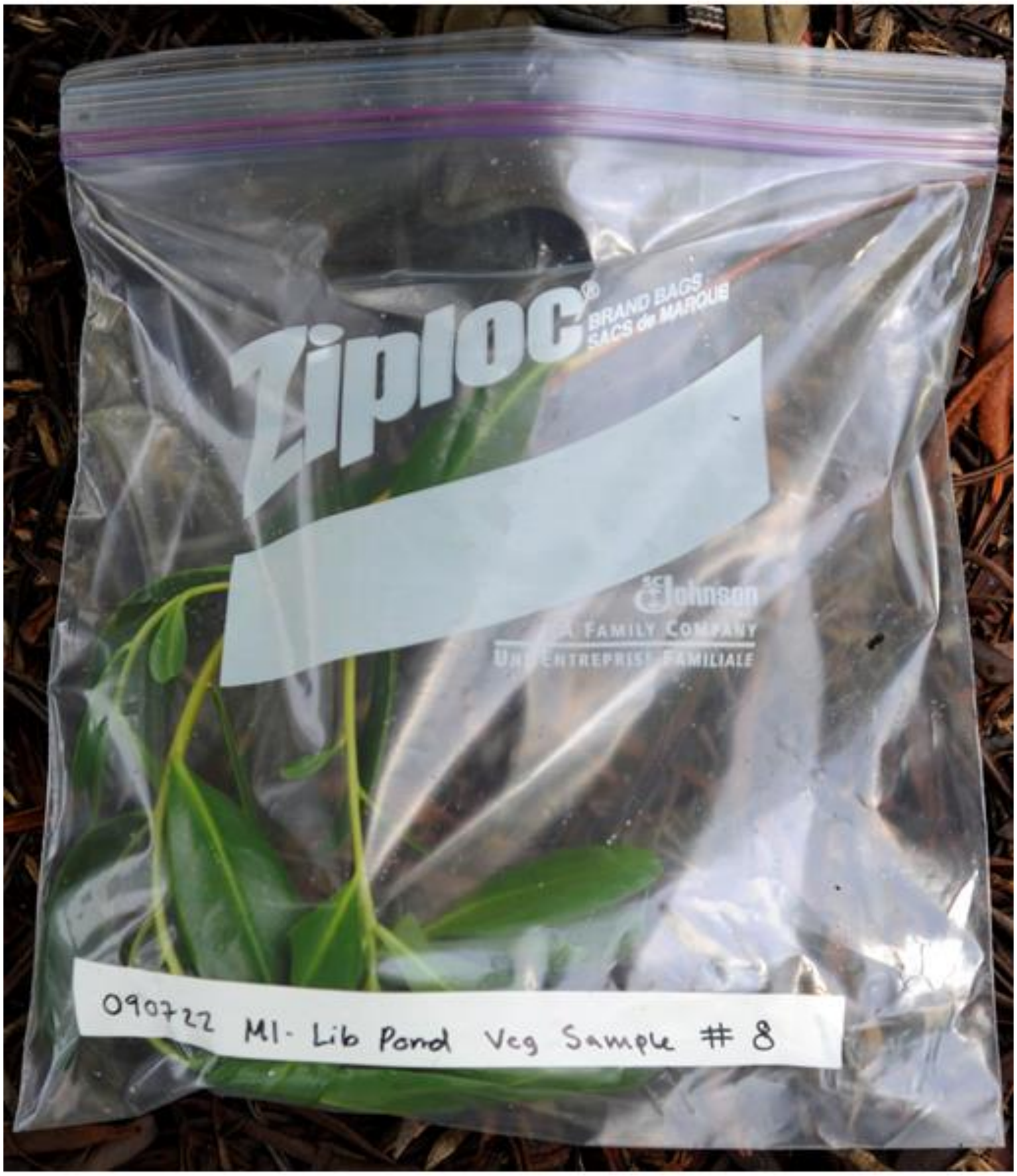

Figure S45. Lib Pond Vegetative Sample 8

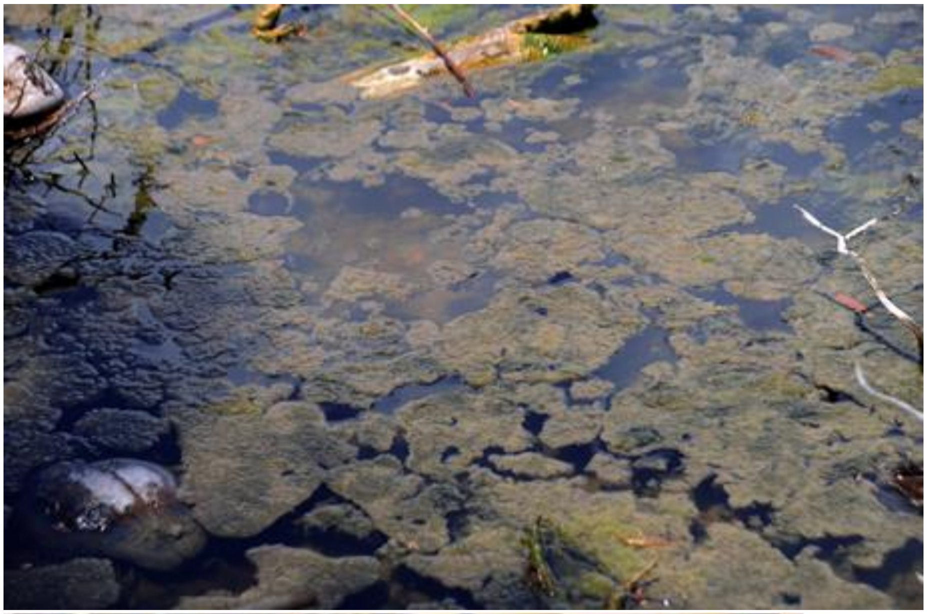

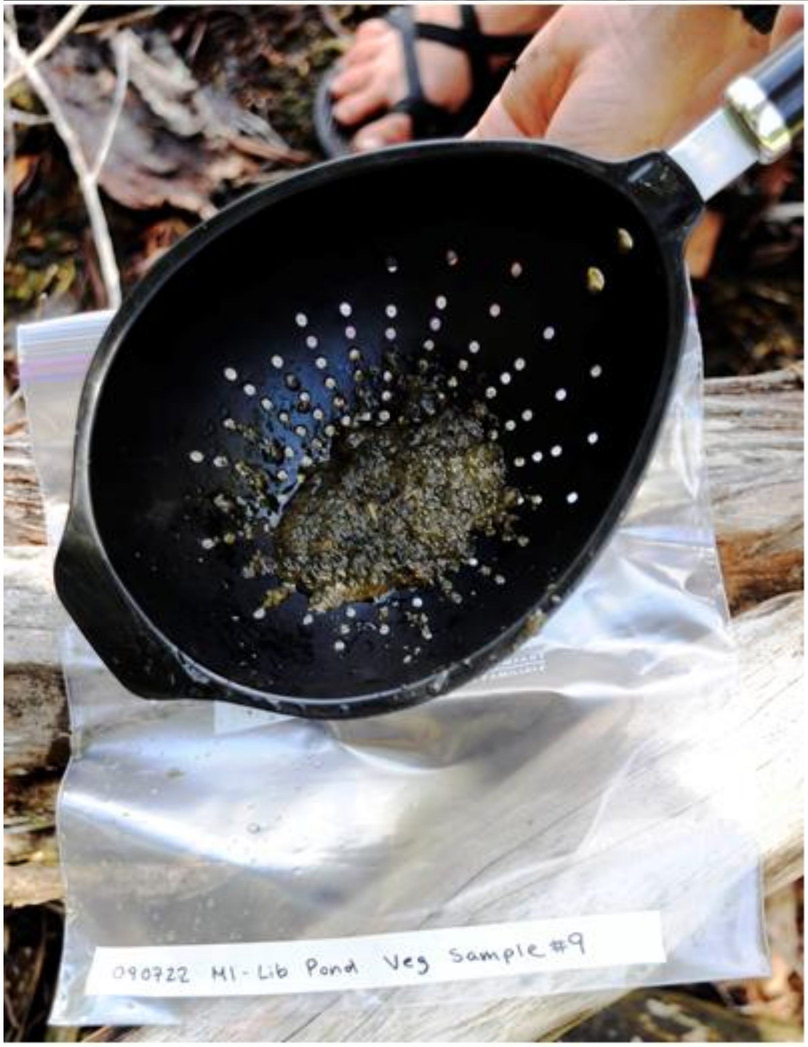

Figure S46.

Lib Pond Vegetative

Sample 9

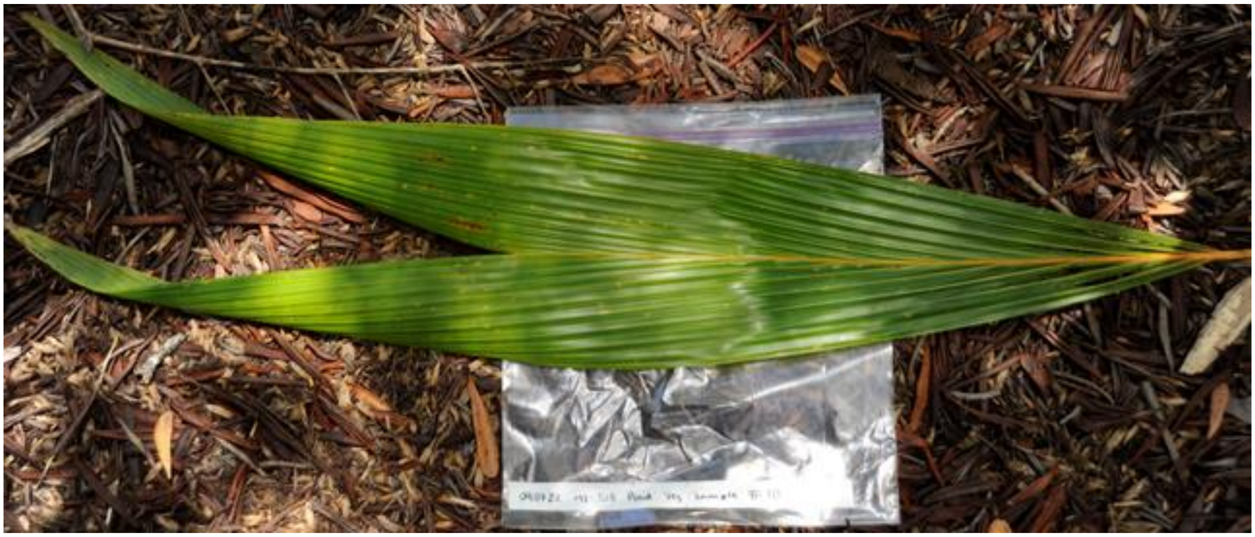

Figure S47. Lib Pond Vegetative Sample 10

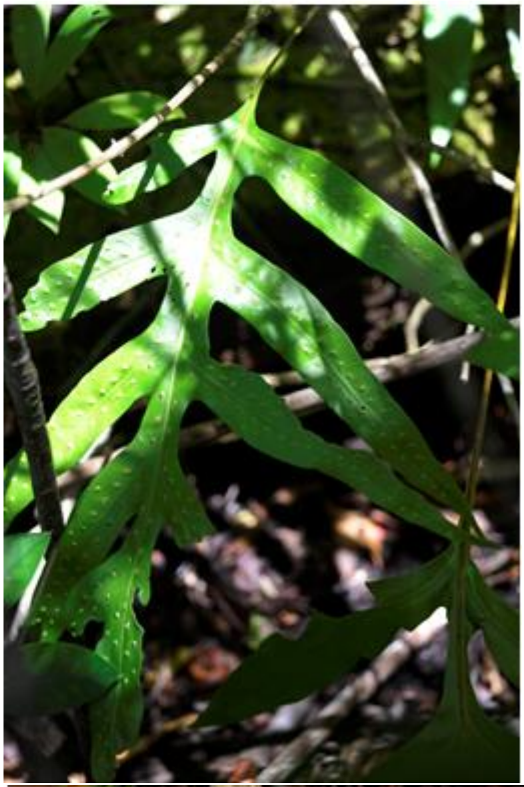

Figure S48.

Lib Pond Vegetative Sample 11

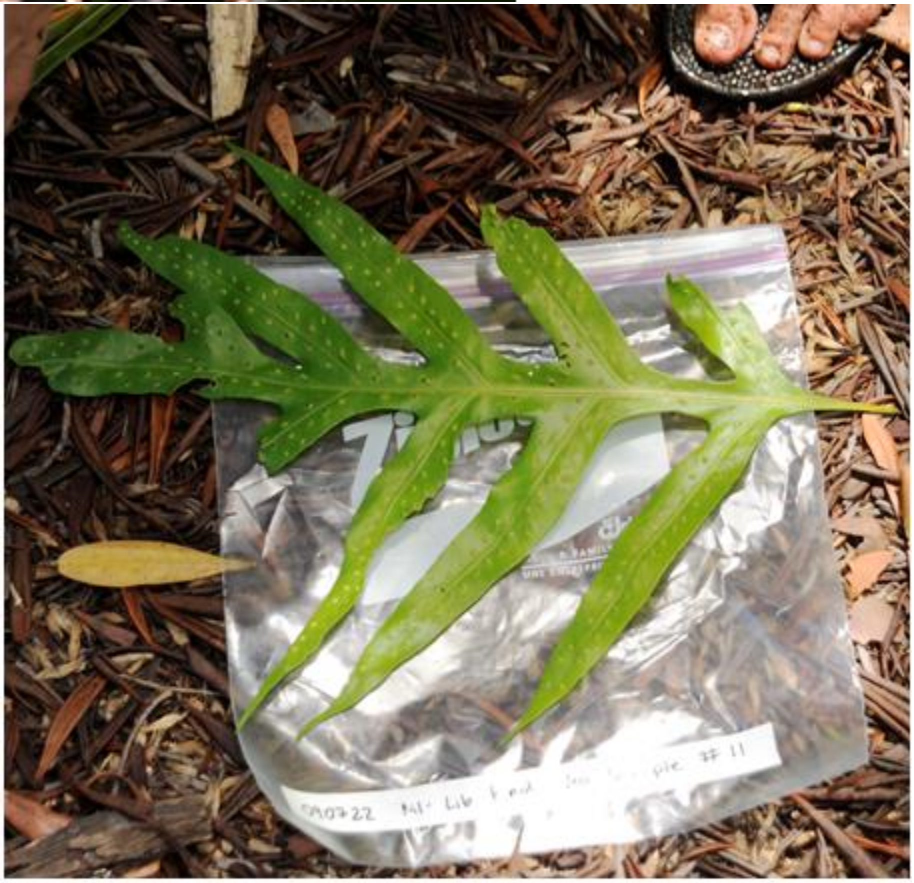

Conventional Bathymetry Background

The conventional approach to bathymetry is a statistical fitting of a smooth surface through a set of depth points. Numerous depth data are collected in a grid or by crisscrossing transects [9]. A bathymetric map is then created from statistical techniques commonly available in GIS software such as kriging [10] or laplacian smoothing [11]. However, these statistical methods only work if sufficient depth data exists because they do not assume any knowledge about the lake other than the depth values and locations.

In this case, field conditions prevented the collection of a sufficient large data set (and depth transects) to use those conventional methods. A standard interpolation technique by itself produces a bathymetric map with unrealistic features: knife-edge ridges and sharp angles which are inconsistent with field measurements and experiences with similar types of lakes.

A prior methodology measures the central deep point of a body of water and just several other points in all four directions, orthogonal to each adjacent direction, forming a cross [12]. “Synthetic” radial transects are modeled upon the observed transects to create a smoothed transect with a realistic lake bottom (e.g. no rough or jagged edges). However, Lib Pond contains smaller features and is more irregular than the wetland basins and we did not collect perpendicular linear transect data (a prerequisite to creating radial transects). Additionally, the deepest part of Lib Pond is unknown and the required laser surveying equipment was unavailable.

Other conventional methods that do not employ the above statistical techniques include multispectral satellite imagery which produces a bathymetric map with centimeter-resolution [13] [14] and radial transects [12]. Yet satellite images of Lib Pond at multiple frequencies are not available.

Boundary Slope Fitting Methodology

Boundary slope fitting creates a model of near-shore lake regions using information nearby regions with similar characteristics and can be used for any shallow body of water. It is considerably more cost effective than other conventional bathymetric methods in terms of equipment and in terms of the number of required depth measurements. Original bottom data is sufficient to determine lake bathymetry if supplemented with data from the lake edges. Hence, boundary slope fitting adds modeled data points around the lake perimeter, which in turn are extrapolated from known nearby bottom contours. With the addition of these modeled shore data points, the new profile improves the bathymetric map by eliminating implausible features of an interpolation, improving the agreement of the bathymetry with both field observations and the satellite image.

The transects yield slopes from the middle of the lake to the lake edge and are consistent with each other, taking into account the different angles of the transects in relation to each other. Thus lake regions without depth data can be indirectly determined from adjacent, known lake slopes. Field observations and unrecorded depth data were used to make the assumption that the slope characteristics are relatively homogenous in a given section where no direct depth data was taken: similar shore types and bottom conditions imply similar slope characteristics.

We used exponential functions since polynomials are not flat at the center of the lake as you extend from the shoreline (most lake bottoms, including Lib, are asymptotically flat).

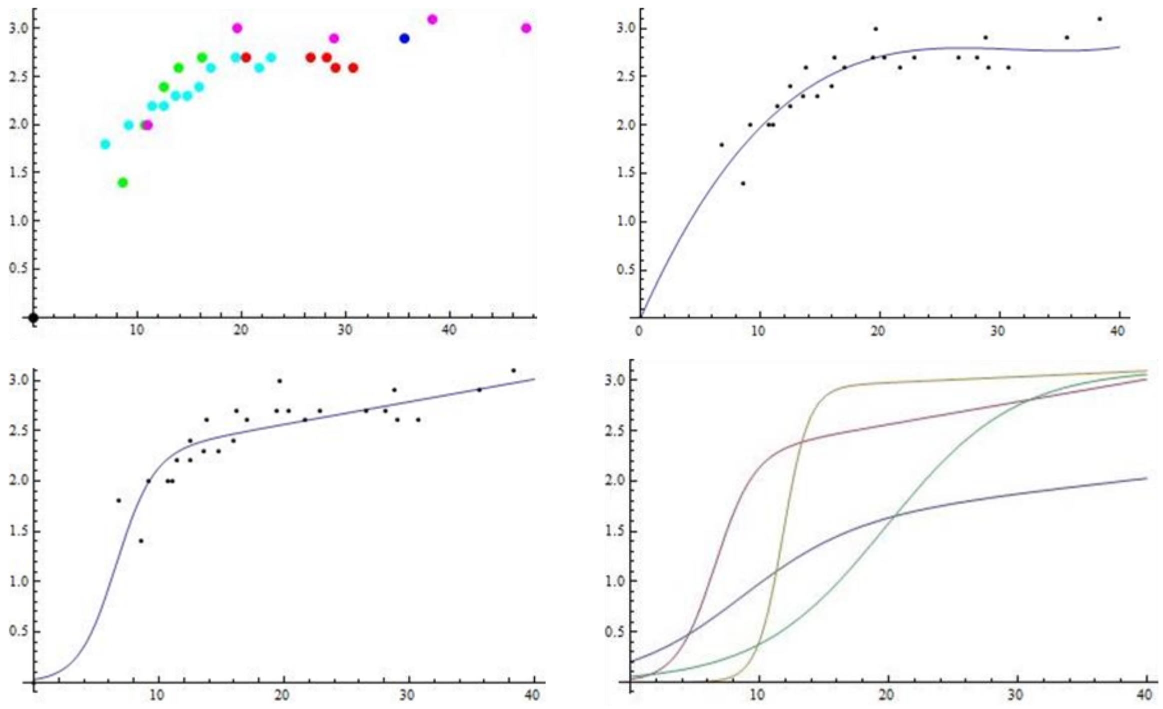

Figure S49. Top left: Example of a linear (directionally speaking) transect, represented by a series of individual GPS coordinates with depths. Top right: The transect fit to a polynomial function, which was rejected. Bottom left: The transect was fit to an exponential with an R^2 value of >0.995. Bottom right: All of the transects fit to exponential functions. The variation in the slope is a result of different shoreline and bottom profile types present in the pond.

Boundary Slope Fitting Transects

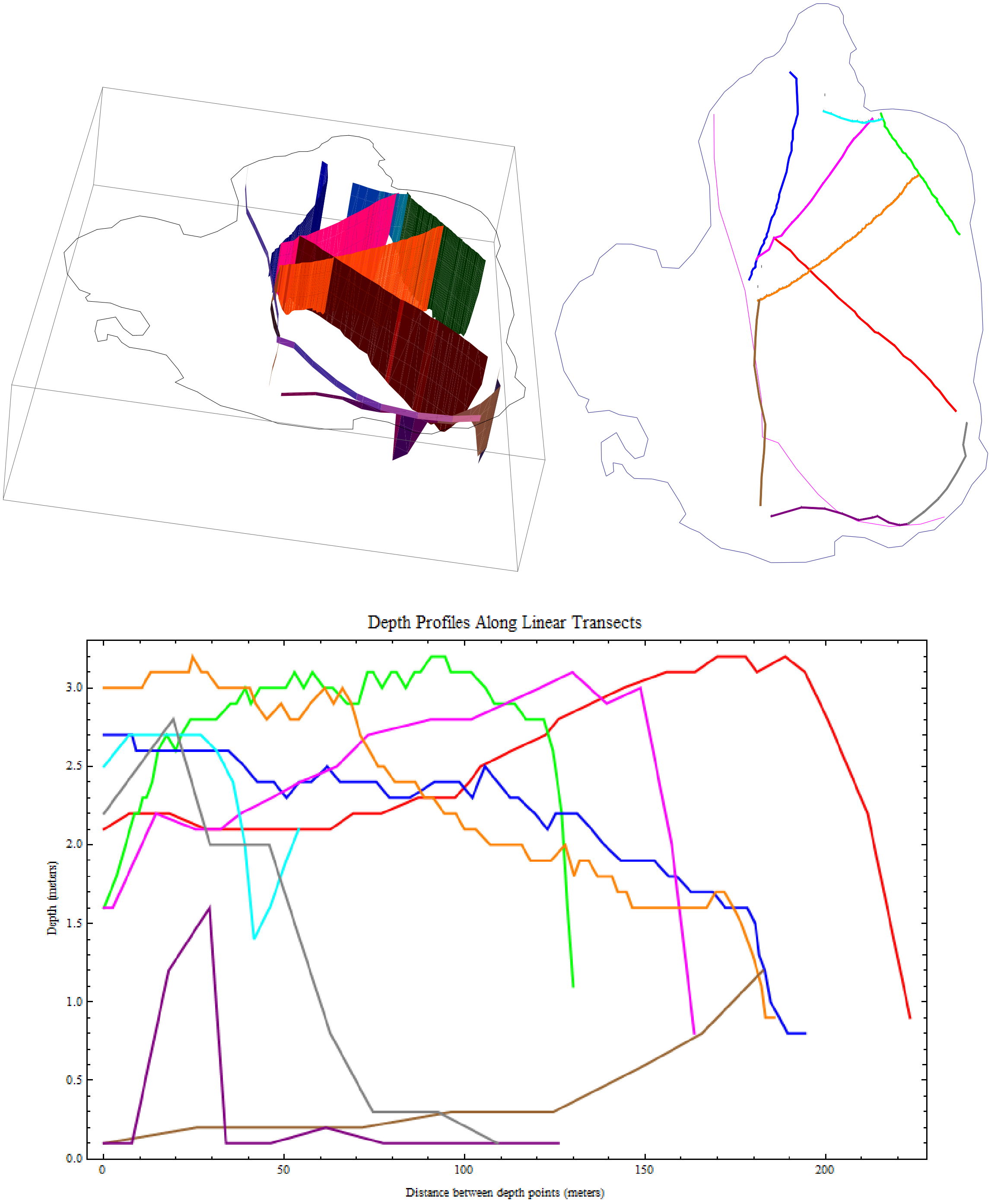

Figure S50. (Above) The pond transects, shown in 3D on the left and in 2D on the right. Each color represents a different transect. The transects were broken up so that they would be in the

same direction; that is to say, roughly linear. On the right, the thin purple line is the outline of the shallow region. Several individual points that did not compose transects are also visible. On the right hand side, North is up. (Below) The depth profiles along the transects.

Boundary Slope Fitting Edge Type Matches

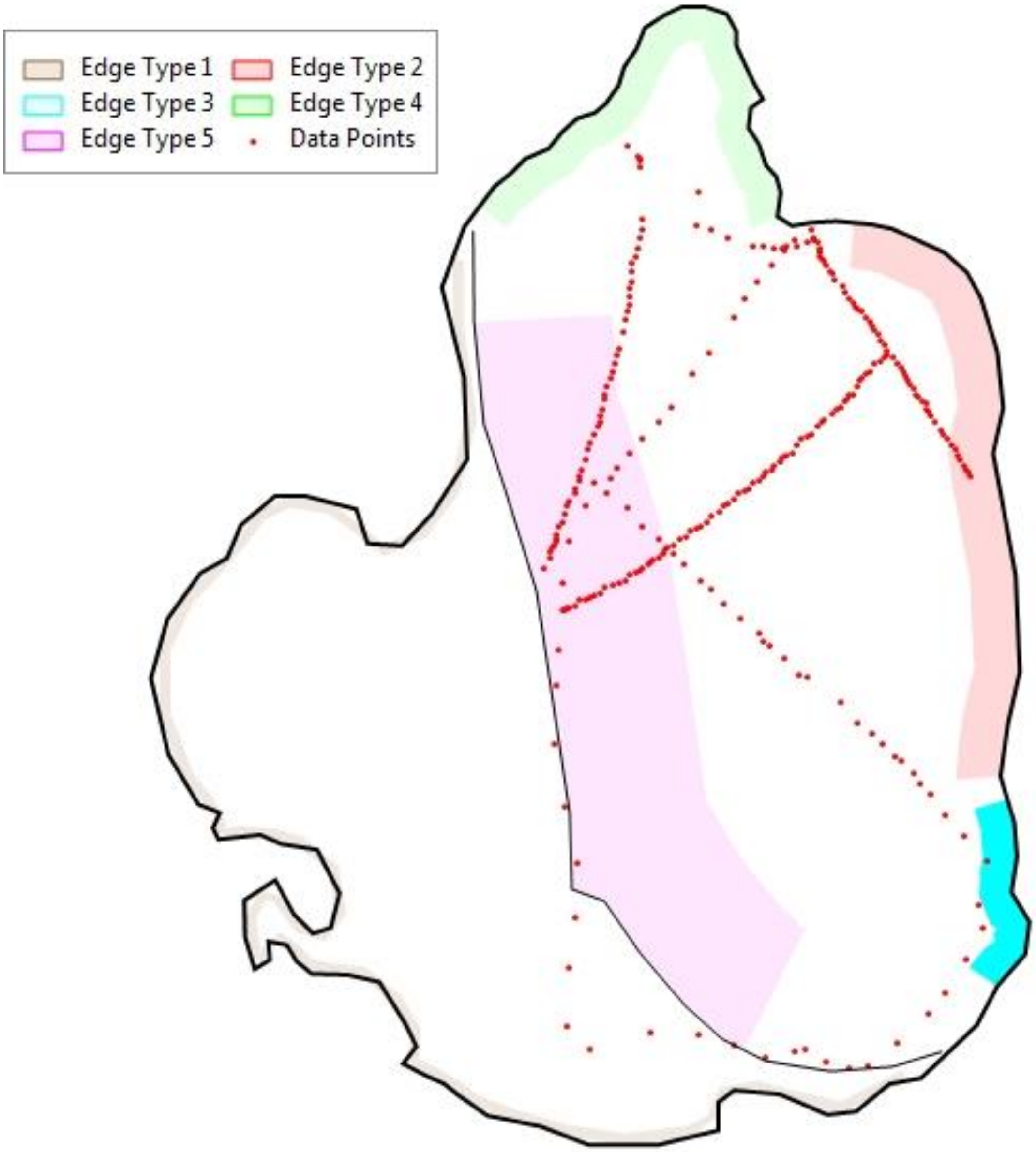

Figure S51. An outline of Lib Pond with the boundary slope fitting edge types, based on depths. The unnavigable shallow floodplain is located to the bottom left and marked by a black line. Edge types indicate similar slopes.

Created Contours from Boundary Slope Fitting, Before and After

For boundary slope fitting contours of Lib Pond shown against the satellite image of the pond, please contact the corresponding author. The channel on the lower left is accurately reflected in the bathymetry to the right of the channel opening. These contours are compared against contours from a standard interpolation of the original data set.

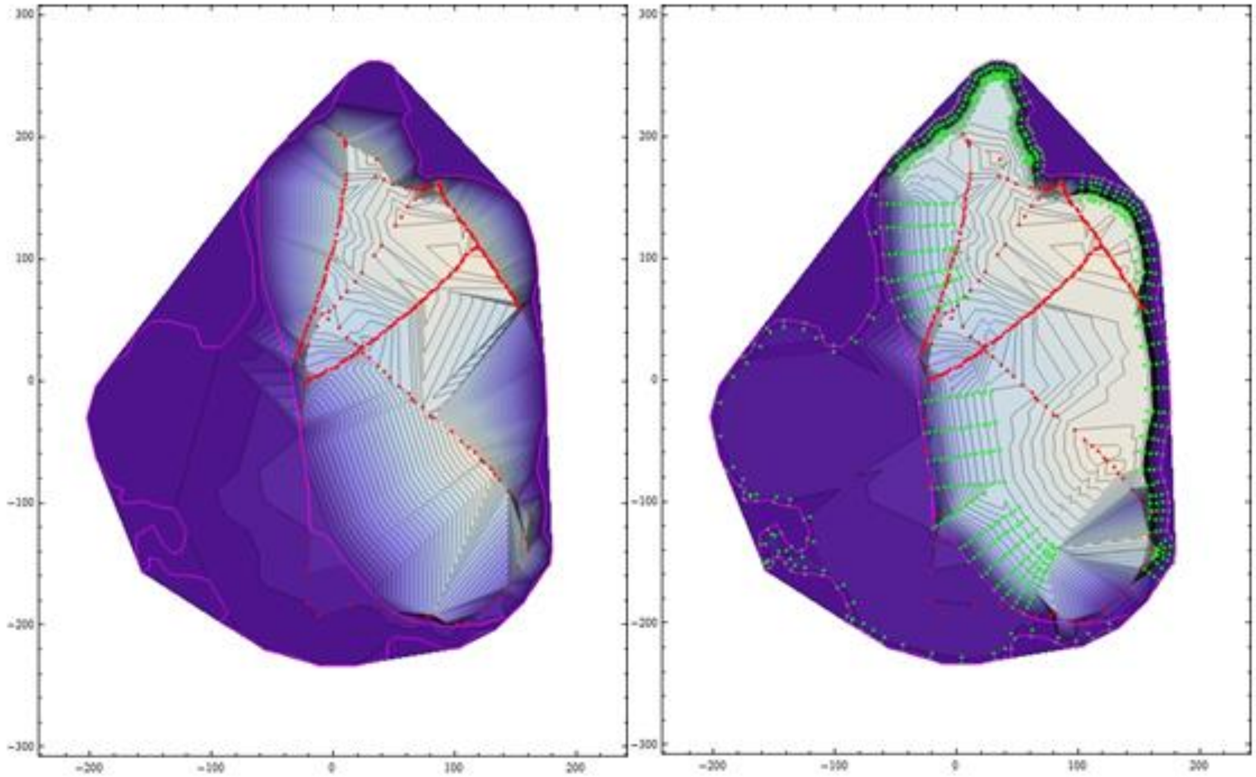

Figure S52. Above: The bathymetry of Lib Pond in 2D contours. A "conventional" interpolation is shown to the left. Note the sharp triangular shapes. The results of the boundary slope fitting can be seen on the right. Green points are the modeled data points, placed on edges of boundaries where pond slope did not have GPS depth data. The green points on the shallow floodplain section are 0.2 m to ensure a uniform value across the floodplain region. In both cases red points are the GPS depth points. Lighter background colors indicate greater depths.

3D Bathymetric Map Difference, Standard Interpolation vs. Boundary Slope Fitting

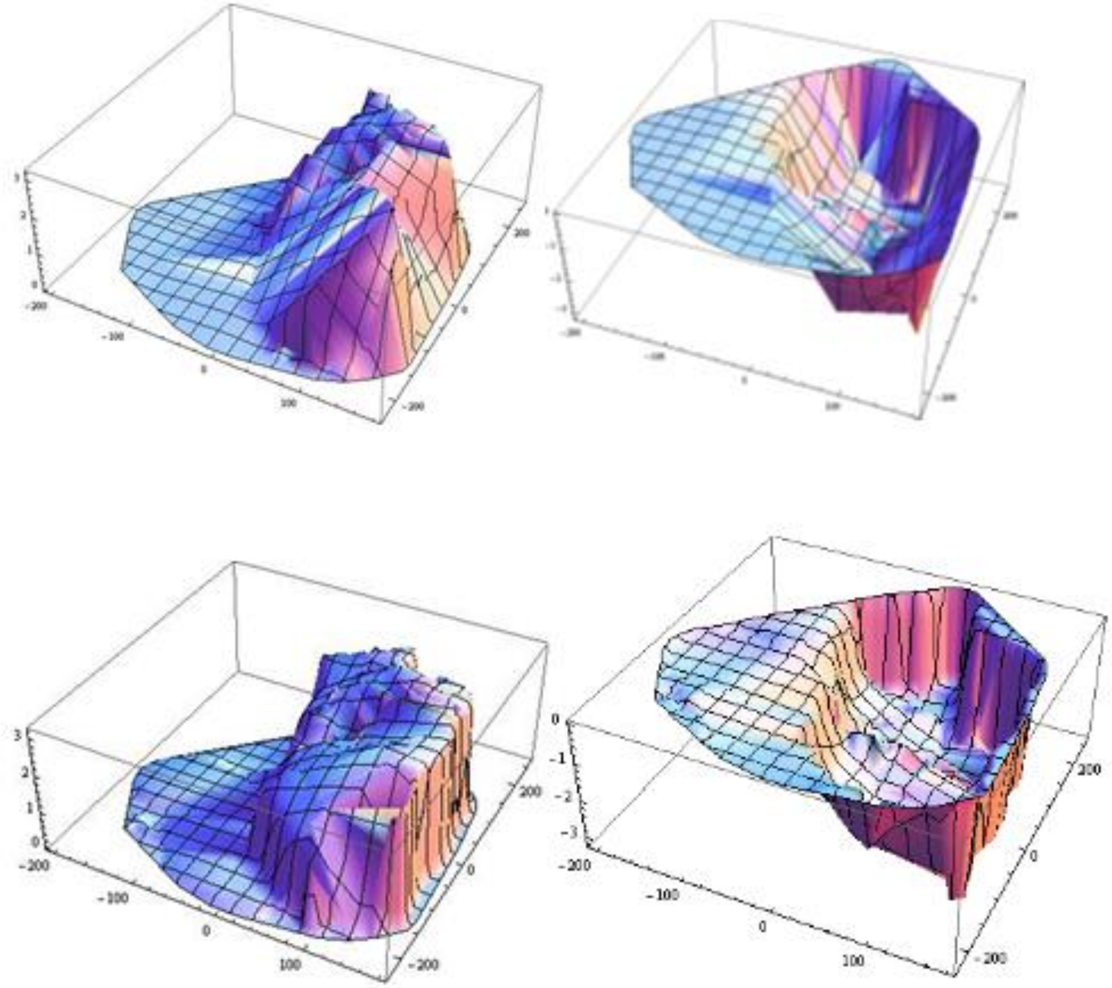

Figure S53. A 3D graphic showing the bathymetry of Lib Pond using a standard contour plot (above) and the boundary slope fitting method (below). Note that the z-axis is flipped in on the left hand side to better show bottom changes, <u>and is exaggerated 30x to show bottom detail</u>.

Triangular Surface Plot Comparison, Standard Interpolation vs. Boundary Slope Fitting

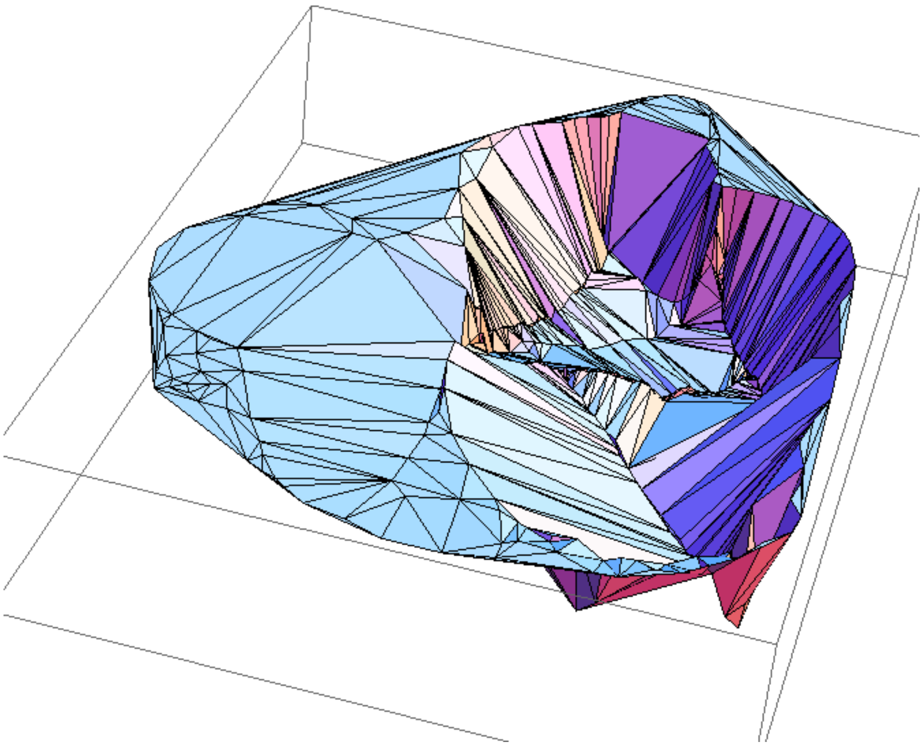

Figure S54. Conventional interpolation.

Lib Pond bathymetry displayed using a triangular surface plot. Note the flipped z axis on the left hand side. A conventional interpolation method is shown above while the results of boundary slope fitting are shown in the lower panels. A triangular mesh surface is fit to the depths of Lib Pond after the modeled depth data has been combined with the GPS depth data. (Note the 30x vertical exaggeration to better see the depth changes across the pond bottom, in all cases. The figure scales differ slightly between the two interpolations.)

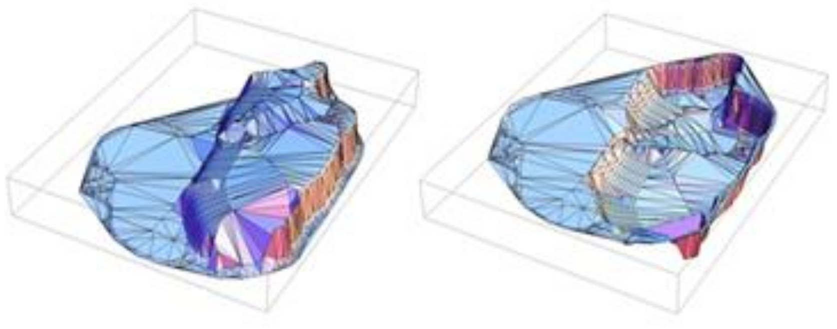

Figure S55. Interpolation after triangular mesh through boundary slope fitting

(Note the 30x vertical exaggeration).

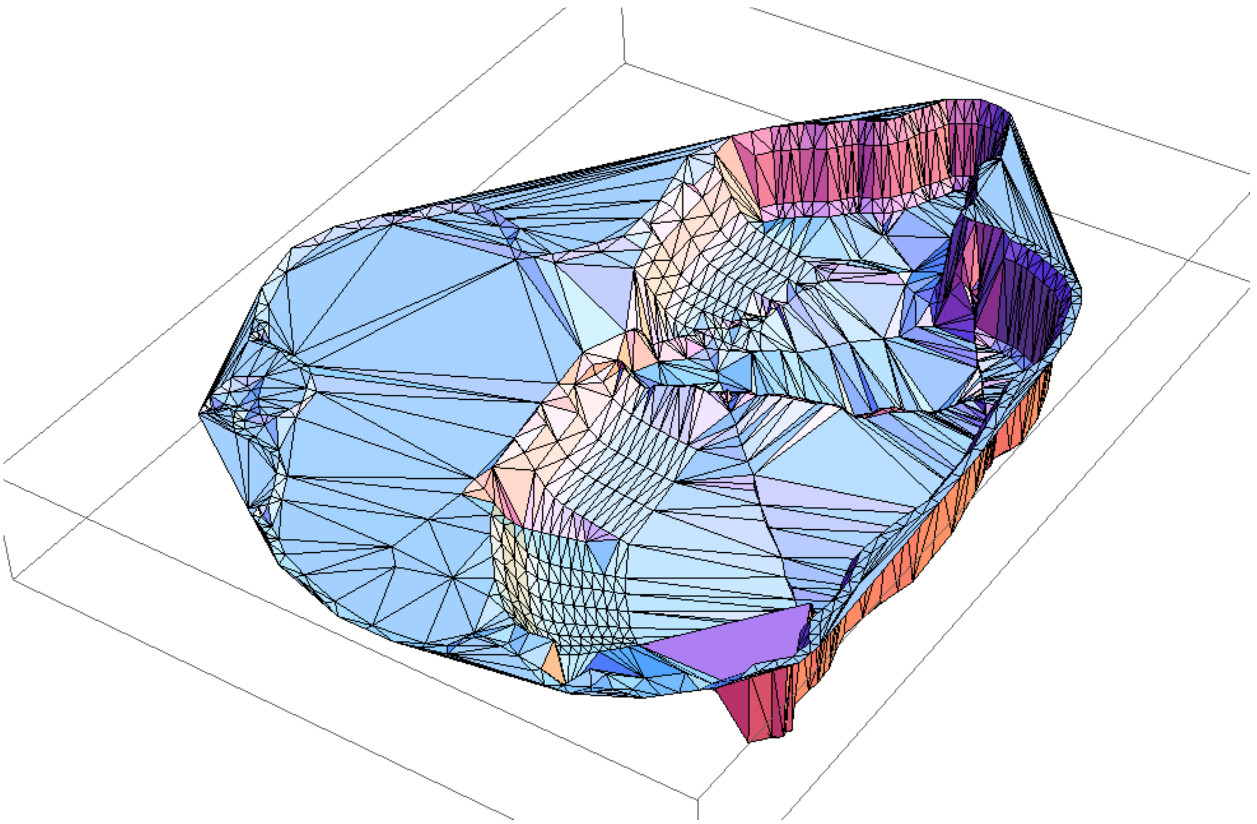

Black and White Luminosity vs. Depth (Remote Sensing)

As another possible way to gauge the depths of Lib Pond, the satellite image was converted from color to monochrome. The idea is that the original depth data were taken across pixels with different luminosities, and that a correlation between depth and pixel value might exist.

The color satellite image of the pond was converted to gray scale to see if there was a correlation between depth and color. But in this case there was no apparent correlation when pixel luminosity values are plotted versus the depth data. There was no correspondence between the color and the depth. As you can see from the graph on the right, there is little correlation, indicated by the wide variation of depths associated with each value of pixel luminosity. This is likely due to the different bottom types and specular reflection of sunlight off the surface of the pond.

Therefore, remote sensing is not helpful in further determining the depth of Lib Pond. This is not surprising since color differences in the water column are likely due to bottom type change or particulate and organic matter, rather than by depth change.

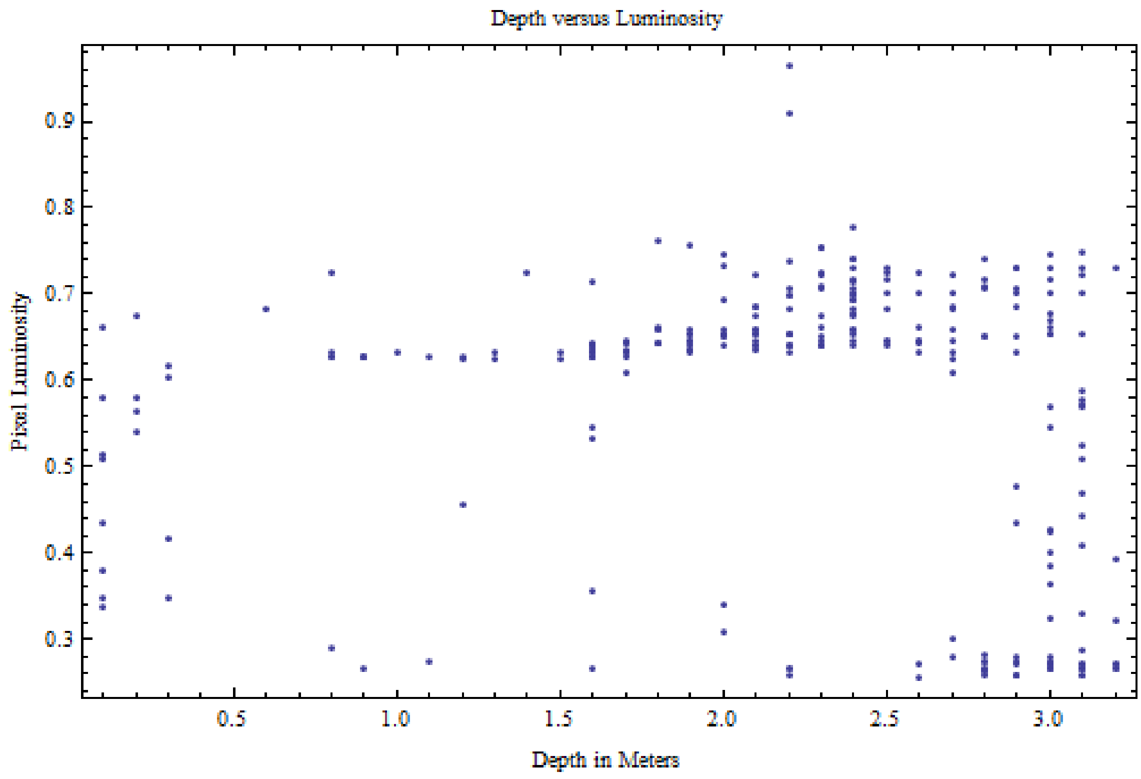
Depth versus Luminosity
Pixel Luminosity
0.3
0.4
0.5
0.6
0.7
0.8
0.9
0.5
1.0
1.5
2.0
2.5
3.0
Depth in Meters

Figure S56.

Figure S57. Boundary Slope Fitting Step by Step Process

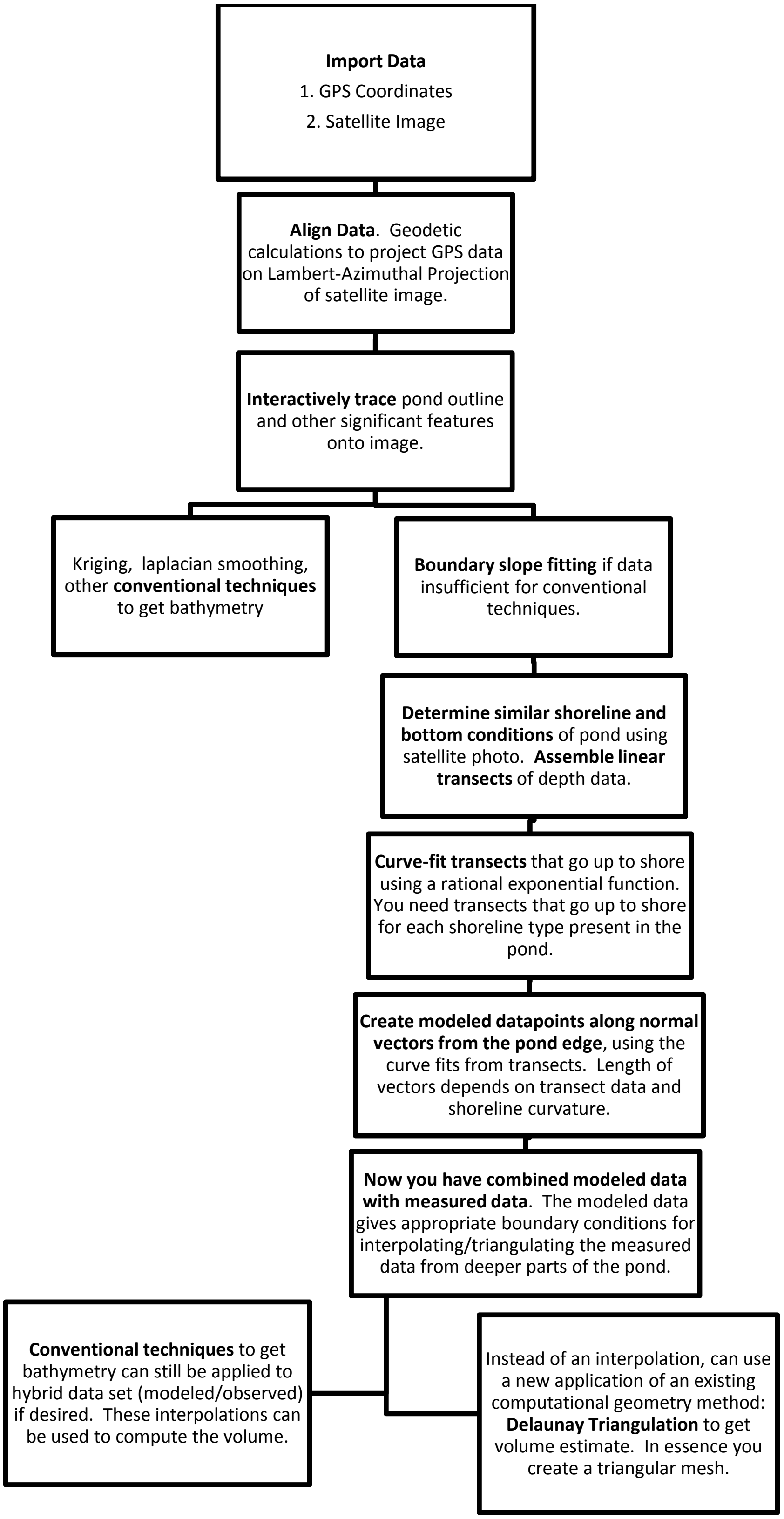

Volume Calculation Discussion

There are additionally several ways to calculate the lake volume after a bathymetric map is created. One could take modeled data and use contour plotting, kriging or other statistical techniques to calculate the volume but we used Delaunay triangulation. It and other computational geometric methods are widely used in finite element modeling and computer graphics, both of which require modeling a surface with a triangulated mesh at a high level of accuracy, and are a particular way of taking a set of data points in 2D and dividing them up into a set of triangles [15] [16].

Results from Delaunay triangulation were compared to a conventional kriging technique as implemented in ArcGIS. To better visualize the difference between the original depth data and the added shoreline points (i.e. modeled data) – a difference map was created from a kriging interpolation done in ArcGIS after boundary slope fitting was applied. The volume difference between a Delaunay triangulated surface and kriging is negligible for the original data point set and the modeled one.

Table S11. Pond Volumes via Different Methods

Volume Method (Software Used)	Extra Depth Points?	Volume (m^3)	Comparison (%)
Kriging (ArcGIS)	None	137,178	+ 1.3 from Delaunay to kriging
Delaunay Triangulation (Mathematica)	None	135,396	+ 1.3 from Delaunay to kriging
Delaunay Triangulation (Mathematica)	Boundary Slope Fitting	169,900	+1.4 from kriging to Delaunay
Kriging (ArcGIS)	Boundary Slope Fitting	167,439	+1.4 from kriging to Delaunay

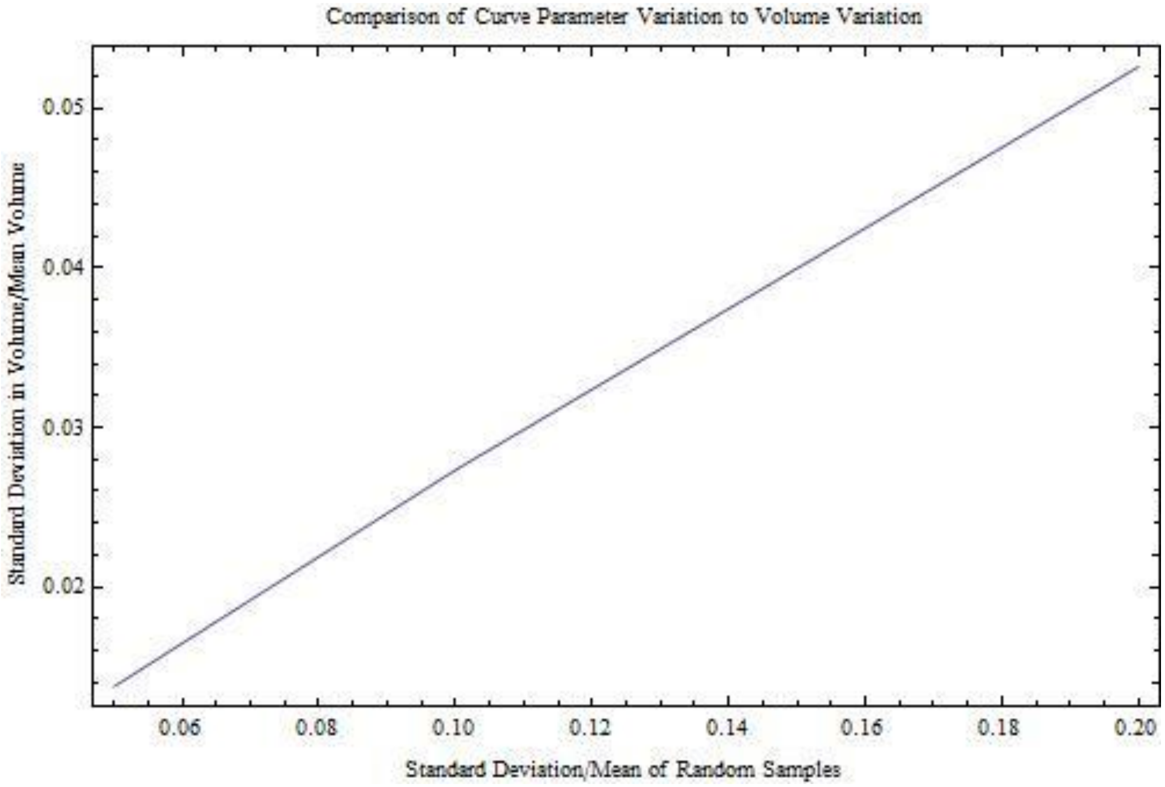

Figure S58.

The boundary slope fitting variation compared against the volume variation. This is done by putting the $\frac{std\ dev}{mean}$ for the volume and the curve fitting, and plotting the ratios against each other on different axes, for 5,10, and 20% standard deviations. Note that curve fitting variation is the parameter variation, since the parameters compose, and can be derived from, the curve fits. This graph shows that there is roughly 4:1 ratio of curve fitting error to volume error. In other words, if the curve fitting error is known to be 10%, then the resulting volume error from the curve error would only be 2.5%. So even if the curve fits were off (highly unlikely; see prior analysis) the pond volume would not be off by much as the curve fitting is off. The fit to the line is: $0.001175 + 0.2577x$.

Bathymetric maps comparing kriging using the original dataset (right) and the dataset including the modeled points with boundary slope fitting (left). The depth transitions are more realistic with the addition of modeling, as seen from the color display.

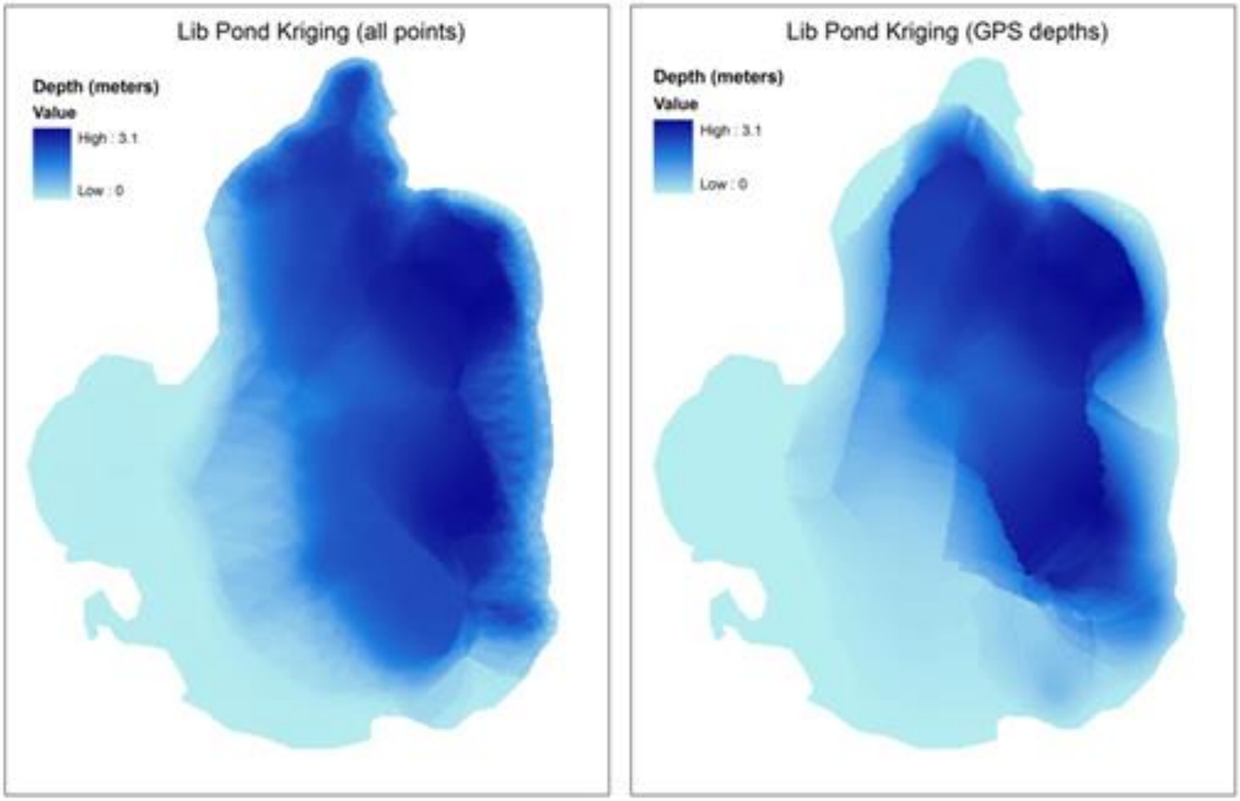

Figure S59.

A comparison of the bathymetric map of the original data (right) versus including the modeled data points (left), shown using a Triangulated Irregular Network (TIN) created in ArcGIS. The region beyond the channel is visible on the left but not the right, demonstrating that boundary slope fitting makes the resulting depths adhere to the physical properties of the pond.

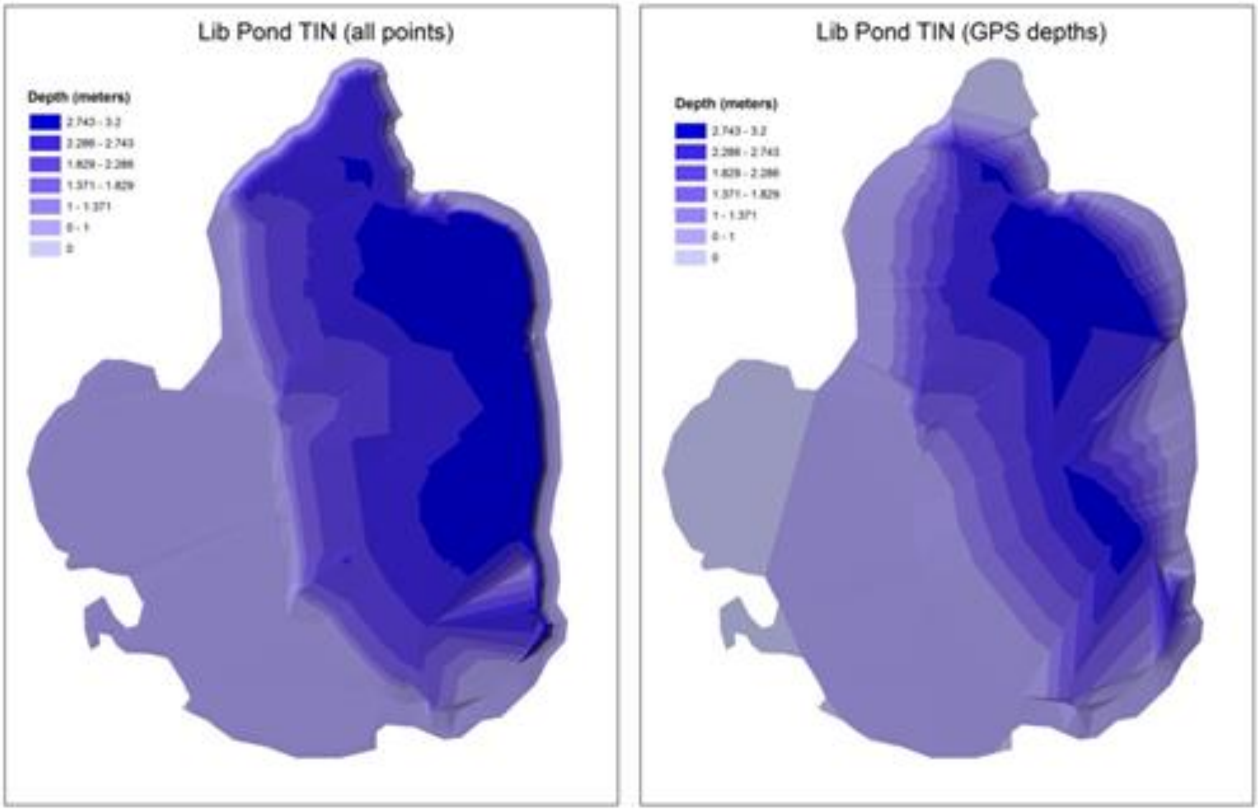

Figure S60.

A difference map comparing depths using kriging with their corresponding depths using the bathymetric map from Delaunay Triangulation. The point is to see adding the boundary slope fitting points versus not having the boundary slope fitting points. The depth values at each location for the original data set were subtracted from all of the points. Thus, it is all data points (including modeling) minus the original data points. Positive values indicate that the boundary slope fitting data set depth is deeper than the original data set. Negative values indicate that interpolation from the boundary slope fitting data set has a shallower value than the original data set. The map is color coded according to scale from 2.4 meters deeper to 1 meter shallower. White areas indicate no change. (Ignore the color outside the boundary).

The map is shown on the next page:

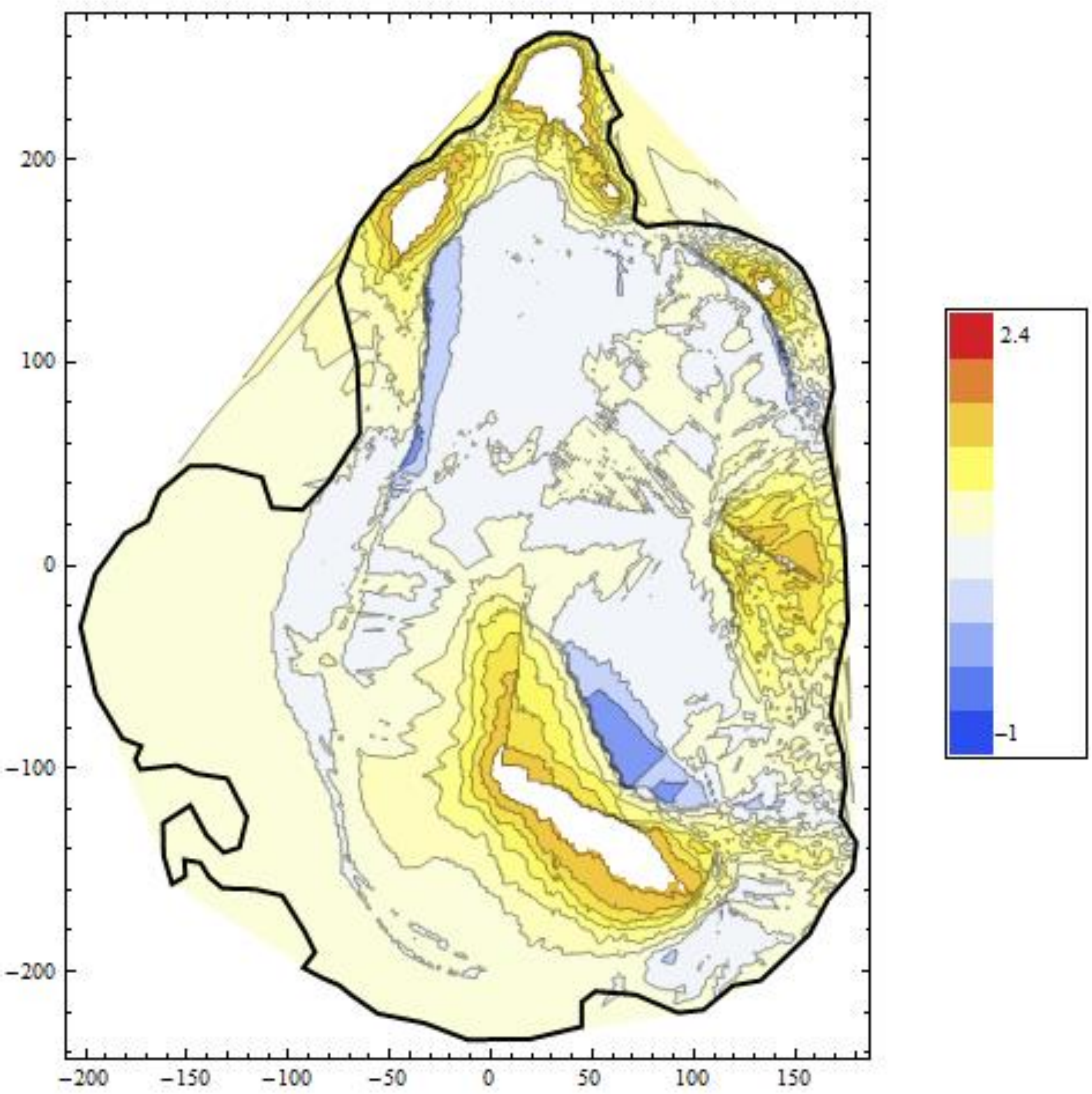

Figure S61.

Error Analysis of Volume Estimation

An error analysis of the data and methods is presented to demonstrate the validity the boundary slope fitting method and the volume and bathymetry of Lib Pond. First, there is error in the raw data used to compute the lake bathymetry (GPS coordinates and associated depths, located in the Bathymetric Data section of the Supplementary Online Material). This error is an inherent characteristic of data collecting and thus of all bathymetric and volume estimation methods. In certain situations, GPS error can lead to critical misinterpretations of GPS data [17], but we show that the GPS error for Lib Pond has a negligible effect on the volume calculation (and thus implicitly, the bathymetric map). Replications of the GPS coordinates with introduced error produce a histogram of volumes with a low standard deviation.

Each GPS coordinate was randomly scattered according to an error distribution. The Monte Carlo simulation of 500 trials, which produced 500 different lake volumes, had a mean volume value of 170,205 m^3 and a standard deviation of 594.474 m^3. Thus, lake volume did not vary substantially due to GPS error.

A larger error source comes from the method used to estimate the volume (in this case boundary slope fitting). To test the effect of variance on volume, Gaussian distributed random parameters for the exponential function curve fits were added. (Gaussian noise with a mean of zero and a standard deviation of 10% of the value found in the curve fit for each parameter a,b,c, and d). This results in 10% in the slope parameter, in the depth parameter, etc., quite a large cumulative error to apply, as seen in the resulting slope differences.

The modeled error is far higher than the actual residuals from boundary slope fitting and is intended to simulate lake regions not well-approximated by nearby transect data. Again, a Monte Carlo simulation of 500 trials produced 500 lake volume values with a mean of 170,040

m^3 and a standard deviation of 4648.33 (substantially higher than the GPS error but nevertheless relatively low.) Expressed as a percentage, the ratio of the standard deviation divided by the mean lake volume is 2.7%. Despite a simultaneous 10% difference in each parameter – such as curve slope and length – the end lake volume was essentially identical. Monte Carlo results using parameter standard deviations of 5% and 20% had similar effects to the 10%.

The effect of boundary slope fitting on the resulting volume can be quantified by a ≈ 4:1 ratio when the boundary slope fitting errors are plotted against the volume error. In other words, one quarter of the error from the curve fit gets passed onto the volume calculation. This is consistent with the fact that the boundary slope fitting method only added about 25% of the volume of the lake. The volume calculation is insensitive to boundary slope fitting due to modeling shallow regions of the lake as opposed to deep regions.

Finally, any echo sounder depth reading will have penetration error. Hence, the definition of the bottom using sonar is dependent on sediment porosity, and varies depending on echo sounder strength and frequency. Thus knowing the specifications of the echo sounder, including the measurement resolution, is more important for shallow bodies of water as than the open ocean.

Table S12. Volume Error Estimates

Lib Pond Volume Estimate (m^3)	Modeled Errors	Error Type using Monte Carlo Simulations
170,205 ± 594	---	GPS error, standard gamma distribution.
169,977 ± 2345	5%	Errors in boundary slope fitting: zero mean, 5% SD
170,040 ± 4648	10%	Errors in boundary slope fitting: zero mean, 10% SD
170,317 ± 8954	20%	Errors in boundary slope fitting: zero mean, 20% SD

Volume estimates from error analysis of Delaunay triangulation volume method, boundary slope fitting data set (i.e. original value used was 169,900 m^3.)

A typical GPS gamma error distribution. A similar figure is seen in [17].

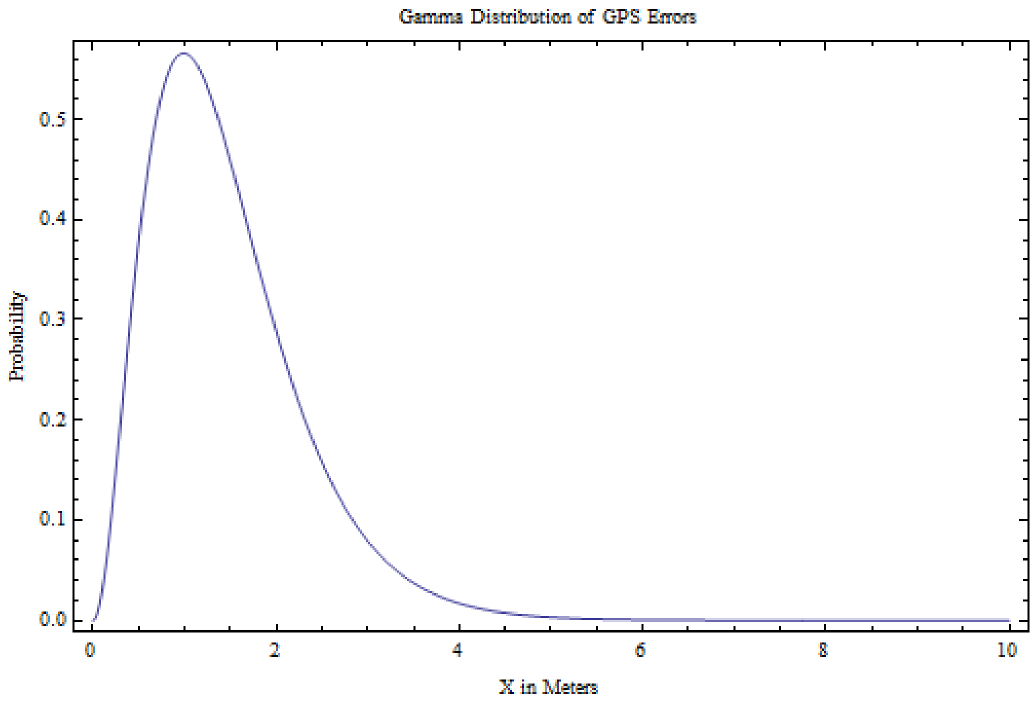

Figure S62.

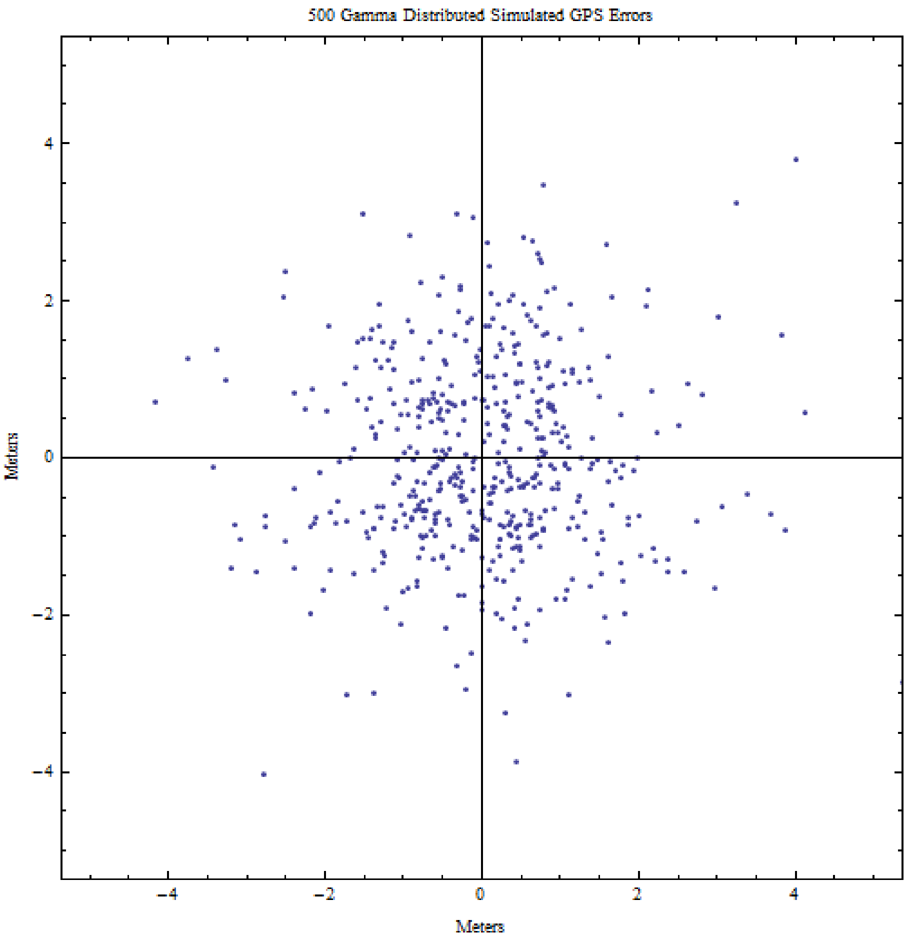

Figure S63. Sources: http://users.erols.com/dlwilson/gpsacc.htm **,**
http://www.ion.org/search/view_abstract.cfm?jp=p&idno=4763

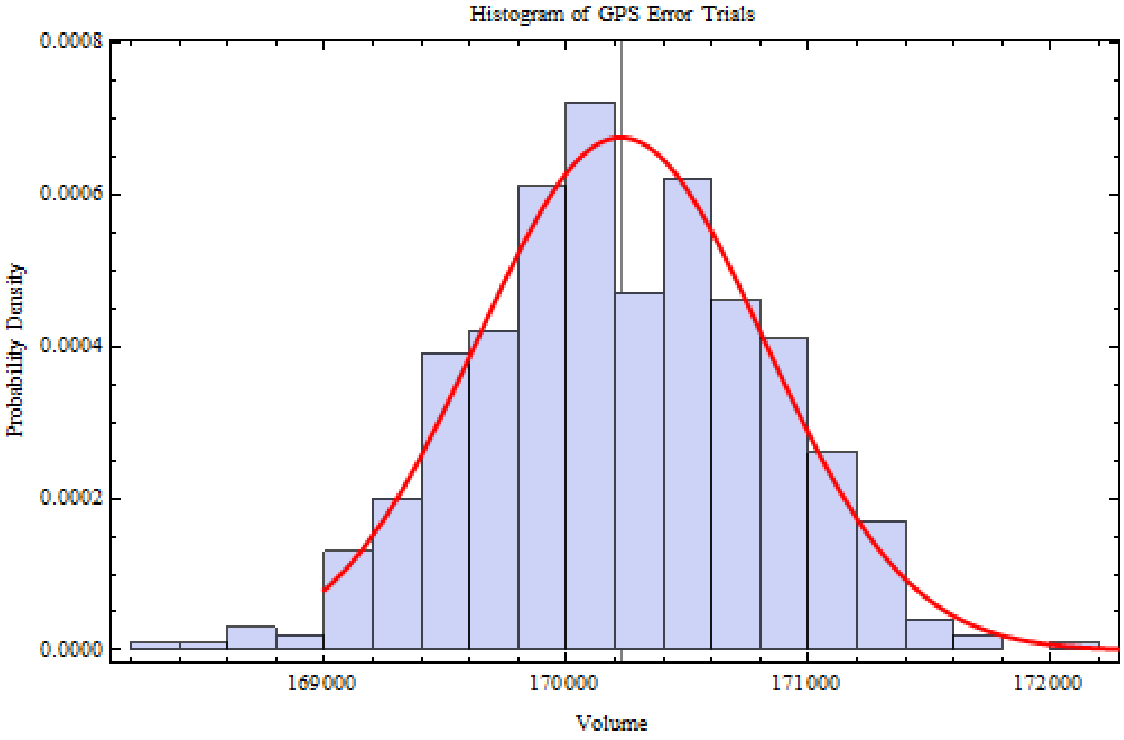

Figure S64. GPS error is negligible. The histogram fits a Gaussian curve. Volume is in cubic meters (m^3).

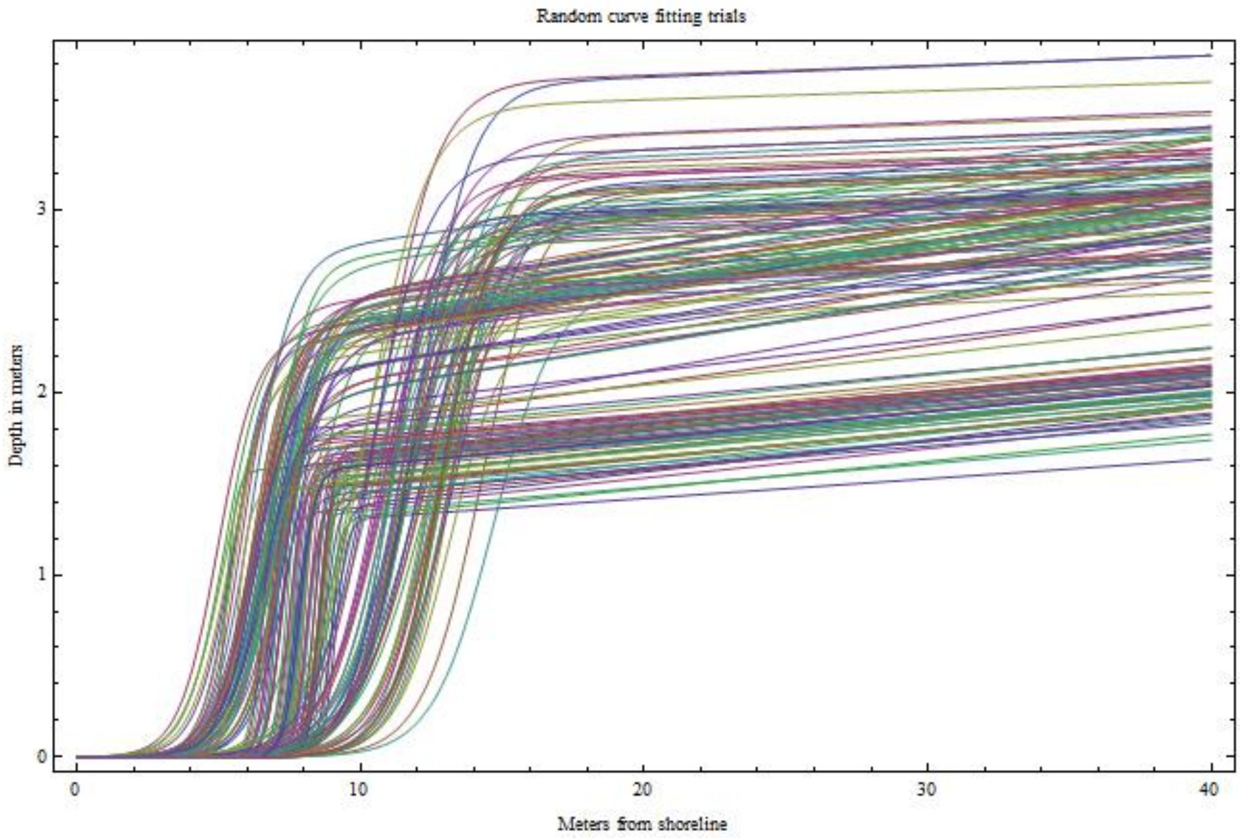

Figure S65. 500 curves fit to exponential functions. Each input of the function varied by a standard deviation of 10%.

The three graphs below show that the boundary slope fitting error is relatively small. The histograms fits a Gaussian "bell" curve. Volume is in cubic meters (m^3). (**Figure S66**) Using 10% standard deviations. The same boundary slope fitting variation compared against the volume variation, done for 5% deviations (**Figure S67**) and 20% deviations (**Figure S68**) of the parameters. The R^2 value of the 5% SD is 0.999934, for example.

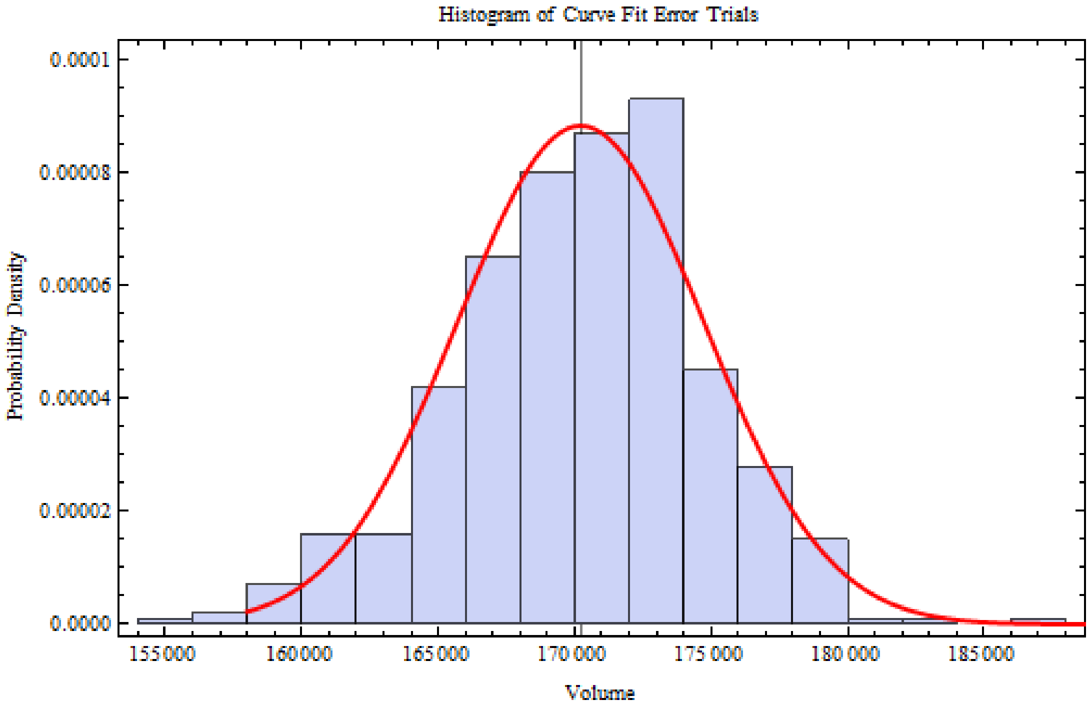

Figure S66.

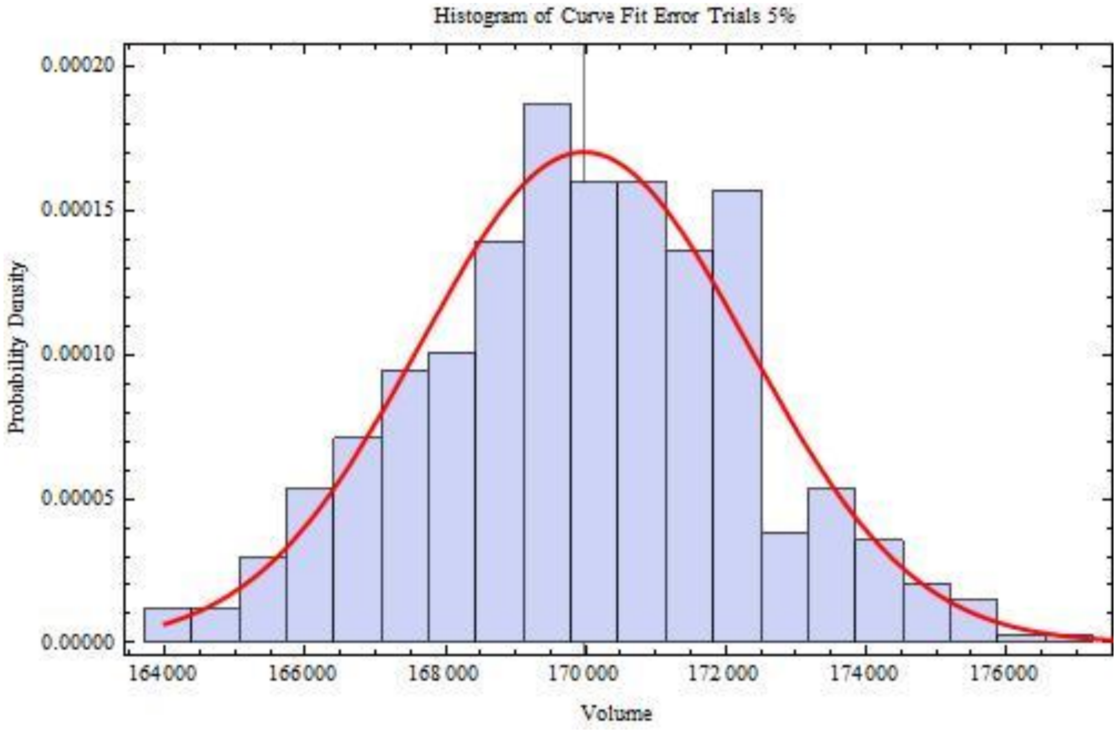

Figure S67 (above)**. Figure S68** (below)**.**

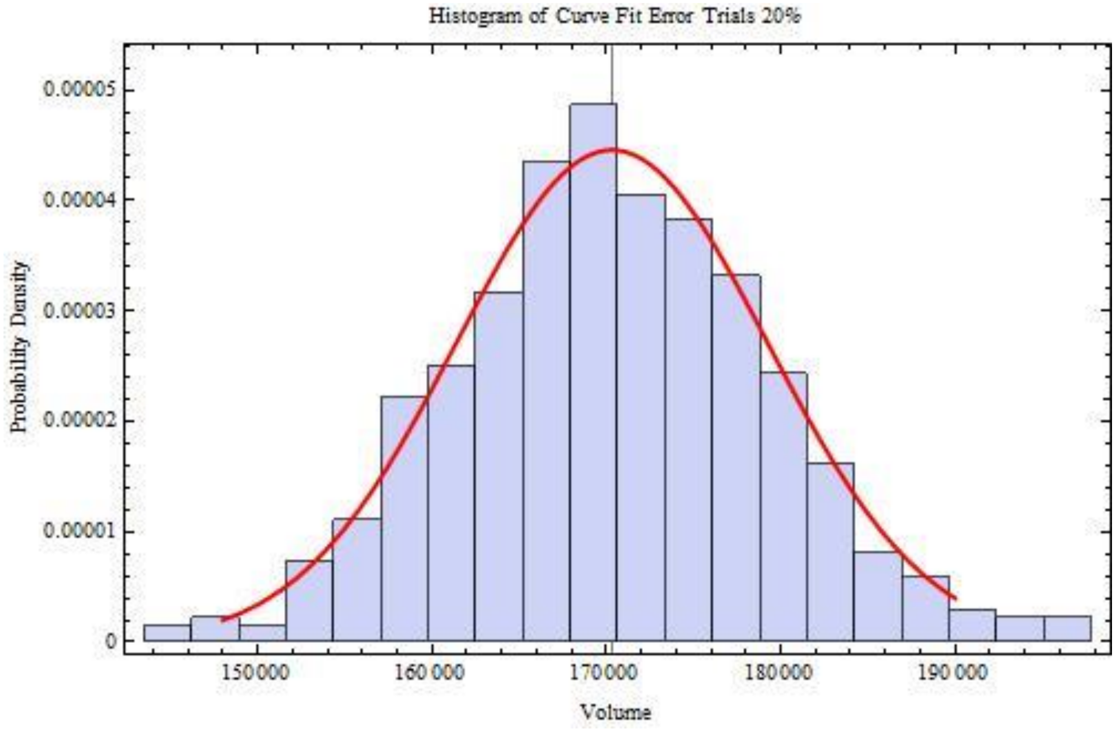

Water Volume Estimation

The calculated Lib Pond volume increases by ~25% modeling the bathymetry using boundary slope fitting. Computing the volume after kriging versus Delaunay triangulation makes little difference to the resulting volume. However, the lake bottom consists of extremely porous sediment so the volume estimate only accounts for water outside of the sediment, what can be called the open water column.

Therefore, taking the sediment porosity into account when calculating the water volume the system contains is a high remaining source of uncertainty. Since 8.5+ m of sediment were recovered at the deepest point, of which the first 4 m were very porous (> 0.5, or 50% water), the total lake sediment volume is likely greater than the open water volume. A single value of porosity is likely appropriate for the entire sediment column, since the depth is on the order of meters (8 m) instead of a km (i.e. 1-2 km, which would be modeled linearly) or many kms (i.e. 5-10 kms, which would be modeled exponentially) [18], but permutations involving an exponential drop off in porosity should also be tested.

An extremely porous orange gelatinous sediment layer that was 90%+ water was likely represented as part of the water column since the echo sounder "read" through it. Field experiments demonstrated that the depth sounder was inaccurate and overestimated depth on the order of 0.5 m when tested in shallow water (< 1 m, where the bottom could be visualized.) Thus, the current estimate for lake water volume is almost certainly an upper bound. Multiplying the surface area of the lake by 0.5 m and subtracting it from the volume estimate subtracts 60,346 m^3 (ignoring significant figures, for the sake of the example). For boundary slope fitting using Delaunay triangulation, the volume would change from 169,900 m^3 to 109,554 m^3, a

baseline volume that is almost certainly a lower bound. Therefore a range of Lib Pond water volumes is ~110,000 m^3 to ~170,000 m^3.

Thus the current estimated lake volume is really an open water volume calculation; there could be a substantial amount of water in the sediment. Thus the current state of Lib Pond is consistent with a lake in a late stage of sediment accumulation that is filling in. We believe the shallow floodplain region will eventually expand to cover the entire lake based on the high sediment accumulation rates inferred from the Lib Pond sediment cores, which would reduce the volume estimation of open water in Lib Pond.

References for Supporting Information

1. Sachs JP, Sachse D, Smittenberg RH, Zhang Z, Battisti DS, Golubic S (2009) Southward movement of the Pacific intertropical convergence zone AD1400-1850. Nat Geosci 2: 519–525. Available: http://dx.doi.org/10.1038/ngeo554.

2. Zhang Z, Sachs JP (2007) Hydrogen isotope fractionation in freshwater algae: I. Variations among lipids and species. Org Geochem 38: 582–608. Available: http://linkinghub.elsevier.com/retrieve/pii/S0146638006003019.

3. Gat JR (1996) Oxygen and Hydrogen Isotopes in the Hydrologic Cycle. Annu Rev Earth Planet Sci 24: 225–262.

4. Sachse D, Sachs JP (2008) Inverse relationship between D / H fractionation in cyanobacterial lipids and salinity in Christmas Island saline ponds. 72: 793–806. doi:10.1016/j.gca.2007.11.022.

5. Saenger C, Miller M, Smittenberg RH, Sachs JP (2006) A physico-chemical survey of inland lakes and saline ponds : Christmas Island (Kiritimati) and Washington (Teraina) Islands , Republic of Kiribati. Saline Systems 15. doi:10.1186/1746-1448-2-8.

6. Hamner WM, Gilmer RW, Hamner PP (1982) The physical, chemical, and biological characteristics of a stratified, saline, sulfide lake in Palau. Limnol Oceanogr 27: 896–909.

7. Fosberg FR (1990) A review of the natural history of the Marshall Islands. Atoll Res Bull: 1–100.

8. Kwajalein Climate Summary (2007) RTS Weather Stn. Available: http://www.rts-wx.com/climatology/summary/. Accessed 22 January 2013.

9. Helvécio D, Estadual P, Perd RD, Gerais M (2008) Morphometric study of Lake Dom Helvécio, Parque Estadual do Rio Doce (PERD), Minas Gerais, Brazil : a re-evaluation. Acta Limnol Bras 20: 161–167.

10. Meyer TH (2004) The Discontinuous Nature of Kriging Interpolation for Digital Terrain Modeling. Cartogr Geogr Inf Sci 31: 209–216.

11. Vollmer J, Mencl R, Müller H (1999) Improved Laplacian Smoothing of Noisy Surface Meshes. Eurographics ’99 18.

12. Wilcox C, Huertos ML (2005) A simple, rapid method for mapping bathymetry of small wetland basins. J Hydrol 301: 29–36. doi:10.1016/j.jhydrol.2004.06.027.

13. Bills BG, Borsa AA, Comstock RL (2007) MISR-based passive optical bathymetry from orbit with few-cm level of accuracy on the Salar de Uyuni, Bolivia. Remote Sens Environ 107: 240–255. doi:10.1016/j.rse.2006.11.006.

14. Mcintyre ML, Naar DF, Carder KL, Donahue BT, Mallinson DJ (2006) Coastal bathymetry from hyperspectral remote sensing data : comparisons with high resolution multibeam bathymetry. Mar Geophys Res 27: 129–136. doi:10.1007/s11001-005-0266-y.

15. Fragakis Y, Eugenio O (2008) Parallel Delaunay triangulation for particle finite element methods. Commun Numer Meth Engng 24: 1009–1017. doi:10.1002/cnm.

16. Silveira RI (2009) Optimization of polyhedral terrains door, Utrecht University.

17. Hurford A (2009) GPS Measurement Error Gives Rise to Spurious 180u Turning Angles and Strong Directional Biases in Animal Movement Data. PLoS One 4. doi:10.1371/journal.pone.0005632.

18. Bahr DB, Hutton EWH, Syvitski JPM, Pratson LF (2001) Exponential approximations to compacted sediment porosity profiles. Comput Geosci 27: 691–700.
